# Wnt/β-catenin coupled with HIF-1α/VEGF signaling pathways involved in galangin neurovascular unit protection from focal cerebral ischemia

**DOI:** 10.1038/srep16151

**Published:** 2015-11-05

**Authors:** Chuanhong Wu, Jianxin Chen, Chang Chen, Wei Wang, Limei Wen, Kuo Gao, Xiuping Chen, Sihuai Xiong, Huihui Zhao, Shaojing Li

**Affiliations:** 1 Institute of Chinese Materia Medica, China Academy of Chinese Medical Sciences, Beijing 100700, China; 2 State Key Laboratory of Quality Research in Chinese Medicine, Institute of Chinese Medical Sciences, University of Macau, Macao, 999078, China.; 3 Beijing University of Chinese Medicine, Beijing 100029, China.; 4 The first Affiliated Hospital of Xinjiang Medical University, Xinjiang, 830054, China.; 5 Beijing No.166 High School, Beijing 100006, China

## Abstract

Microenvironmental regulation has become a promising strategy for complex disease treatment. The neurovascular unit (NVU), as the key structural basis to maintain an optimal brain microenvironment, has emerged as a new paradigm to understand the pathology of stroke. In this study, we investigated the effects of galangin, a natural flavonoid isolated from the rhizome of *Alpina officinarum* Hance, on NVU microenvironment improvement and associated signal pathways in rats impaired by middle cerebral artery occlusion (MCAO). Galangin ameliorated neurological scores, cerebral infarct volume and cerebral edema and reduced the concentration of Evans blue (EB) in brain tissue. NVU ultrastructural changes were also improved by galangin. RT-PCR and western blot revealed that galangin protected NVUs through the Wnt/β-catenin pathway coupled with HIF-1α and vascular endothelial growth factor (VEGF). VEGF and β-catenin could be the key nodes of these two coupled pathways. In conclusion, Galangin might function as an anti-ischemic stroke drug by improving the microenvironment of NVUs.

Ischemic stroke results from sudden disruption of blood flow to the brain, leading to brain cell death and neurological deficits. Considering the complex mechanism involved in the pathological process of ischemic stroke, drugs only acting on a single target might not be sufficient to treat ischemic stroke. In 2002, the neurovascular unit (NVU) was proposed as a structural and functional unit of the nervous system as part of an integrated model for the treatment of stroke^1^. The NVU couples neuronal activity to vascular function, controls brain homeostasis, and maintains an optimal brain microenvironment adequate for neuronal survival by adjusting blood-brain barrier (BBB) parameters based on the brain’s needs^2^. The structural basis of the NVU is the neurons, BBB, microglial cells and extracellular matrix that maintain the integrity of brain tissue. The BBB is the core structure of NVU, consisting of endothelium, the basal lamina matrix, the end-feet of astrocytes and pericytes^3^. Microtubule-associated protein-2 (MAP-2) and glial fibrillary acidic protein (GFAP) are classical proteins used for the identification of NVU components^4^. Coagulation factor VIII is an essential glycoprotein pro-cofactor within the intrinsic pathway of the blood coagulation cascade and is synthetized by the endothelium. The NVU model changed the concept of neuroprotection from only nerve protection to overall stabilization of the brain microenvironment, promoting neuronal survival, brain plasticity, and neurological recovery.

The BBB prevents many macromolecules and harmful substances from entering the brain. The injury of any BBB components caused by stroke could lead to an increase in its permeability^5^. The value of Evans blue (EB) in brain tissue is commonly used to evaluate the permeability of the BBB. Lanthanide metal penetration observed by transmission electron microscopy (TEM) is also used. The tight junction (TJ) is significant in maintaining the integrity of the BBB. The injury of TJs is marked with the expression of zonula occludens-1 (ZO-1) and claudin-5^6^.

Neurogenesis triggered by ischemia in the adult mammalian brain may provide insights into stroke treatment^7^. Accumulated evidence reveals that the Wnt/β-catenin pathway is a crucial pathway in the regulation of neurogenesis^8^,^9^. The Wnt/β-catenin pathway plays critical roles in embryonic development and adult homeostasis, including determination, proliferation, migration and differentiation. Alterations of Wnt signaling are involved in neuronal death and other human diseases^10^. Vascular endothelial growth factor (VEGF) is an important signaling molecule in angiogenesis and neurogenesis^11^. Hypoxia-inducible factor-1α (HIF-1α) acts upstream of the Wnt/β-catenin pathway and also may contribute to the production of VEGF^12^. Moreover, HIF-1α plays a role in brain edema formation and BBB disruption via a signaling pathway involving the glial water channel aquaporin-4 (AQP-4) and matrix metalloproteinase-9 (MMP-9)^13^.

Galangin is a natural flavonoid isolated from the rhizome of *Alpina officinarum* Hance that has been widely used as an antioxidant agent. It has multiple bioactivities and affects many cell systems. In addition to its anti-oxidant^14^, anti-obesity^15^, and anti-tumor effects^16^, galangin demonstrates anti-inflammatory^17^, anti-microbial^18^, and anti-viral^19^ activities in a variety of *in vitro* and *in vivo* systems. Furthermore, galangin has vasodilatory^20^, anti-ischemic, and anti-oxidant properties, which may reduce the risk of coronary heart disease and improve endothelial cell function^21^. Galangin inhibits acetylcholinesterase activity *in vitro* and might be of potential use for the treatment of Alzheimer’s disease^22^. Additionally, galangin modulates vascular smooth muscle Ca (v) 1.2 channels, which might be valuable in the treatment of hypertension and stroke^23^. In this study, we investigated the effects of galangin on NVU protection and its underlying mechanisms in MCAO rats.

## Results

### Effects of galangin on neurological defects, BBB integrity and NVU damage

**Figure 1:**
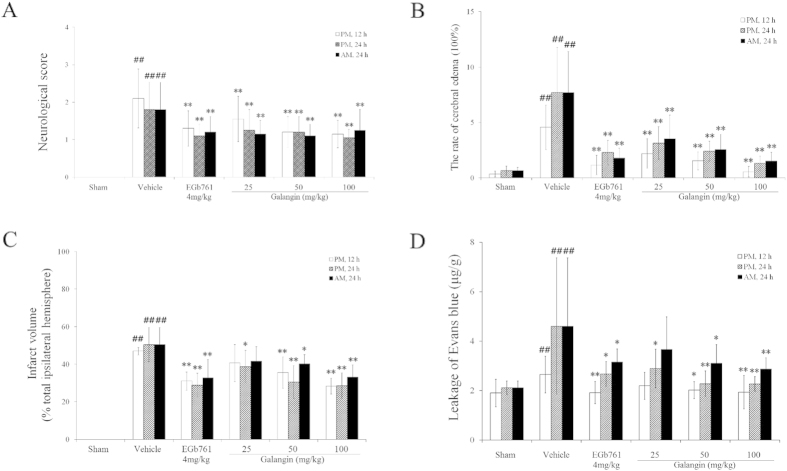
Effects of galangin on neurological defects and NVU damage. (**A**) Neurological deficits . (**B**) Cerebral edema. (**C**) Infarct volume. (**D**) Leakage of Evans blue. The values are expressed as the mean ± SD (n = 10), and the data were analyzed by one-way ANOVA. ^##^*P* < 0.01, ^#^*P* < 0.05 versus the sham group; ^**^P < 0.01, ^*^P < 0.05 versus the vehicle control.

Neurological defects are shown in Fig. 1A,C. The mean neurological scores in the galangin-treated groups were significantly improved (*P* < 0.01) compared to the vehicle group at all three time points. Galangin at the concentration of 50 mg•kg^−1^ and 100 mg•kg^−1^ reduced the infarct volume significantly (*P* < 0.01 or *P* < 0.05).

BBB integrity is reflected by cerebral edema and leakage of EB. As shown in Fig. 1B,D, the cerebral edema in the galangin-treated groups was significantly improved (*P* < 0.01) compared with the vehicle group at all three time points. Galangin at the concentration of 50 mg•kg^−1^ and 100 mg•kg^−1^ markedly reduced the leakage of EB (*P* < 0.01 or *P* < 0.05).

**Figure 2:**
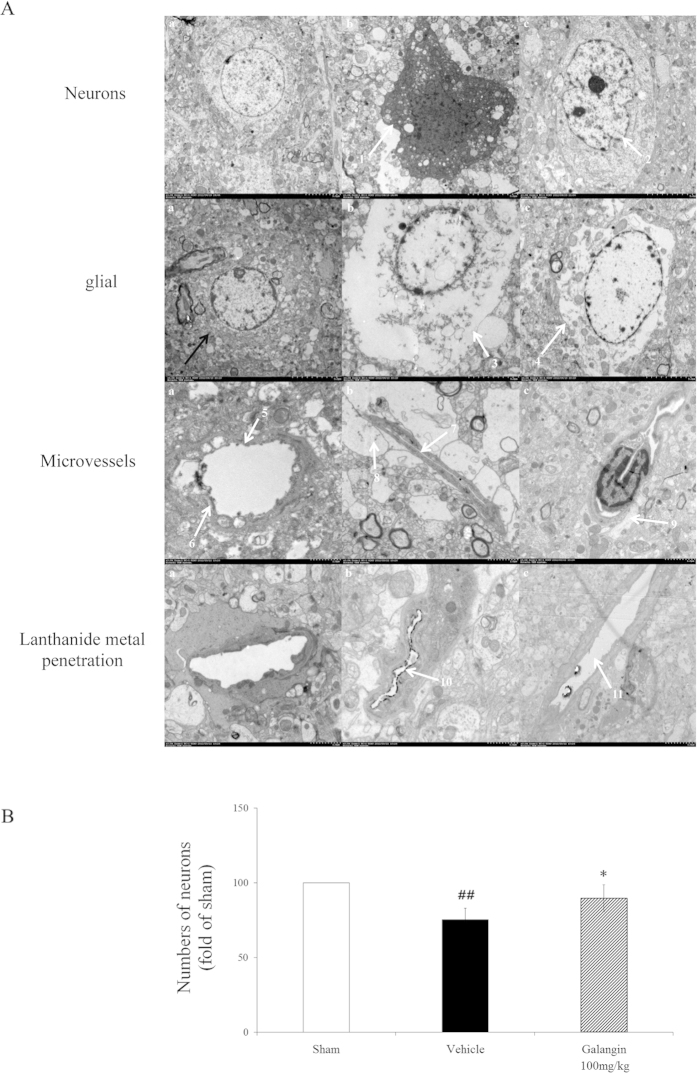
Effects of galangin on ultrastructural changes to NVU structure and BBB permeability. (**A**) Representative electron photomicrographs. (a) Sham group, with normal cell morphology, microvascular endothelial cells, basal lamina (**5**) and astrocyte end-feet (6). (b) Vehicle group: Neuron nuclei are condensed and the electron density of the neurons is increased (1). The glial cell had serous edema (3). Microvascular lumina was crushed and became narrow (7), end-feet of astrocyte were serous edema (8). Lanthanide metal penetrated into the microvascular lumina (10). (**c**) Galangin 100 mg•kg^−1^ group: The injury to neurons (2) was lessened. The edema of glial cell (4) and end-feet of astrocytes were also mitigated (9). The penetration of lanthanide metal was decreased (11). (**B**) Statistical comparison of the numbers of neurons. The values are expressed as the mean ± SD (n = 3), and the data were analyzed by one-way ANOVA. ^##^*P* < 0.01, ^#^*P* < 0.05 versus the sham group; ^**^P < 0.01, ^*^P < 0.05 versus the vehicle control.

Ultrastructural changes to the BBB and BBB permeability are illustrated in Fig. 2. The neurons and glial cells had normal cell morphology in the sham group. The microvessels had normal endothelial cells, basal lamina and astrocyte end-feet (Fig. 2A. a). However, a series of pathological changes to the BBB were observed in the vehicle group: neuron nuclei condensed and electron density of the neuron increased; the glial cells and end-feet of astrocytes were swollen; microvascular lumina was crushed and became narrow; a large amount of lanthanide mental penetrated into the microvascular lumina (Fig. 2A. b). Galangin (100 mg·kg^−1^) decreased the edema of astrocyte end-feet and glial cells and also alleviated the injury of neurons. Moreover, the penetration of lanthanide mental was decreased (Fig. 2A. c,B). A comparison of the numbers of neurons is shown in Fig. 2B. After MCAO, the neuron number was decreased significantly (*P* < 0.01), but in the galangin-treated groups, the neuron number was much higher than the vehicle group (*P* < 0.05).

### Effects of galangin on factor VIII, ZO-1, claudin-5, VEGF, HIF-1α, β-catenin, LRP6 and Frizzled-1

**Figure 3:**
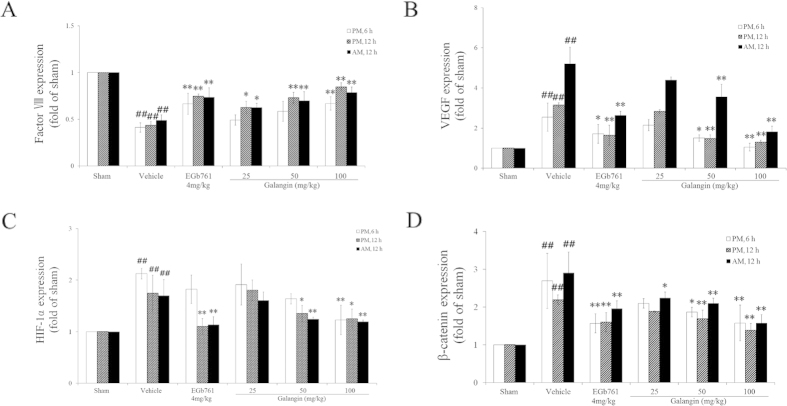
Effects of galangin on the mRNA expression of factor VIII, VEGF, HIF-1α and β-catenin. The values are expressed as the mean ± SD (n = 3), and the data were analyzed by one-way ANOVA. ^##^*P* < 0.01, ^#^*P* < 0.05 versus the sham group; ^**^P < 0.01, ^*^P < 0.05 versus the vehicle control.

**Figure 4:**
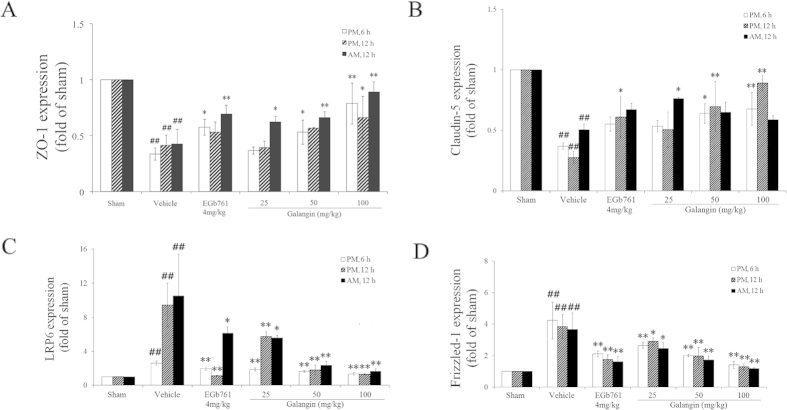
Effects of galangin on the mRNA expression of ZO-1, claudin-5, LRP6 and Frizzled-1. The values are expressed as the mean ± SD (n = 3), and the data were analyzed by one-way ANOVA. ^##^*P* < 0.01, ^#^*P* < 0.05 versus the sham group; ^**^P < 0.01, ^*^P < 0.05 versus the vehicle control.

As illustrated in Figs 3 and 4, RT-PCR showed that the mRNA expression of factor VIII, ZO-1, and claudin-5 decreased significantly (*P* < 0.01) after MCAO. The galangin-treated (50 mg·kg^−1^ and 100 mg·kg^−1^) groups and EGb761-treated group had improved expression of factor VIII, ZO-1 and claudin-5 (*P* < 0.01 or *P* < 0.05). VEGF, HIF-1α, β-catenin, LRP6 and Frizzled-1 in the vehicle-treated group had higher expression compared to the sham-treated group (*P* < 0.01). Galangin (50 mg•kg^−1^ and 100 mg·kg^−1^) and EGb761 down-regulated the expression of VEGF, HIF-1α, and β-catenin (*P* < 0.01 or *P* < 0.05). The expression of Frizzled-1 and LRP6 in the galangin-treated (25 mg•kg^−1^, 50 mg•kg^−1^ and 100 mg•kg^−1^) groups and EGb761-treated group were reduced significantly (*P* < 0.05 or *P* < 0.01).

### Effects of galangin on MAP-2, GFAP, AQP-4, MMP-9 and VEGF

**Figure 5:**
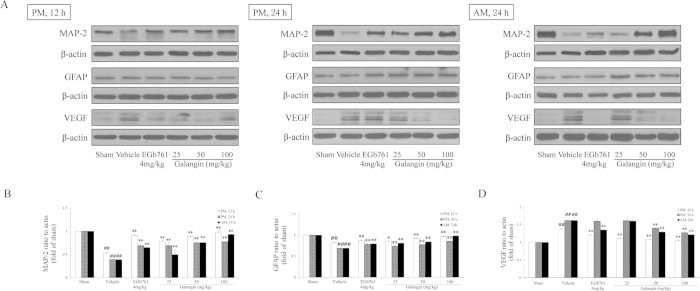
Effects of galangin on MAP-2, GFAP and VEGF expression in rat brain after MCAO. (**A**) Representative western blots of MAP-2, GFAP, VEGF and β-actin at three time points. (**B–D**) The quantified densitometric analysis of MAP-2, GFAP, and VEGF. The full-length blots are shown in Supplementary Materials. The values are expressed as the mean ± SD for the four independent experiments, and the data were analyzed by one-way ANOVA. ^##^*P* < 0.01, ^#^*P* < 0.05 versus the sham group; ^**^P < 0.01, ^*^P < 0.05 versus the vehicle control.

**Figure 6:**
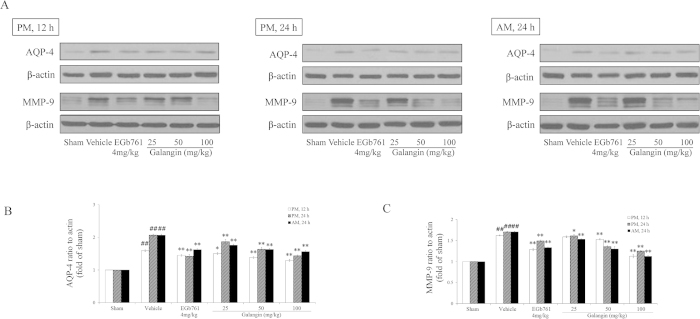
Effects of galangin on AQP-4 and MMP-9 expression in rat brain after MCAO. (**A**) Representative western blots of AQP-4, MMP-9 and β-actin in three time points. (**B–C**) The quantified densitometric analysis of AQP-4 and MMP-9. The full-length blots are shown in Supplementary Materials. The values are expressed as the mean ± SD for the four independent experiments, and the data were analyzed by one-way ANOVA. ^##^*P* < 0.01, ^#^*P* < 0.05 versus the sham group; ^**^P < 0.01, ^*^P < 0.05 versus the vehicle control.

GFAP, AQP-4, MMP-9 and VEGF were up-regulated in the vehicle-treated group (Figs 5 and 6). Galangin (25 mg·kg^−1^, 50 mg·kg^−1^ and 100 mg·kg^−1^) and EGb761 reduced the expression of GFAP, AQP-4, MMP-9 and VEGF (*P* < 0.01 or *P* < 0.05). In contrast, MAP-2 was down-regulated in the vehicle-treated group. Galangin (25 mg•kg^−1^, 50 mg•kg^−1^ and 100 mg•kg^−1^) and EGb761 up-regulated MAP-2 (*P* < 0.01).

### Effects of galangin on pGSK-3β, GSK-3β, pβ-catenin, β-catenin, pSmad3, and Smad3

**Figure 7:**
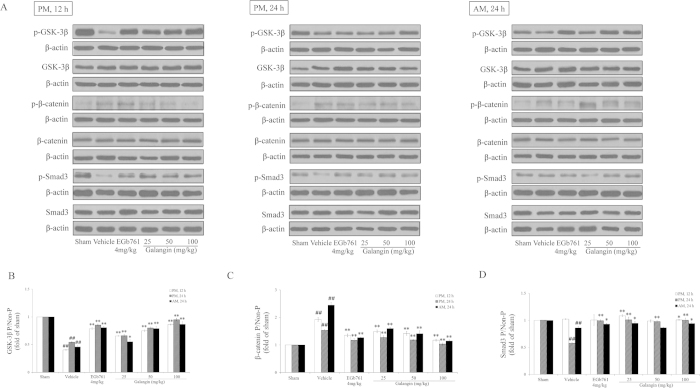
Effects of galangin on pGSK-3β, GSK-3β, pβ-catenin, β-catenin and pSmad3, Smad3 expression in rat brain after MCAO. (**A**) Representative western blots of pGSK-3β, GSK-3β, pβ-catenin, β-catenin and pSmad3, Smad3 and β-actin at three time points. (**B**) The ratio of pGSK-3β/GSK-3β, pβ-catenin/β-catenin, pSmad3/Smad3. The full-length blots are shown in Supplementary Materials. The values are expressed as the mean ± SD for the four independent experiments, and the data were analyzed by one-way ANOVA. ^##^*P* < 0.01, ^#^*P* < 0.05 versus the sham group; ^**^P < 0.01, ^*^P < 0.05 versus the vehicle control.

In response to the activation of the Wnt pathway, the ratio of pGSK-3β/GSK-3β in the vehicle-treated group was reduced significantly (P < 0.01). The ratio of pSmad3/Smad3 decreased significantly (P < 0.01) at PM 24 h and AM 24 h. The ratio of pβ-catenin/β-catenin was improved (*P* < 0.01) in the vehicle-treated group. Galangin (25 mg·kg^−1^, 50 mg·kg^−1^ and 100 mg·kg^−1^) and EGb761 improved the ratio of pGSK-3β/GSK-3β and decreased the ratio of pβ-catenin/β-catenin (*P* < 0.01 or *P* < 0.05). Galangin (25 mg·kg^−1^, 50 mg·kg^−1^ and 100 mg·kg^−1^) and EGb761 improved the ratio of pSmad3/Smad3 (*P* < 0.01) at PM 24 h. Galangin (25 mg·kg^−1^ and 100 mg·kg^−1^) and EGb761 also improved the ratio of pSmad3/Smad3 (*P* < 0.05) at AM 24 h. However, galangin at the dose of 50 mg·kg^−1^ did not improve the ratio of pSmad3/Smad3 at AM 24 h (Fig. 7).

## Discussion

Pathophysiologic responses in the brain after stroke are highly complex. In recent years, the concept of the NVU has emerged as a new paradigm for understanding the pathology of central nervous system disease, including stroke^3^,^24^,^25^,^26^,^27^. Within the NVU conceptual framework, brain function and dysfunction manifest at the level of cell-cell signaling between neuronal, glial and vascular elements. During the early phase of neurovascular injury, BBB perturbations predominate, with key roles for various matrix proteases. Understanding how neurovascular signals and substrates make the transition from initial injury to angiogenic recovery will be important if we are to find new therapeutic approaches to stroke^5^. In addition to brain injury responses, regenerative responses are also activated by stroke, such as vascular remodeling, angiogenesis and neurogenesis.

The results of neurological scores and cerebral infarct volume revealed that galangin has an excellent effect against focal cerebral ischemia. The perturbation of BBB function is one of the most important factors of early neurovascular damage. BBB disruption eventually exacerbates long-term disability. Our results indicate that galangin at doses of 50 mg·kg^−1^ and 100 mg·kg^−1^ could attenuate cerebral edema, reducing the permeability of EB at PM 12 h, PM 24 h and AM 24 h after MCAO. The analysis of changes to NVU structure and BBB permeability revealed that galangin protected the structure of NVUs and BBB permeability. Considering the outstanding protection of galangin on the structure of NVUs and integrity of the BBB, we further explored the mechanisms of this effect using RT-PCR and western blot.

The Wnt pathway plays important roles in multiple physiological and pathological processes^28^. GSK-3β and β-catenin are key members of the Wnt signaling pathway. GSK-3β is involved in modulating a variety of functions, including cell signaling, growth metabolism, and various transcription factors that determine the survival or death of the organism^29^. Wnt/β-catenin signaling might have multimodal effects for the prevention of the destructive effects of stroke. In the absence of Wnt ligands, the transcription factor β-catenin is phosphorylated by a protein complex containing GSK-3β^30^. The phosphorylated β-catenin is constantly degraded to prevent its accumulation and translocation to nucleus^30^,^31^. HIF-1α is a master regulator of cellular adaptation to hypoxia and has been suggested as a potent therapeutic target in cerebral ischemia. When cellular oxygen supply is reduced after MCAO, HIF-1α is activated, and its downstream protein VEGF is secreted, a critical factor for angiogenesis is produced and secreted from a variety of cell types to increase capillary permeability and stimulate the proliferation of endothelial cells^32^,^33^,^34^. However, whether VEGF is secreted in a HIF-1α-independent or -dependent way remains uncertain in this MCAO model. Upon binding of certain Wnt ligands, the Frizzled-1 receptor dimerizes with LRP6, forming a co-receptor complex, whose signaling inhibits the phosphorylation of β-catenin. Subsequently, β-catenin is stabilized, leading to its accumulation^35^,^36^. β-catenin then translocates itself to the nucleus and regulates the expression of target genes to promote angiogenesis and neurogenesis^37^,^38^,^39^,^40^. Smad3 is required for the interaction with β-catenin and protects β-catenin from ubiquitin-proteasome-dependent degradation. Smad3 also facilitates the translocation of β-catenin^41^.

In our study, RT-PCR and western blot revealed that HIF-1α was up-regulated by MCAO. Galangin down-regulated HIF-1α, VEGF, Frizzled-1 and LRP6, reduced the phosphorylation of β-catenin, and increased the phosphorylation of Smad3. This suggested that galangin could activate VEGF in a HIF-1α-dependent way and then activate the Wnt pathway after acute ischemic stroke. Moreover, the translocation of β-catenin caused by galangin could further activate Smad3. Smad3 could in turn protect β-catenin from degradation^41^. C-terminal Smad3 interacts with both the N-terminal region and the middle region of β-catenin in a TGFβ-dependent manner^41^. Smad3 is closely related to the TGFβ signal pathway^42^,^43^,^44^,^45^,^46^, which suggests that the activation of Smad3 could regulate the growth and differentiation of cells. Based on the above mechanisms, galangin might mediate neurogenesis and angiogenesis after MCAO.

*In vivo*, the BBB together with its neuronal and non-neuronal surrounding represents the NVU, which is characterized by a functional interaction between brain endothelial cells, astrocytes, pericytes, microglia and neurons. Brain endothelial cells effectively separate the brain from cerebral blood flow through a complex and precisely regulated system of tight-junction (TJ) proteins^47^. The activation of HIF-1α after MCAO mediated signaling disrupts TJ resulting in increased BBB permeability^48^. The up-regulated VEGF could further increase vascular leakage^49^. Increased BBB permeability correlated with disruption of TJ protein organization has emerged as a key pathological hallmark in neurodegenerative diseases. AQP-4, the predominant water channel in the brain, is enriched in astrocytes at BBB and brain-cerebrospinal fluid interfaces, indicating regulation of brain water homeostasis between fluid compartments and brain parenchyma^50^. Loosening of TJs occurs as endothelial cells disengage in preparation to move; and up-regulation of extracellular proteases such as MMPs is required for angiogenesis. Accumulated evidence suggests that MMP-9 activation is closely related to BBB disruption after stroke. In our study, down-regulation of occludin and up-regulation of AQP-4 and MMP-9 were found in MCAO rats. Ultrastructural observation showed serious leakage of lanthanum ions from the lumen of vessels and the astrocytes were edema. Galangin improved the mRNA expression of occludins, such as claudin-5 and ZO-1. It also reduced the expression of AQP-4 and MMP-9. However, its effect on TJs still requires further investigation.

Considerable evidence now suggests that the Wnt/β-catenin pathway is involved in neurogenesis^51^,^52^,^53^. Given that MCAO activated the Wnt and HIF-1α/VEGF pathways, vascular remodeling, angiogenesis and neurogenesis might be promoted in rats after MCAO. Factor VIII, GFAP and MAP-2 were up-regulated by galangin. These findings might suggest that galangin promotes the regeneration of vascular endothelial cells, astrocytes and neurons.

In conclusion, this study provided comprehensive evidence supporting the potential effects of galangin on NVU remodeling and its underlying protective mechanisms. Galangin could ameliorate neurological defects and NVU damage after MCAO. Moreover, galangin could up-regulate Wnt/β-catenin and HIF-1α/VEGF signaling to protect the rats injuried by MCAO. VEGF was activated along with HIF-1α. Among nodes, VEGF and β-catenin were the key nodes of these two coupled pathways. All of the data in this study demonstrates that the anti-cerebral effect of galangin might result from the improvement of the microenviroment in NVUs. Therefore, galangin might function as a multi-target drug in the treatment of ischemic stroke.

## Methods

### Experimental procedures

#### Animals

Adult male Sprague-Dawley rats weighing 250–270 g were obtained from the Animal Breeding Center of Beijing Vital River Laboratories Company (Beijing, China). All animals were housed individually at 22 ± 2 °C with a relative humidity of 50 ± 10% and a 12-h light/12-h dark cycle. The animals had free access to food and water. The experimental procedures were approved by the China Academy of Chinese Medical Science’s Administrative Panel on Laboratory Animal Care. All animal experiments were performed in accordance with institutional guidelines and ethics.

### Chemical and Reagents

Galangin (purity 98.0%) was purchased from Nanjing Zelang Medical Technology Co. Ltd (Nanjing, China). The positive control, EGb761, was purchased from Dr. Willmar Schwabe (Karlsruhe, Germany). Antibodies for MAP-2, VEGF, AQP-4, GFAP, MMP9, GSK-3β, p-GSK-3β, Smad3, pSmad3, β-catenin, pβ-catenin were purchased from Abcam (CA, USA). R-PE-conjugated goat anti-rabbit IgG (H + L) was purchased from Cell Signaling Technology (MA, USA). Evans blue was purchased from Sigma Chemical Co. Ltd (St. Louis, MO, USA). RNA extraction kit, HiFi-MMLV cDNA first strand synthesis kit, Ultra SYBR Mixture, DNase 1, RNA Loading Buffer were all purchased from CWbio. Co. Ltd (Beijing, China).

### Animal Models and Experimental Protocol

After 48 h of acclimatization, the rats were anesthetized with chloral hydrate at a dose of 400 mg·kg^−1^(i.p.). The rectal temperature was recorded and maintained at 37 ± 0.5 °C throughout the surgical procedure. The MCAO operation by the intraluminal filament method was performed according to a previous method with some modifications^54^. Briefly, 4–0 monofilament nylon suture with a round tip was inserted from the left external carotid artery into the lumen of the internal carotid artery to occlude the origin of the MCA. The rats were sacrificed 12 h or 24 h after the MCAO.

The rats were randomly divided into the following 6 groups (n = 10/group): sham group; vehicle control; positive control EGb761 group (4 mg·kg^−1^); and galangin-treated groups (25, 50 and 100 mg·kg^−1^). Galangin was dissolved in sterile saline (containing 5% Tween 80) to make the stock solution. Dilutions were then prepared for the administration of the different doses. Galangin and EGb761 were both administered by intragastric administration (i.g.) 15 min prior to a 12-h MCAO (PM 12 h), 15 min prior to a 24-h MCAO (PM 24 h) and 6 h after 24-h MCAO (AM 24 h). The sham and vehicle-treated rats were injected with physiological saline. Neurological defects and BBB permeability were determined at 12 h and 24 h after the MCAO followed by the isolation of the related proteins, ultrastructural changes to the BBB and western blot of BBB-related proteins. For RT-PCR, galangin or EGb761 was administered by intragastric administration (i.g.) 15 min prior to 6-h MCAO 6 h (PM, 6 h), 15 min prior to 12-h MCAO (PM, 12 h), and 6 h after 12-h MCAO (AM, 12 h). The genes of interest were detected 6 h and 12 h after the MCAO.

### Blinding and Randomization

Treatment groups were allocated in a randomized fashion. Researchers were blind to the allocation of treatment during surgeries and outcome evaluations.

### Evaluation of neurological deficits

To reveal the effect of galangin on the neurological defects caused by the MCAO operation, the neurological defects were determined by a single researcher at 12 h and 24 h after MCAO. The researcher was blind to the experimental treatment groups. The neurological behaviors were scored on 5-point scale as described previously^55^.

### Evaluation of cerebral infarct volume

The cerebral infract volumes measured with TTC staining were used for describing the severity of the cerebral ischemia. After 24 h of ischemia, the brains were quickly removed and sliced into 6 coronal sections 2 mm thick. The brain slices were treated with 2% TTC saline solution and incubated at 37.5 °C for 30 min, followed by 10% formalin fixation overnight according to a previously described method^56^. After staining with TTC, the normal tissue stained a rose-red color, and the infarct tissue was white. The stained slices were photographed and recorded. The adjusted infarct areas and both the hemisphere areas of each slice were determined by an image analysis system (Image-Pro Plus 6.0). Infarct volume was determined by integrating the cross-sectional area of infarction at each stereotaxic level and the thickness of each slice according to the following formula: *V* = *t* × (A1 + A2 + …An), where V is the infarct volume (mm^3^), t is the thickness of the slice and A is the infarct area (mm^2^). Correction for edema of the infarct area was performed as described by Lin *et al.*^57^.

### Evaluation of cerebral edema

Following decapitation at 12 h or 24 h after MCAO, the left and right cerebral hemispheres were obtained and immediately weighed to obtain the wet weight. The rate of cerebral edema was calculated according to the following formula:

Rate of cerebral edema = [(left cerebral hemisphere weight–right cerebral hemisphere weight)/whole cerebral weight] × 100%

### Evaluation of BBB permeability

BBB permeability was assessed by the concentration of EB in brain cortex. EB was dissolved in formamide, and then the solution was serially diluted to obtain a standard curve at a wavelength of 610 nm (Fig. S1). The animals underwent MCAO followed by an intravenous application of EB (2% solution, 2 ml·kg^−1^) and perfusion of the heart with 0.9% NaCl (1,000 ml·kg^−1^). The brain was divided into the left cerebral hemisphere and right cerebral hemisphere. After weighing, the whole brain was immersed in 1 ml formamide at 60 °C for 24 h. Then, the mixture was centrifuged at 10,000 r·min^−1^ for 15 min, and the supernatant was obtained for further detection. Then 100 μl of supernatant was aliquoted into the microplate, and the optical density (OD) value was measured at the wavelength of 610 nm. The amount of EB (μg) in the brain (g) was calculated from the OD value of extracted brain using the formula from the standard curve. The detailed calculation was as follows:

EB per gram brain tissue (μg·g^−1^) = [EB concentration (μg·ml^−1^) × 1 ml (Formamide volume)]/ Brain weight (g).

### Changes to NVU structure and BBB permeability

The tracer lanthanum was used to assess the restoration of the BBB function by previously reported methods with modification^58^,^59^. After anesthesia, the heart was exposed and the left ventricle was perfused with 0.9% saline, followed by perfusion for 2 h with fixative consisting of 4% lanthanum nitrate and 6% glutaraldehyde in 0.1 M sodium cacodylate (pH 7.40–7.50). At the end of perfusion, the temporal cerebral cortices were isolated and cut into 1 to 2 mm^3^ pieces. The samples were kept in the same fixative without lanthanum nitrate for 1 h, rinsed in washing solution (0.15 M NaCl plus 0.2 M sucrose), post-fixed in 1% OsO_4_ diluted in the same solution, dehydrated in a graded acetone series and embedded in Epon 812. For light-microscopic examination, semithin sections (1 μm thick) were cut on an ultramicrotome (Reichert S Ultra-Cut, Leica) and stained with 1% toluidine blue (TB). For TEM, ultrathin sections (60 nm thick) were obtained of selected blocs of cerebral regions after histological examination of TB sections. The ultrathin sections were mounted on copper grids (200 mesh) and double-contrasted with uranyl acetate and led citrate for examination in a LEO 906 TEM (Zeiss, Oberkochen, Germany) operated at 60 kV. In the analysis of the TEM images, the number of neurons in the brain was also counted.

### RT-PCR

Total RNA was extracted from rat brain using the RNA extraction kit. The primers used for PCR are shown in Table 1. The specificity of each target amplicon was assessed by dissociation curve analysis, and all amplicons spanned exon-exon regions to avoid genomic amplification. RT-PCR was performed on an ABI7500 Sequence Detection System (Applied Biosystems, CA, USA) in 96-well plates using a final volume of 20 μl and the following cycle conditions: 95 °C for 10 min, and then 45 cycles of 15 s at 95 °C and 1 min at 60 °C. All RT-PCR mixtures contained 2 μl of cDNA template, 10 μl of UltraSYBR Mixture (With ROX) and 10 μM of each target-specific primer. The results were analyzed using the Applied Biosystems software, and expression levels were calculated from a linear regression of the standard curve. Results of quantification are given according to the formula of 2^−△△ct^. GADPH was used as an internal standard. All real-time PCRs for each sample were performed in triplicate.

### Western blot of BBB-related proteins

The expression of MAP-2, GFAP, MMP-9, AQP-4, VEGF, pSmad3, Smad3, pGSK-3β, GSK-3β, pβ-catenin, β-catenin in cerebral cortex were determined by western blot. After anesthesia, rats were sacrificed by decapitation. The brains were quickly dissected, and the cerebral cortex was homogenized in ice-cold lysis buffer. The homogenate was incubated on ice for 30 min and then centrifuged at 12,000 rpm for 20 min at 4 °C. The supernatant was collected, and the protein concentrations contained in supernatant were measured by BCA assay. The protein samples with loading buffer added were boiled for 10 min before loading onto SDS-polyacrylamide gel. After electrophoresis, the gel was electroblotted onto PVDF membranes. Membranes were blocked in Tris-buffered saline (TBS) with 1% Tween-20 (TBST) and 5% non-fat dry milk, then incubated with primary antibody overnight at 4 °C. Then, the membranes were washed several times with TBST prior to incubation with horseradish peroxidase-conjugated secondary antibody for 45 min at room temperature. After subsequent washes in TBST, the protein bands were visualized using the ECL detection kit. The relative intensities of the bands were quantified by densitometric analysis. The densitometric plots of the results were normalized to the intensity of the actin band.

### Statistics

The data are expressed as the mean ± SD. The statistical significance of differences between groups was determined by one-way analysis of variance (ANOVA). A P value < 0.05 was considered statistically significant.

## Additional Information

**How to cite this article**: Wu, C. *et al.* Wnt/β-catenin coupled with HIF-1α/VEGF signaling pathways involved in galangin neurovascular unit protection from focal cerebral ischemia. *Sci. Rep.* **5**, 16151; doi: 10.1038/srep16151 (2015).

## Supplementary Material

Supplementary Dataset 1

Supplementary Dataset 2

Supplementary Dataset 3

Supplementary Information

Supplementary Dataset 4

## Figures and Tables

**Table 1:** Primers for the Quantitative RT-PCR.

Gene	Sequences (5′ to 3′)	PCR product (bp)
HIF-1α *for*	GATGAATCAAAAGCAGTGACGAAGG	106
HIF-1α *rev*	ATGCCTTAGCAGTGGTCATTTCTTG	
VEGF *for*	CCGTCCTGTGTGCCCCTAATG	101
VEGF *rev*	CGCATGATCTGCATAGTGACGTTG	
Claudin-5 *for*	GCTTGTGGCACTCTTTGTTACCTTG	193
Claudin-5 *rev*	AGACACCGGCACCGTTGGATC	
Ctnnb1 *for*	GGACTCTAGTGCAGCTTCTGGGTTC	191
Ctnnb1 *rev*	ACAGATGGCAGGCTCGGTAATG	
Lrp6 *for*	TGGATGGAACAGAACGGGAGGT	104
Lrp6 *rev*	GTCGGAGTCAGCCCAGAAGAGC	
Tjp1 *for*	AGCCAGTTCCGCCTCTGTCCA	189
Tjp1 *rev*	GGGGATAGAAGGTCTGAGGAGGGTC	
Fzd1 *for*	GCTCAATAACGTGGATGCTCTGC	154
Fzd1 *rev*	TTTCCAGTTTCTCTGTCTTGGT	
Factor VIII *for*	AGTGCCAAGGAGCCATTCGC	185
Factor VIII *rev*	ATCAGGCTTCCCGTGGAGTTTC	
GAPDH *for*	TGGAGTCTACTGGCGTCTT	138
GAPDH *rev*	TGTCATATTTCTCGTGGTTCA	
