# Supplementary material for: Wnt/β-catenin coupled with HIF-1α/VEGF signaling pathways involved in galangin neurovascular unit protection from focal cerebral ischemia
Source: Sci Rep. 2015 Nov 5;5:16151. doi: 10.1038/srep16151 (PMC4633613; doi:10.1038/srep16151)
Supplement: Supplementary Information [file srep16151-s4.doc]

**Wnt/β-catenin coupled with HIF-1α/VEGF signaling pathways involved in galangin neurovascular unit protection from focal cerebral ischemia**

*Chuanhong Wua,[[1]](#footnote-2),2, Jianxin Chen^a,3^, Chang Chen ^a,1^, Wei Wang^3^, Limei Wen^4^, Kuo Gao^3^, Xiuping Chen^2^, Sihuai Xiong^5^, Huihui Zhao^*,3^, Shaojing Li^*,1,3^*

^1^Institute of Chinese Materia Medica, China Academy of Chinese Medical Sciences, Beijing 100700, China.

^2^State Key Laboratory of Quality Research in Chinese Medicine, Institute of Chinese Medical Sciences, University of Macau, Macao, 999078, China.

^3^Beijing University of Chinese Medicine, Beijing 100029, China.

^4^The first Affiliated Hospital of Xinjiang Medical University, Xinjiang, 830054, China.

^5^Beijing No.166 High School, Beijing 100006, China

MAP-2:

PM, 12 h


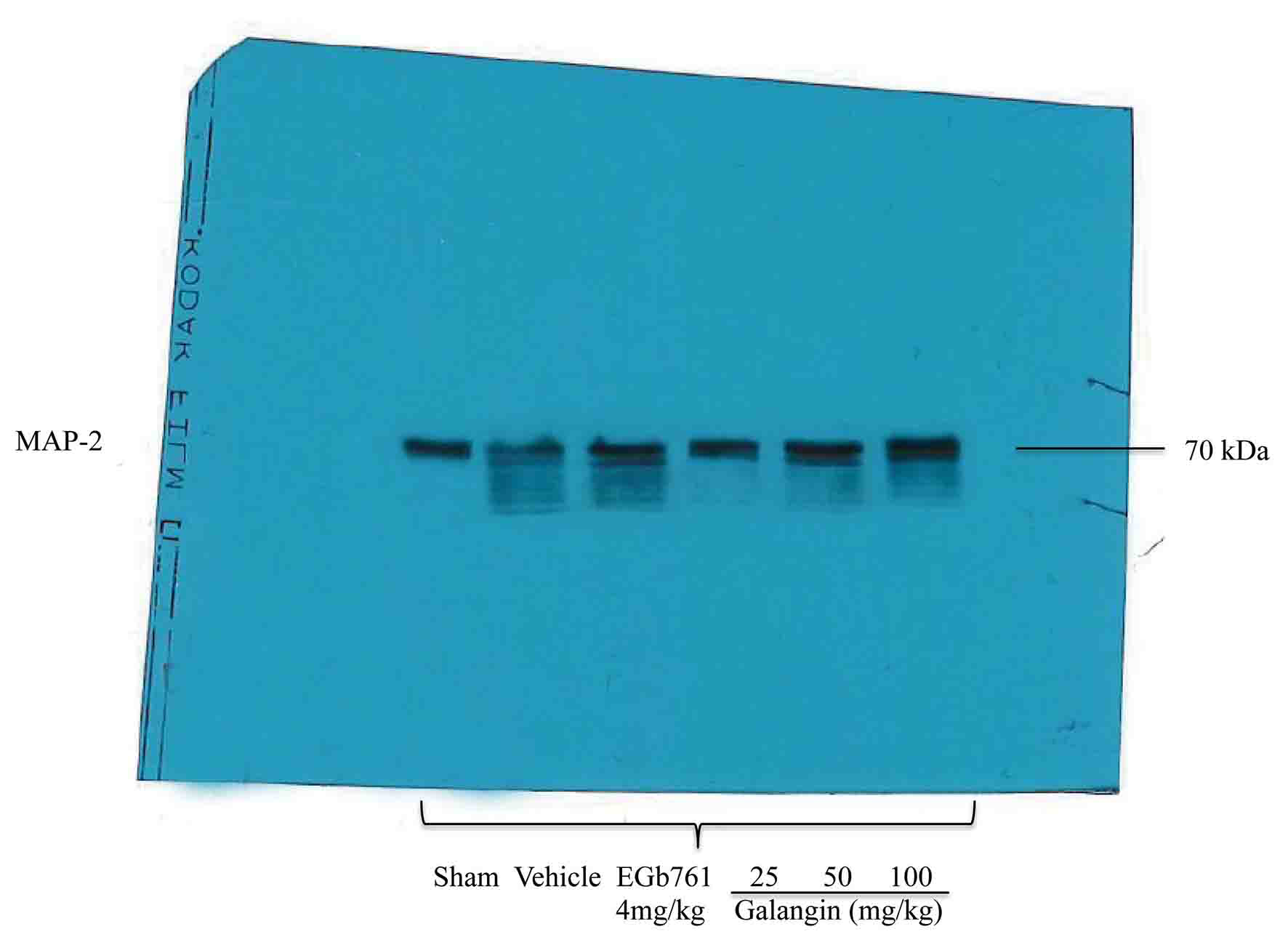


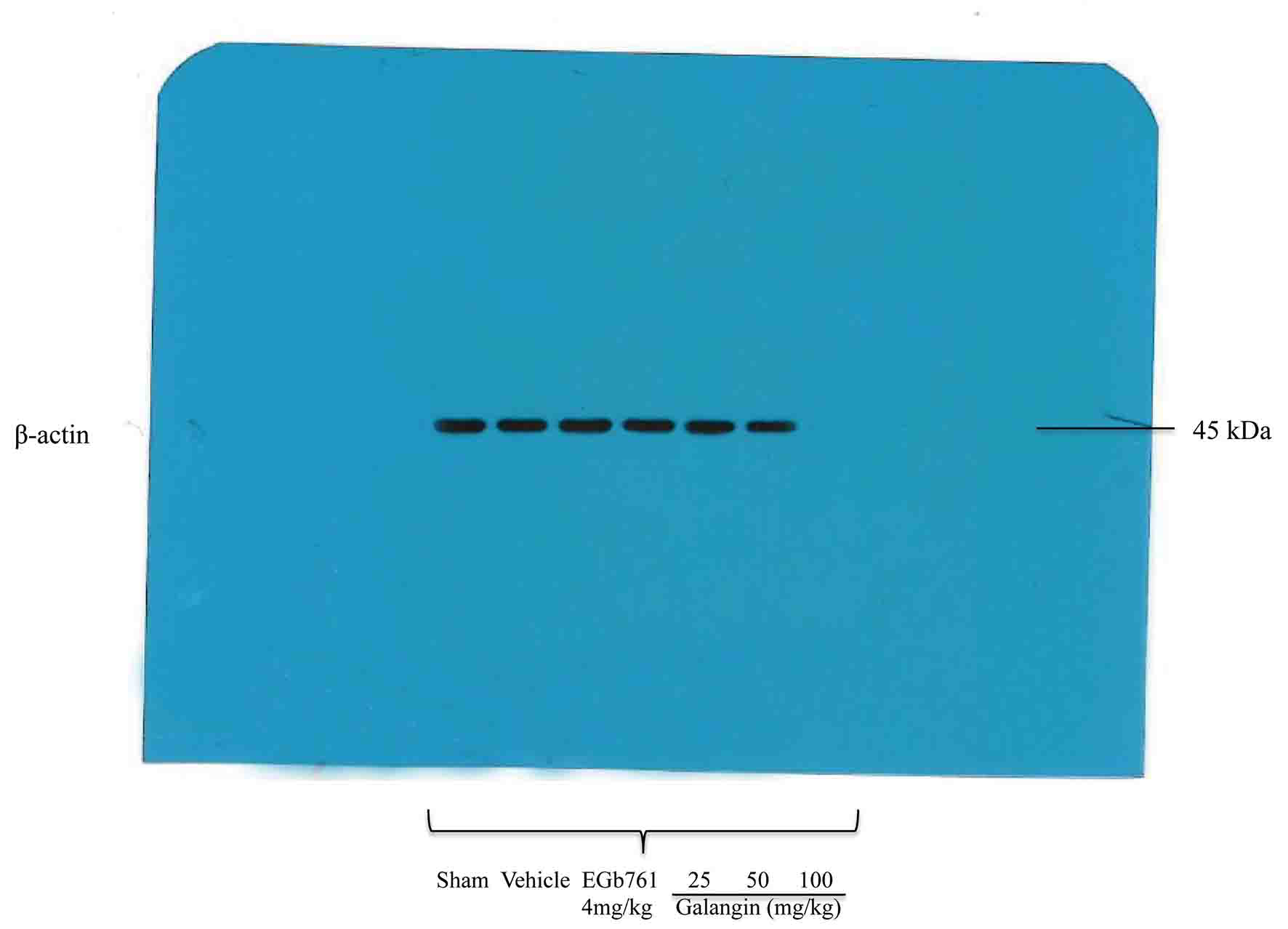


PM, 24 h


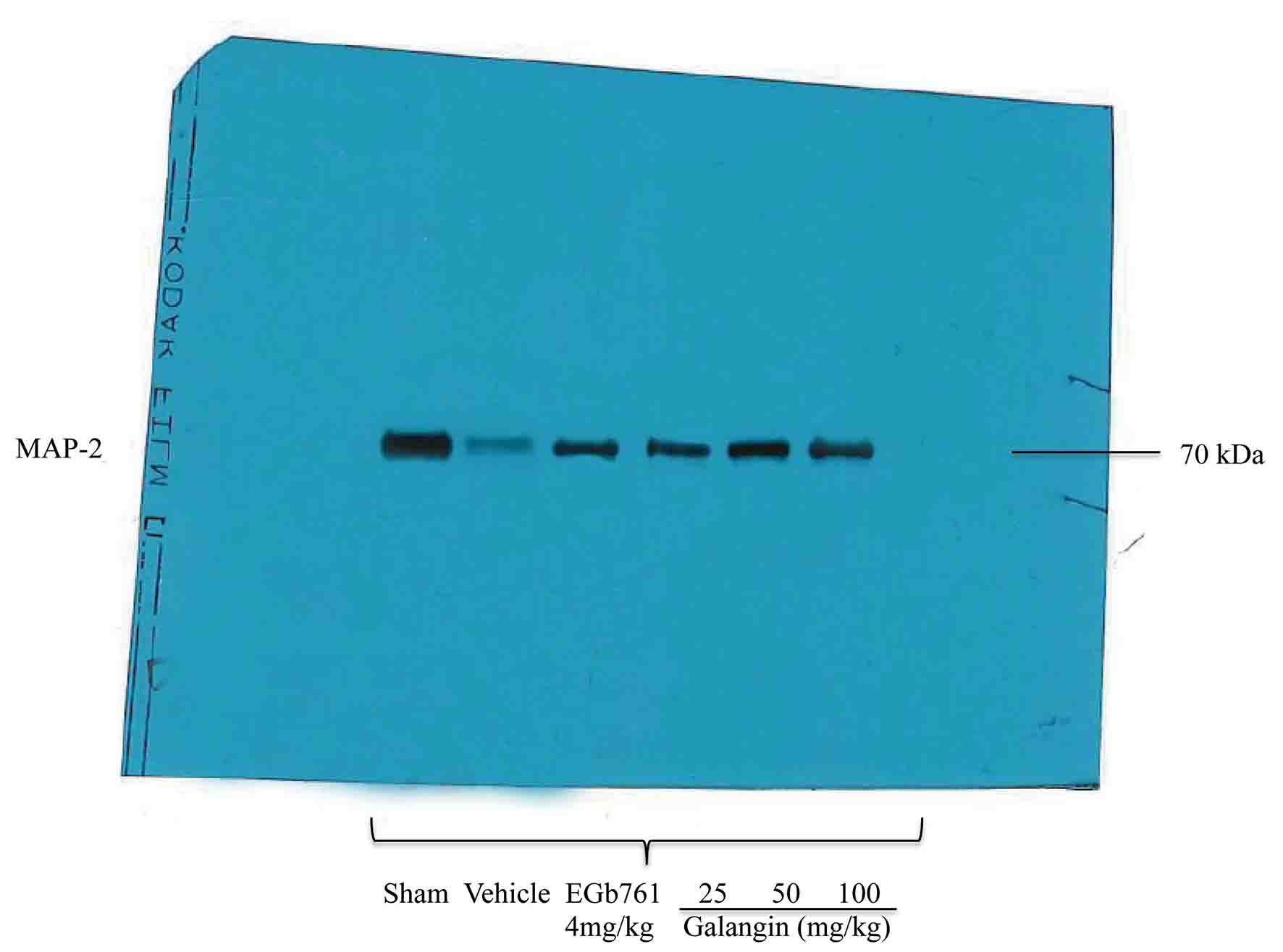


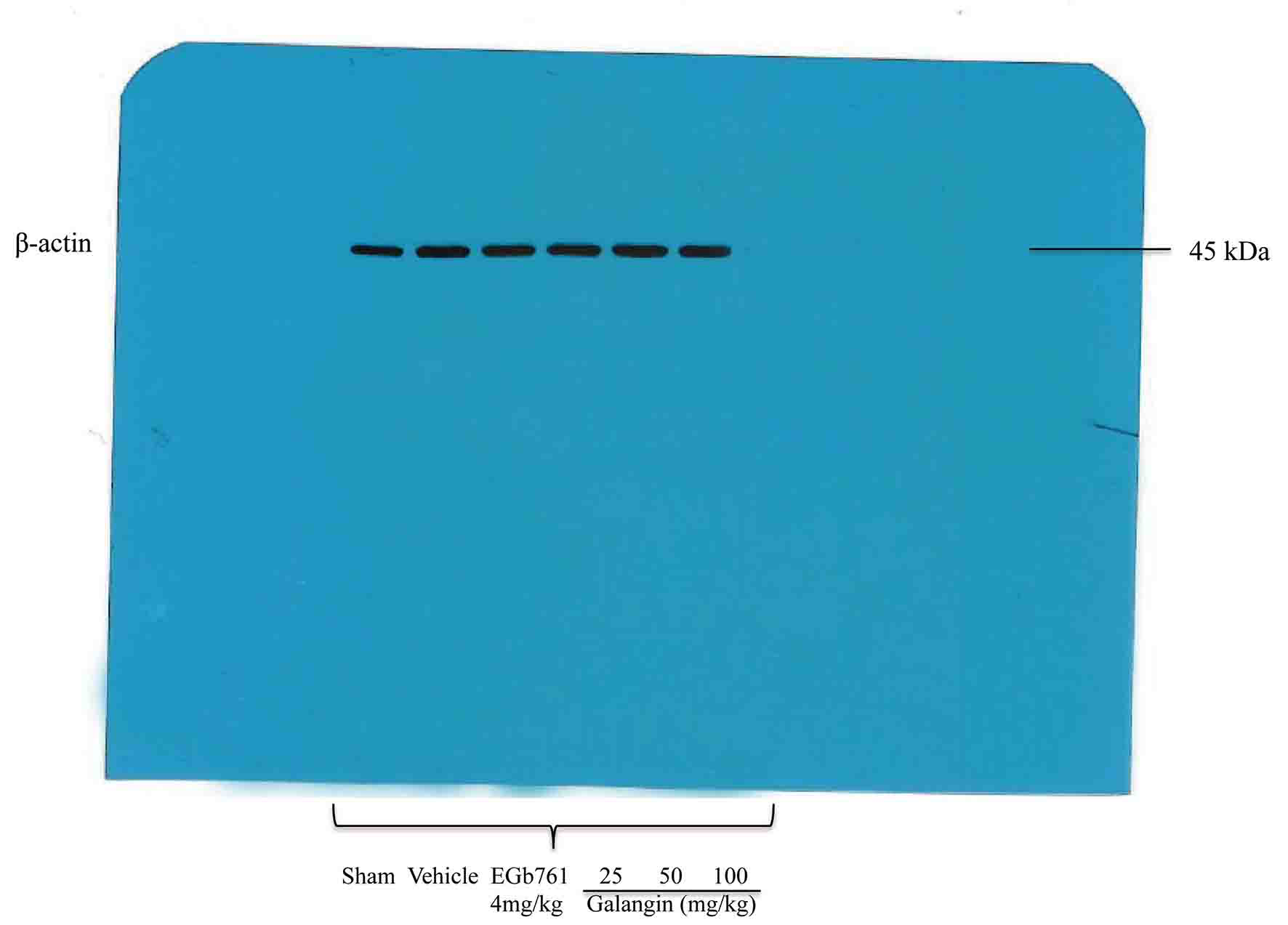


AM, 24 h


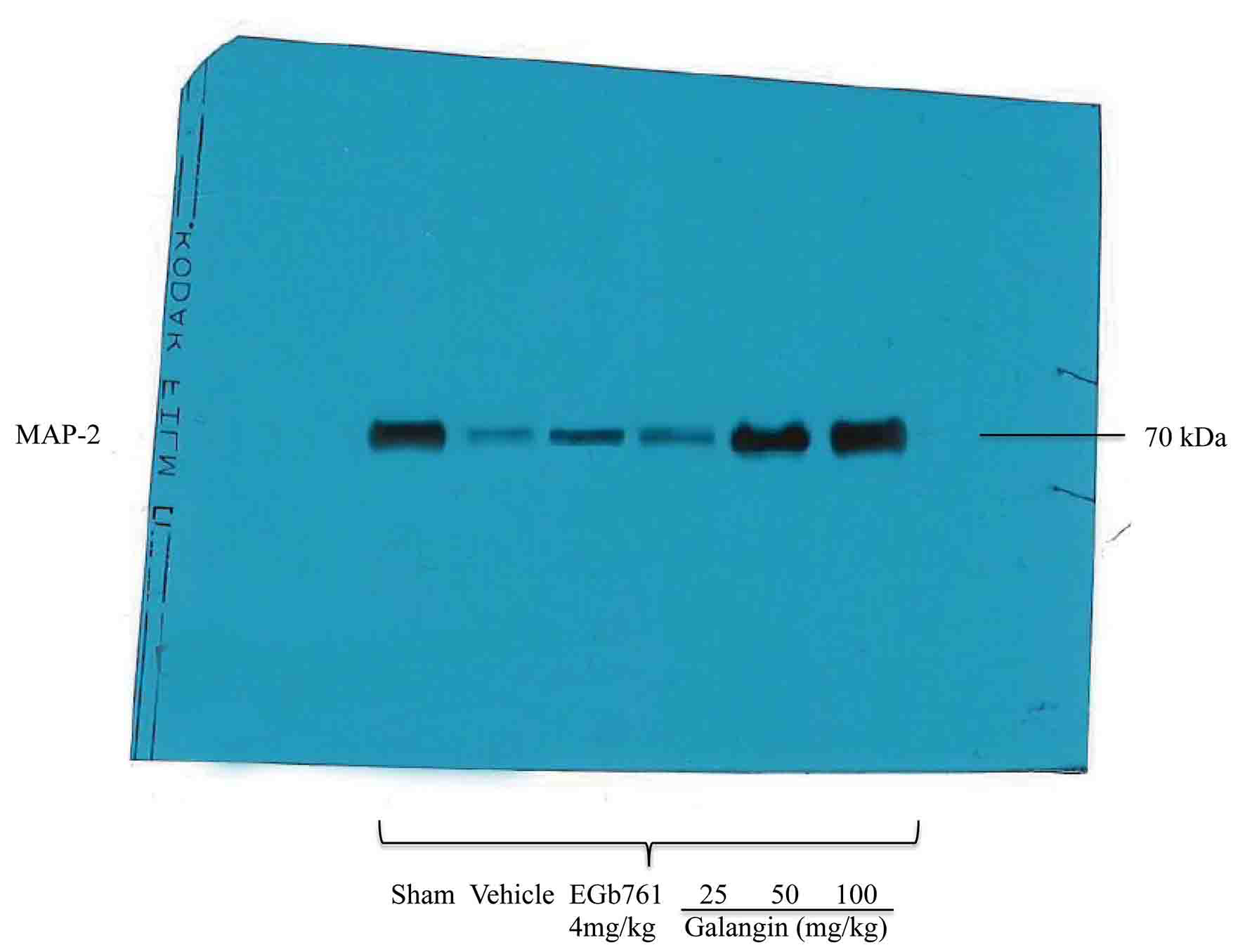


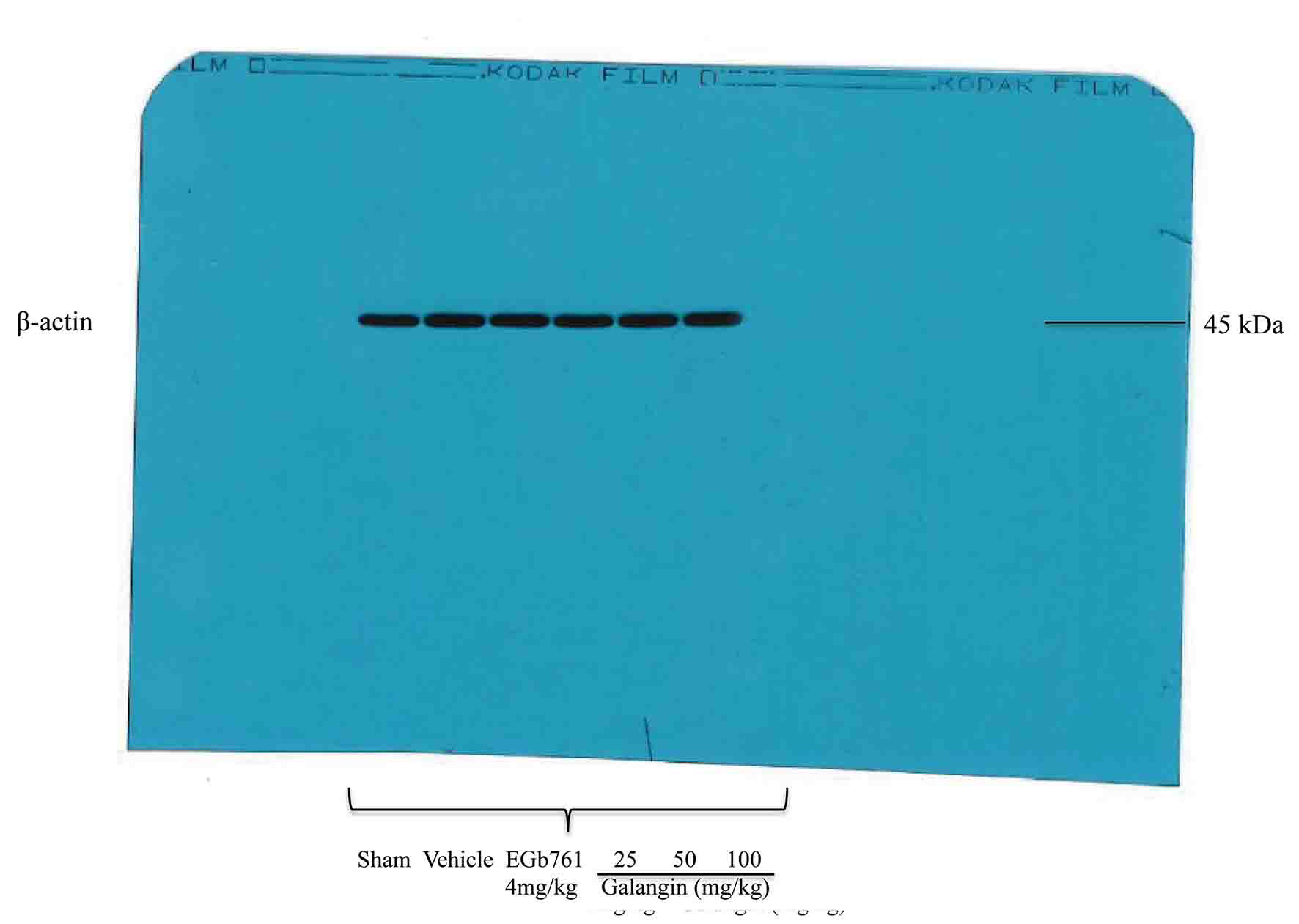


GFAP:

PM, 12 h


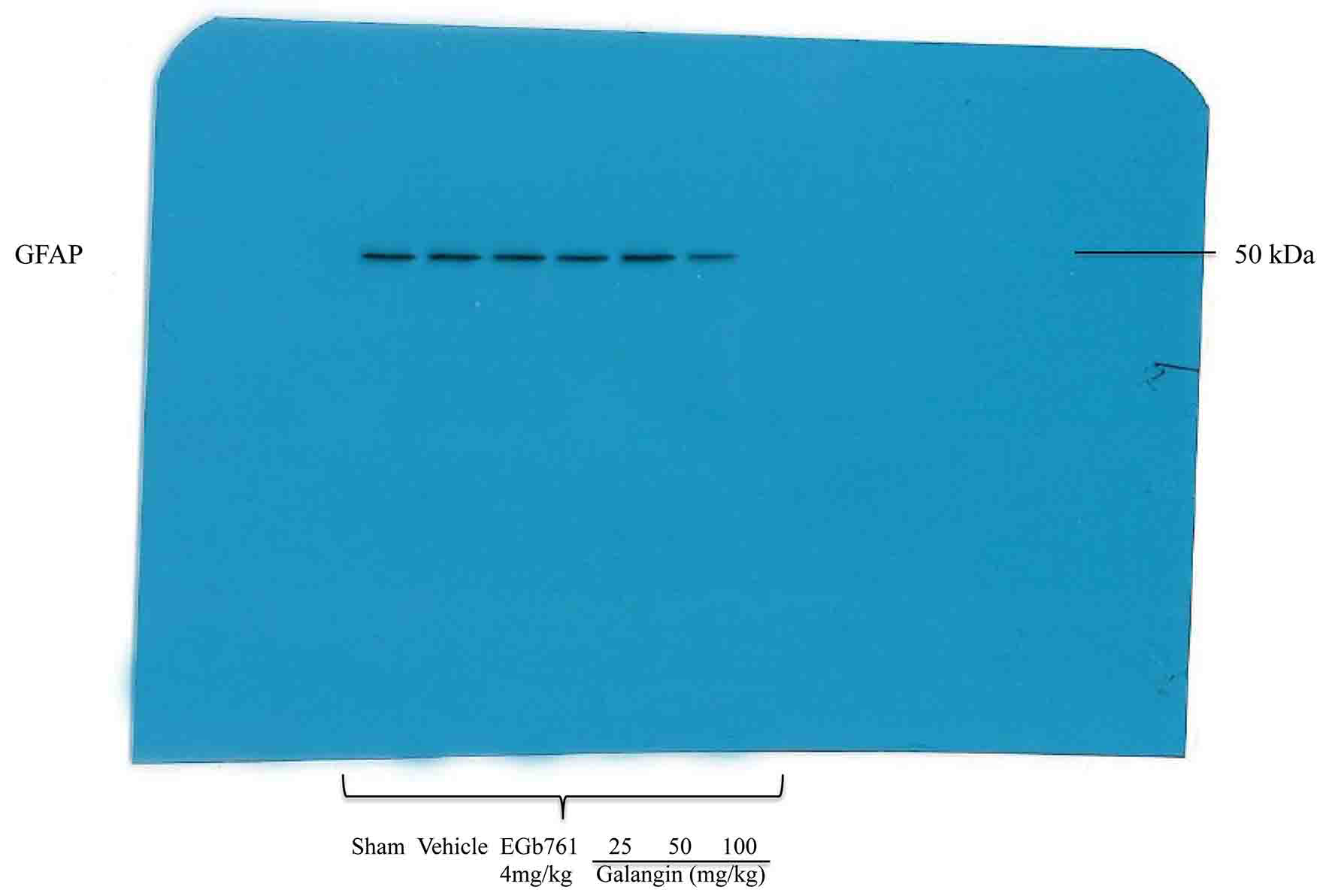


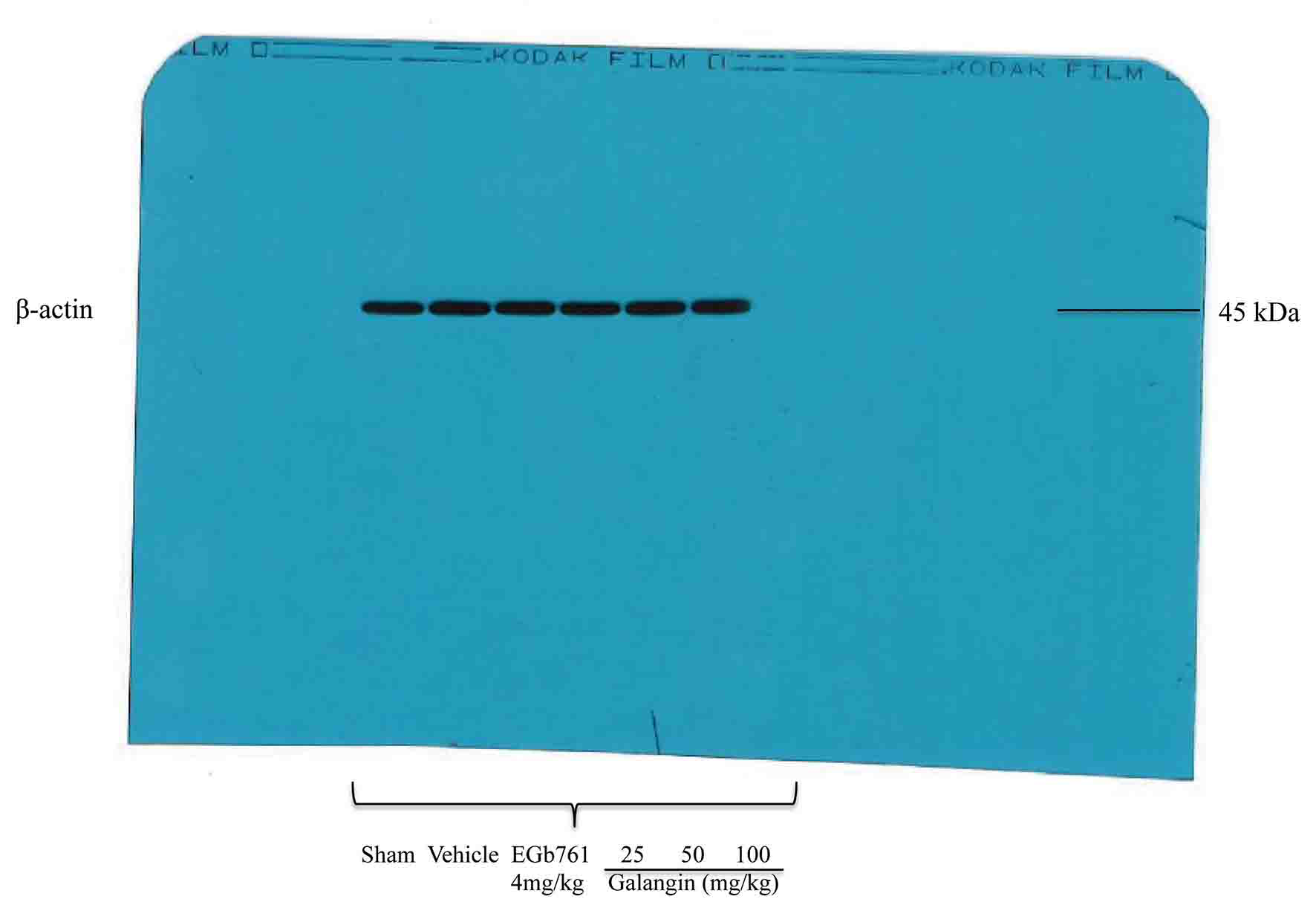


PM, 24 h


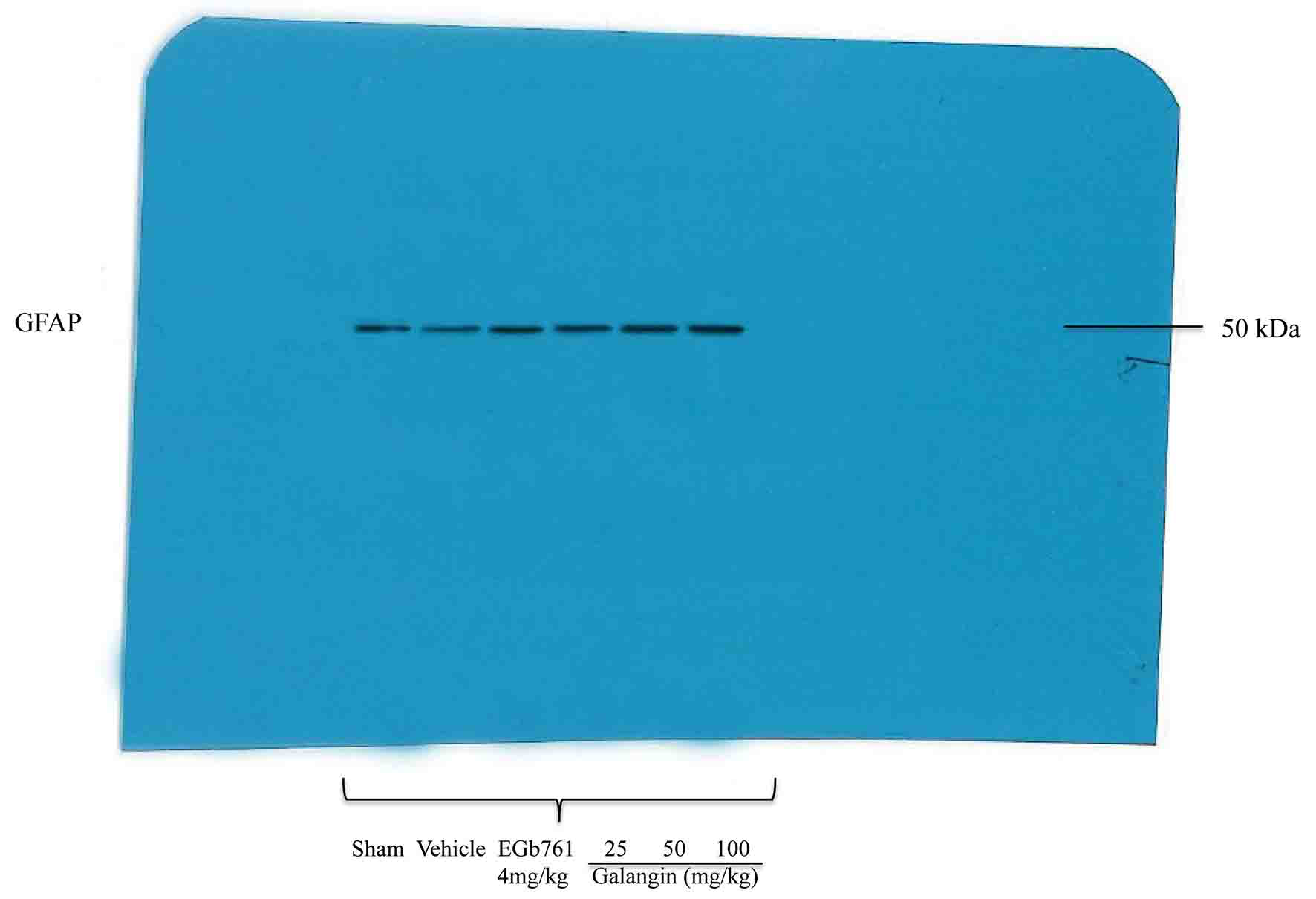


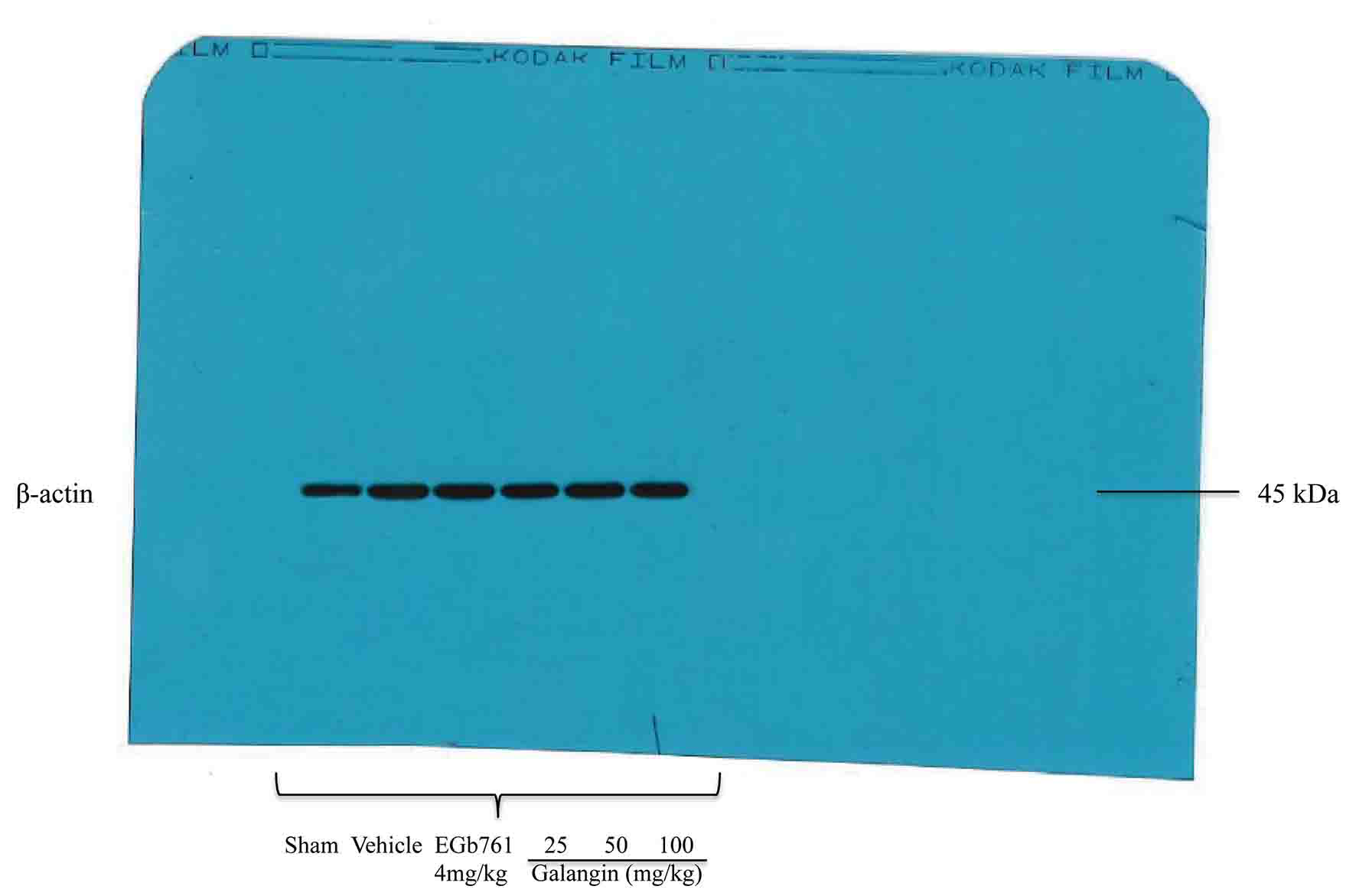


AM, 24 h


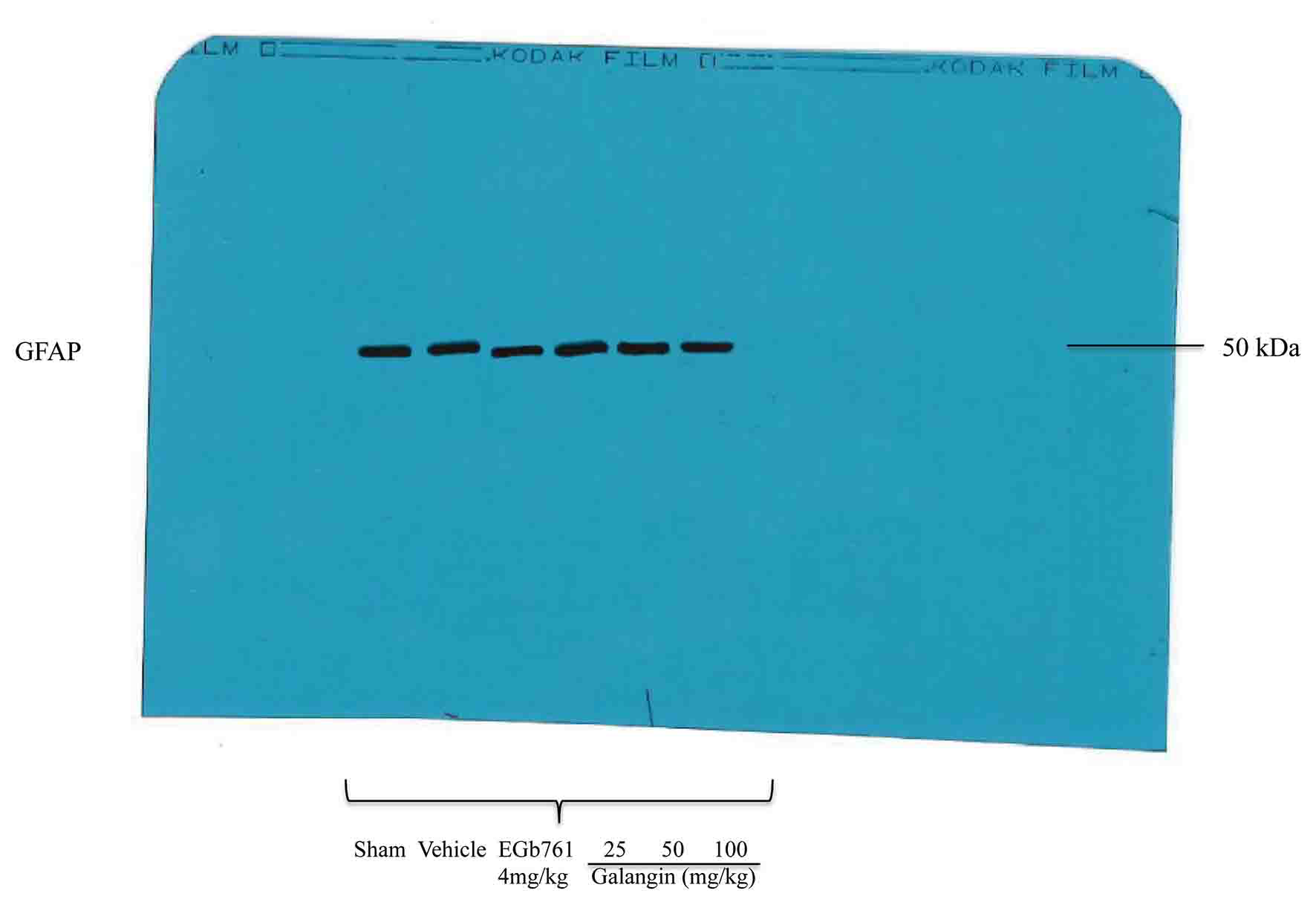


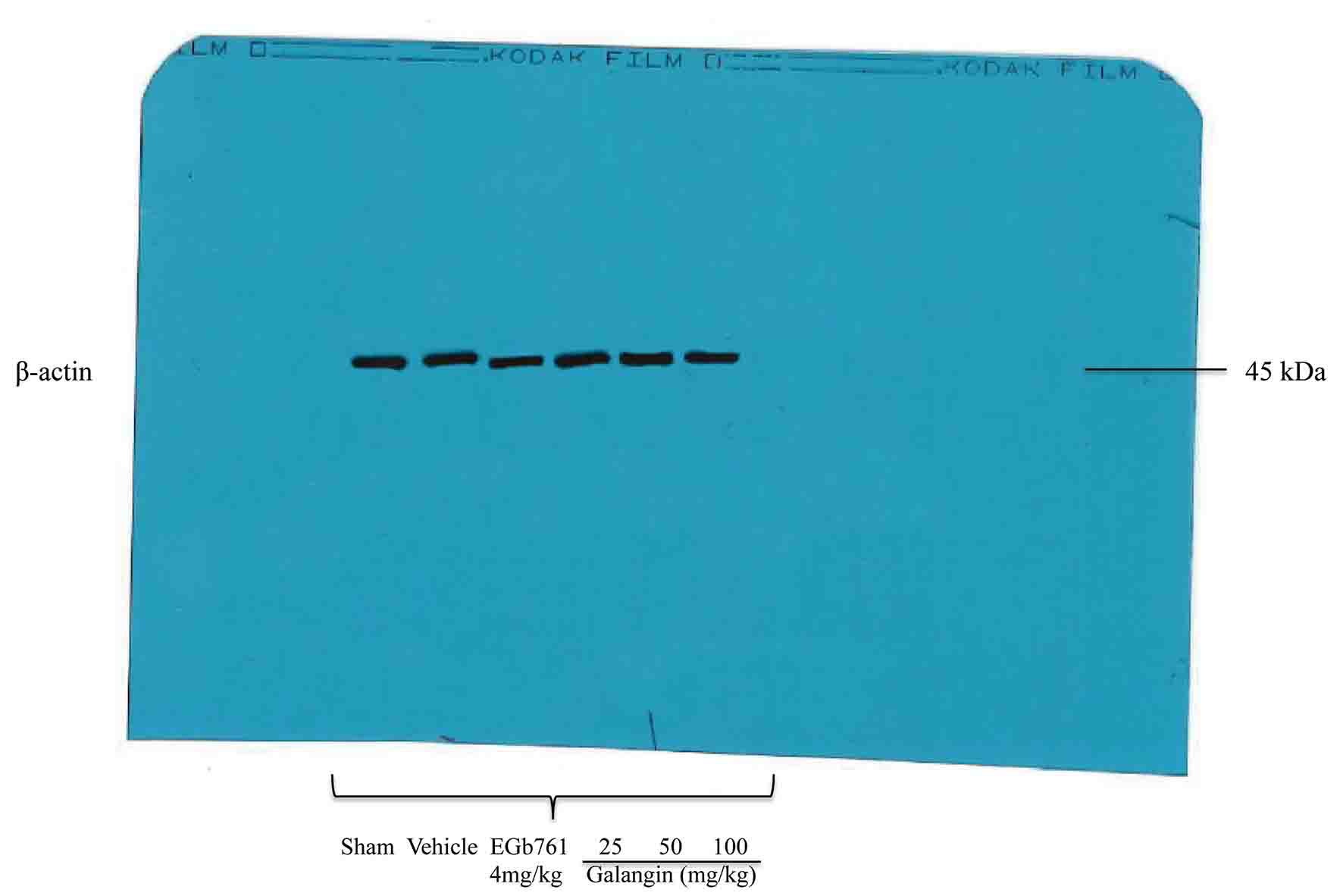


VEGF:

PM, 12 h


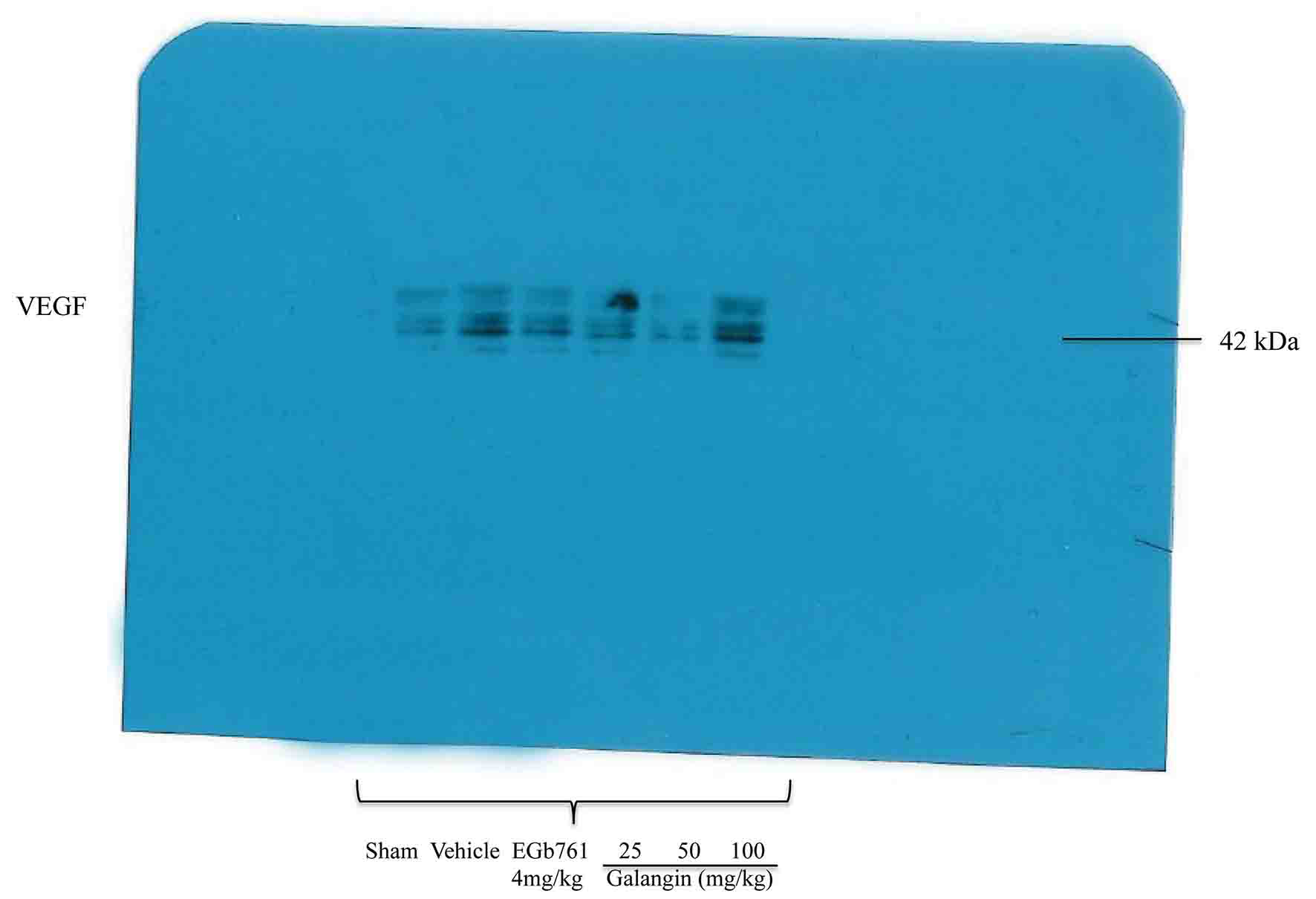


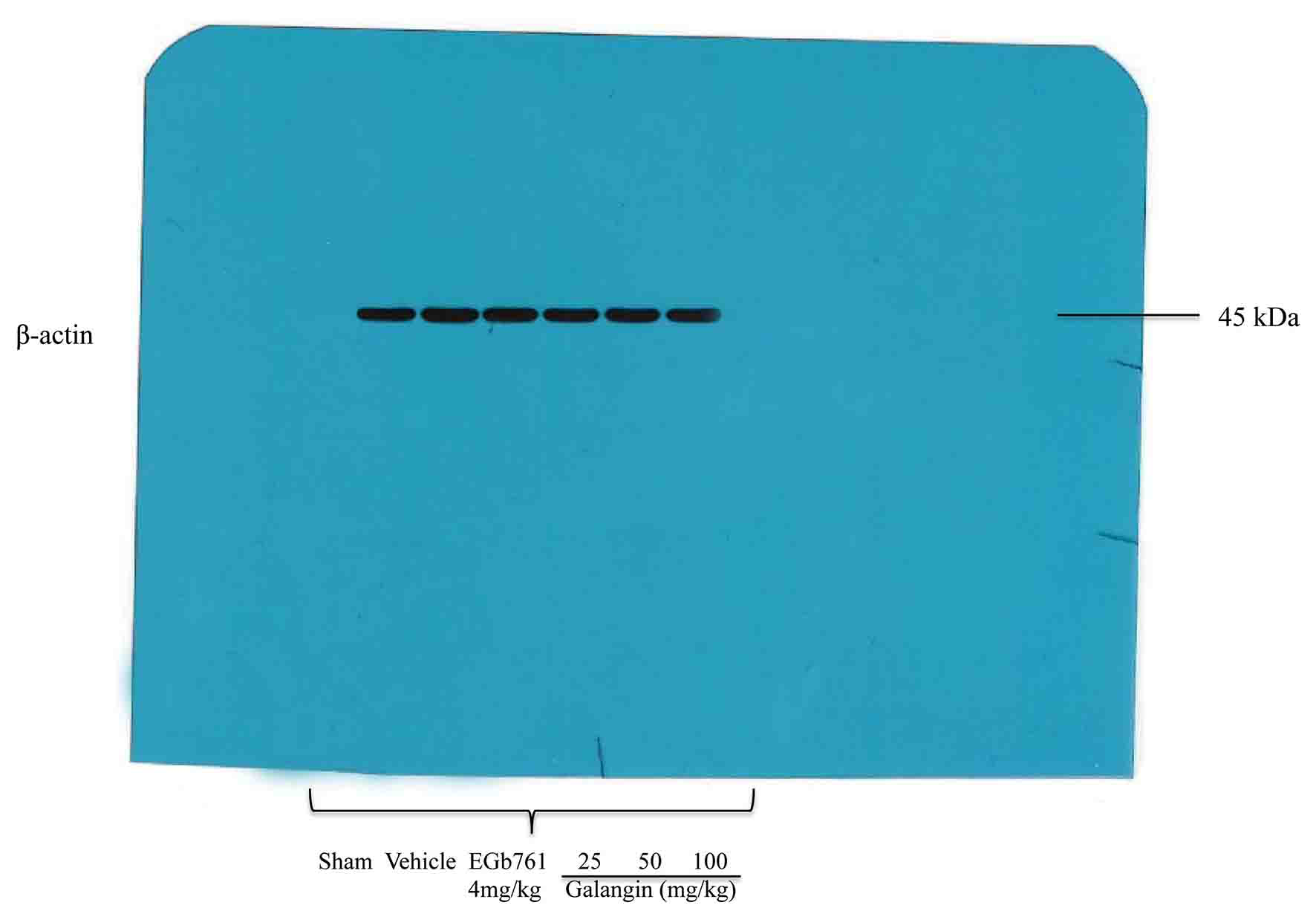


PM, 24 h


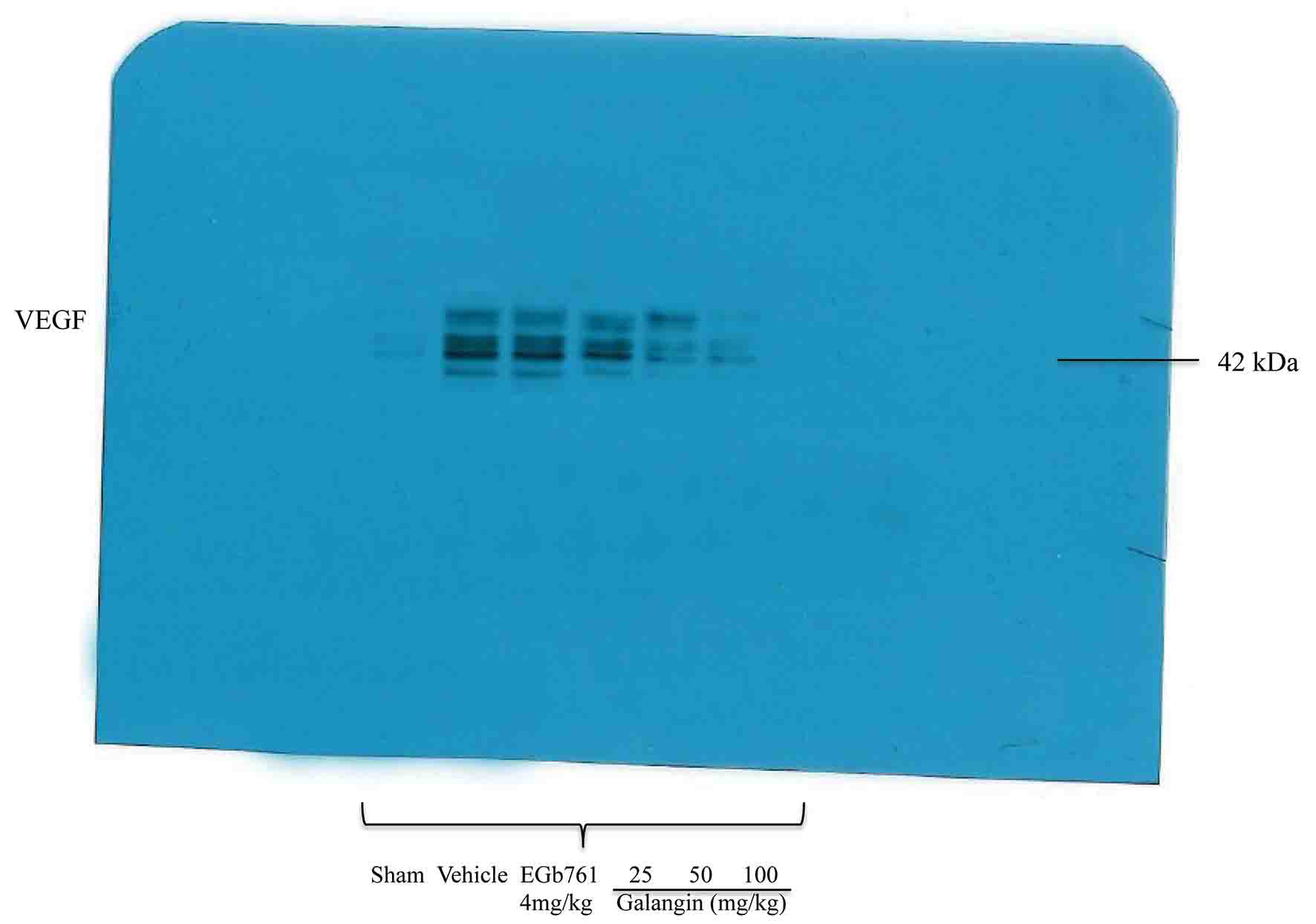


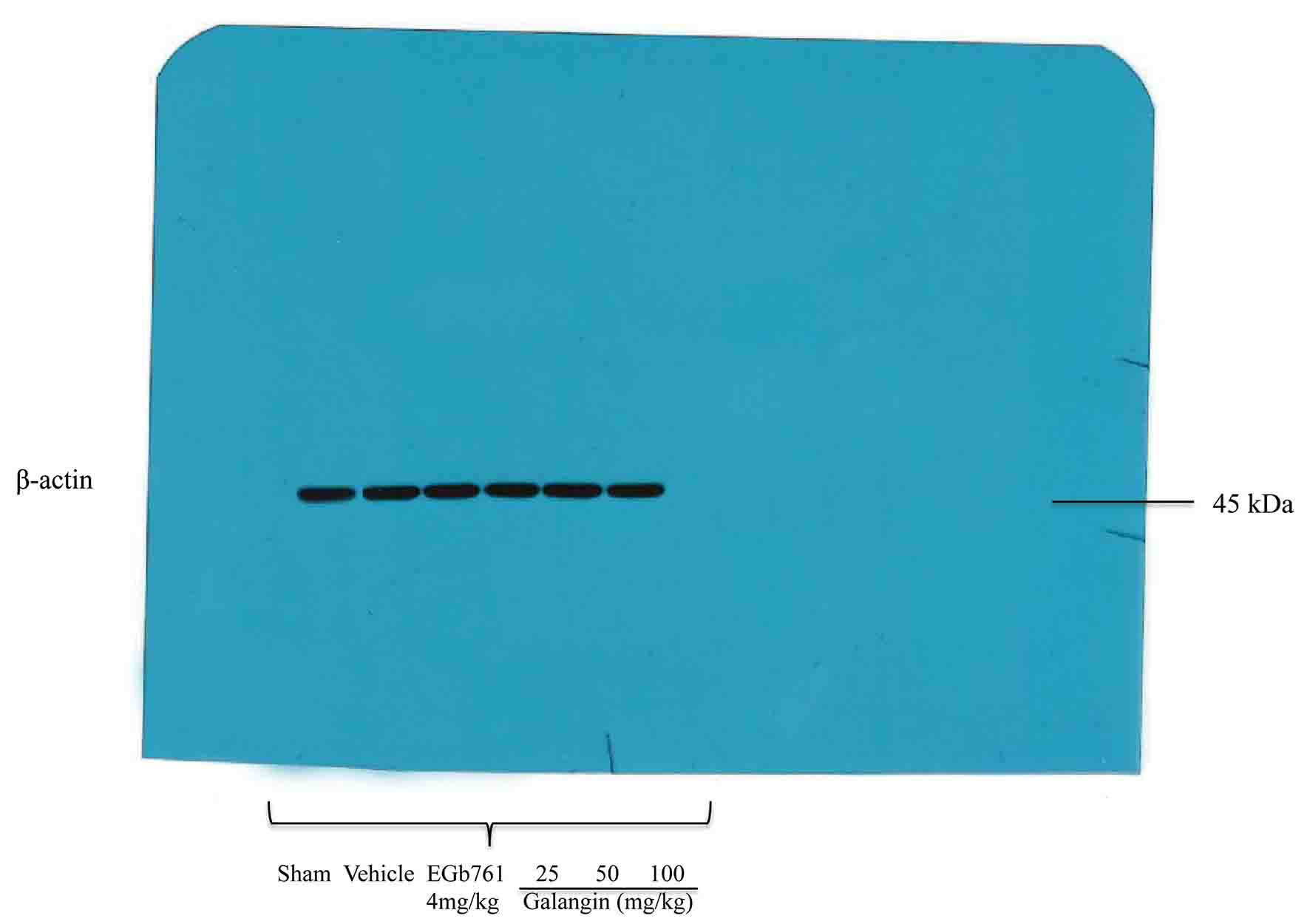


AM, 24 h


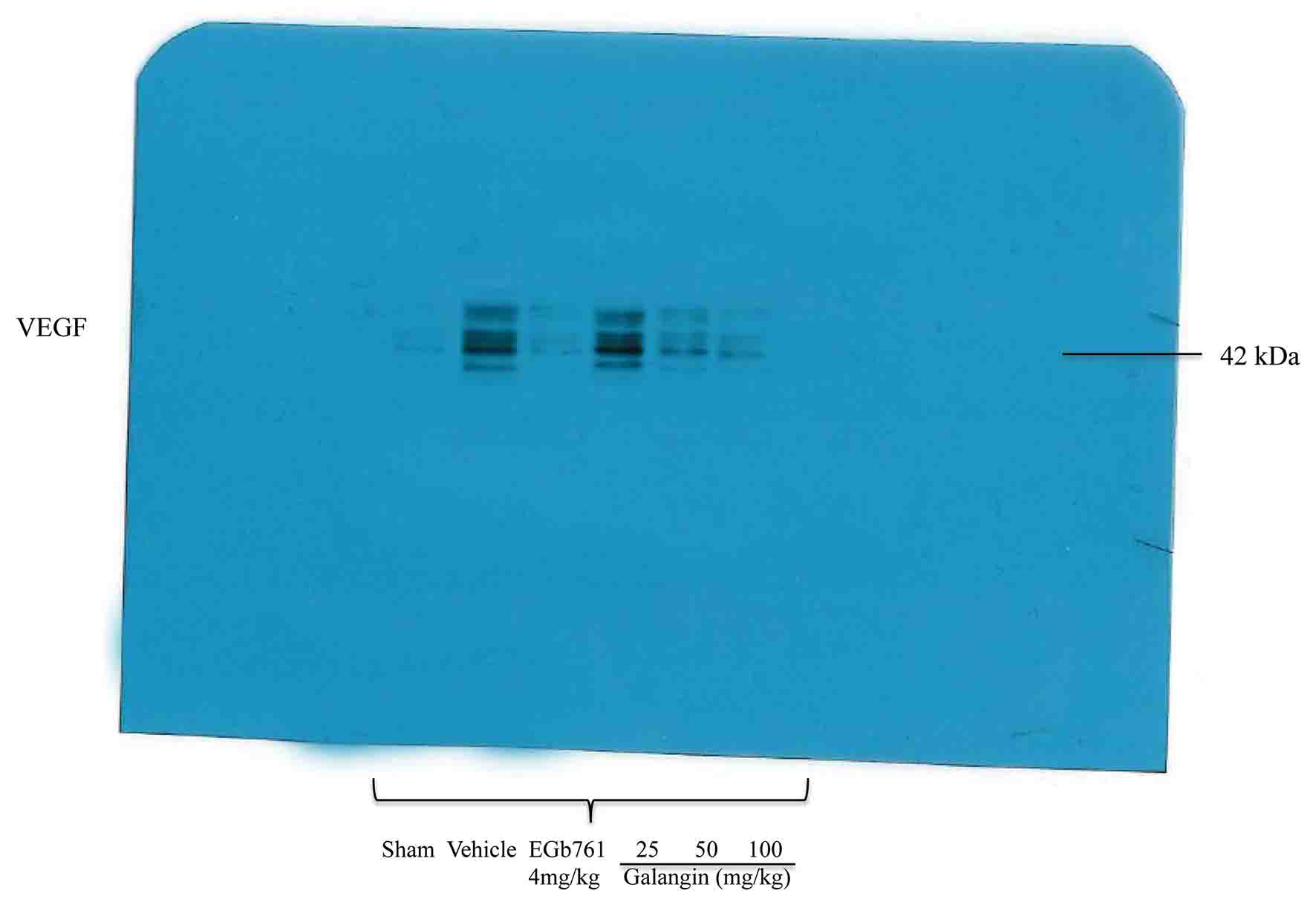


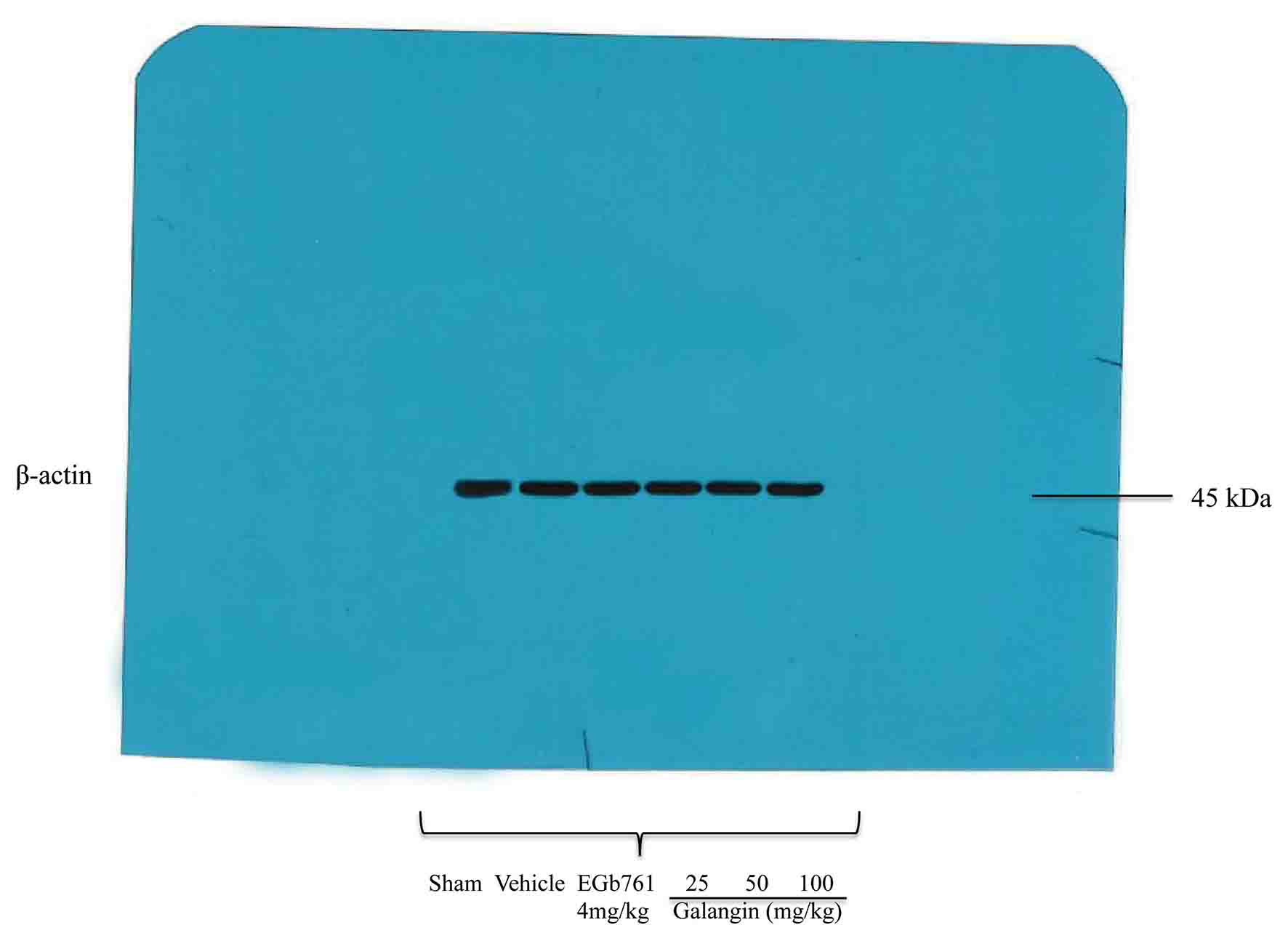


AQP-4:

PM, 12 h


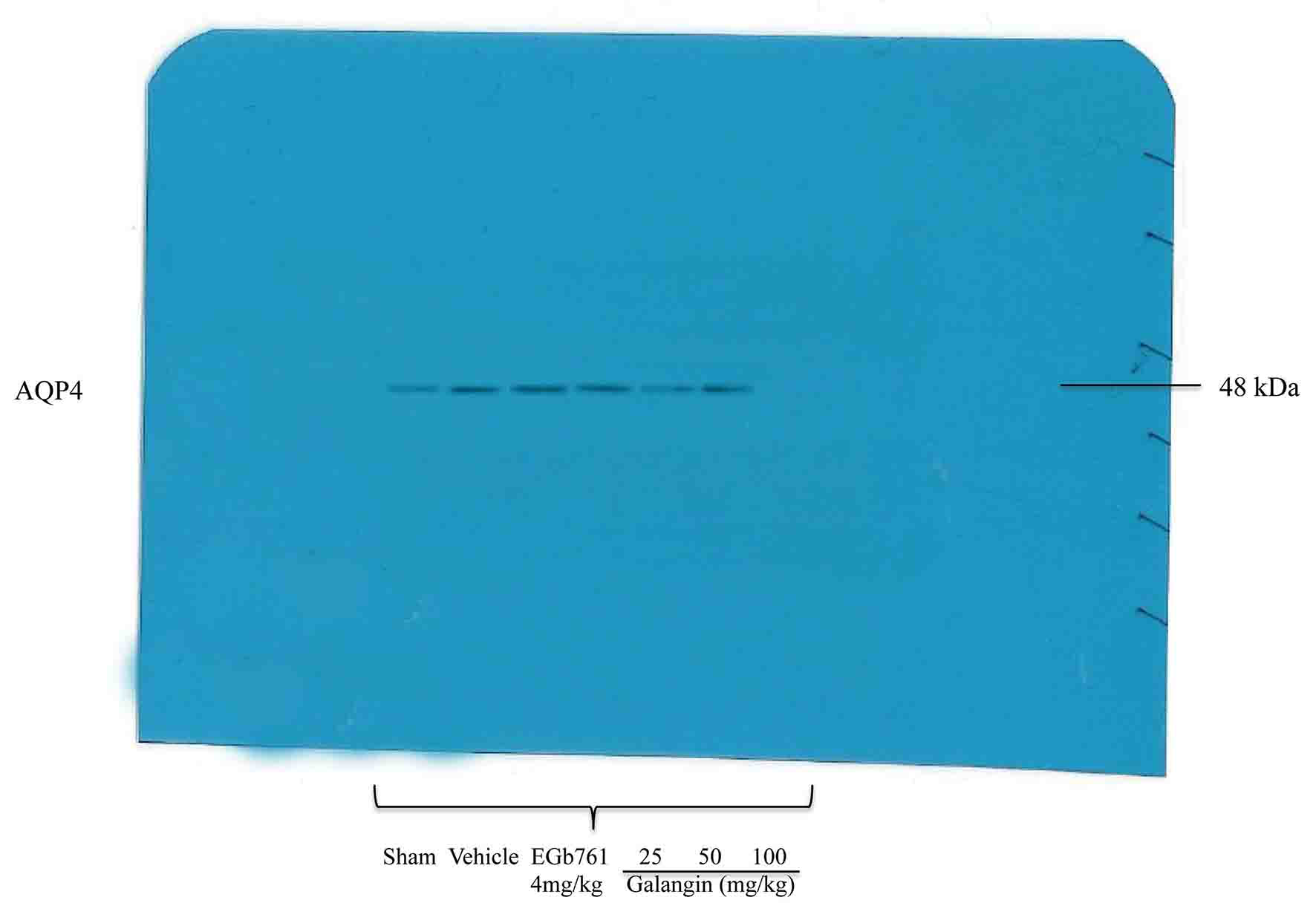


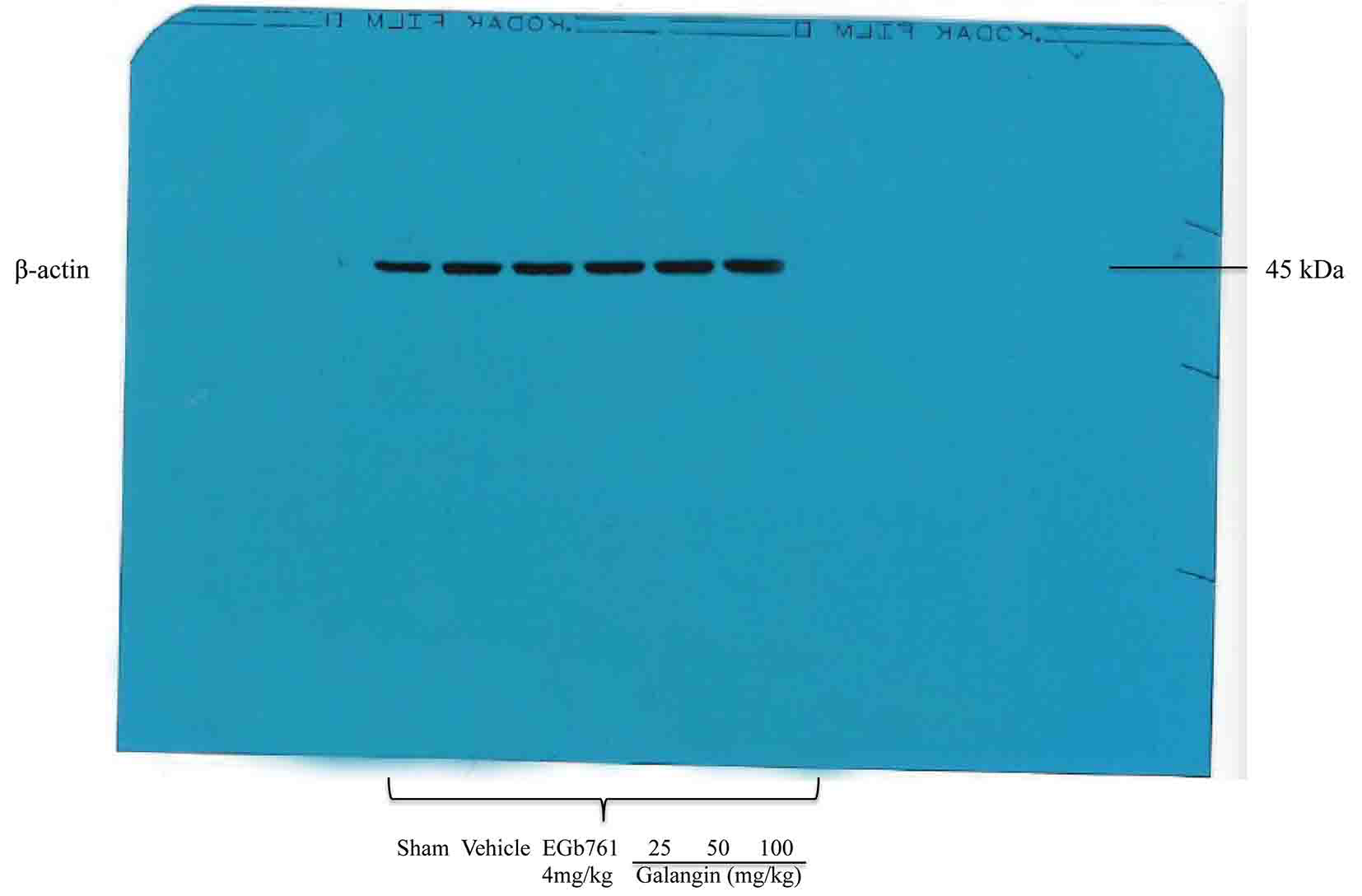


PM, 24 h


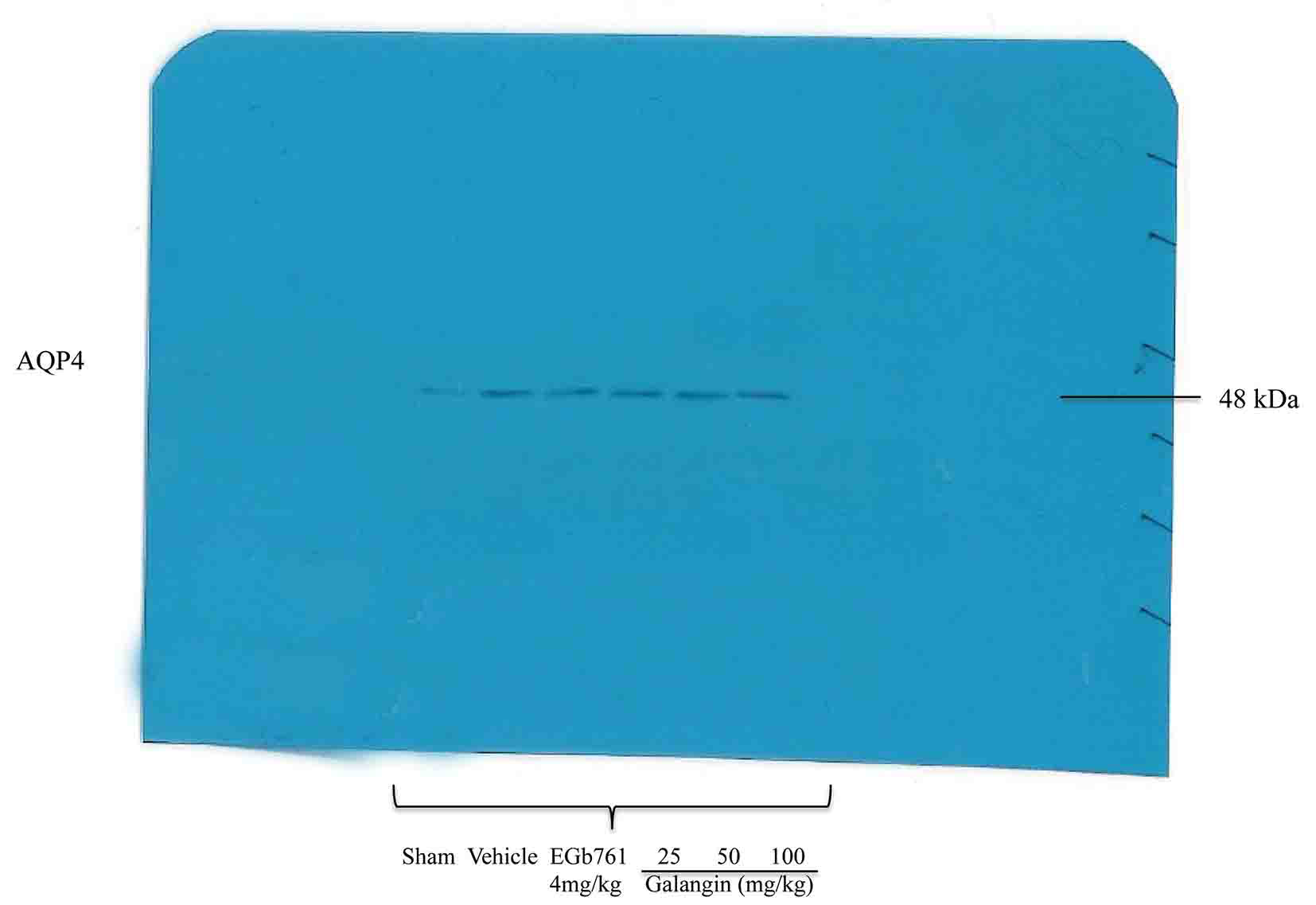


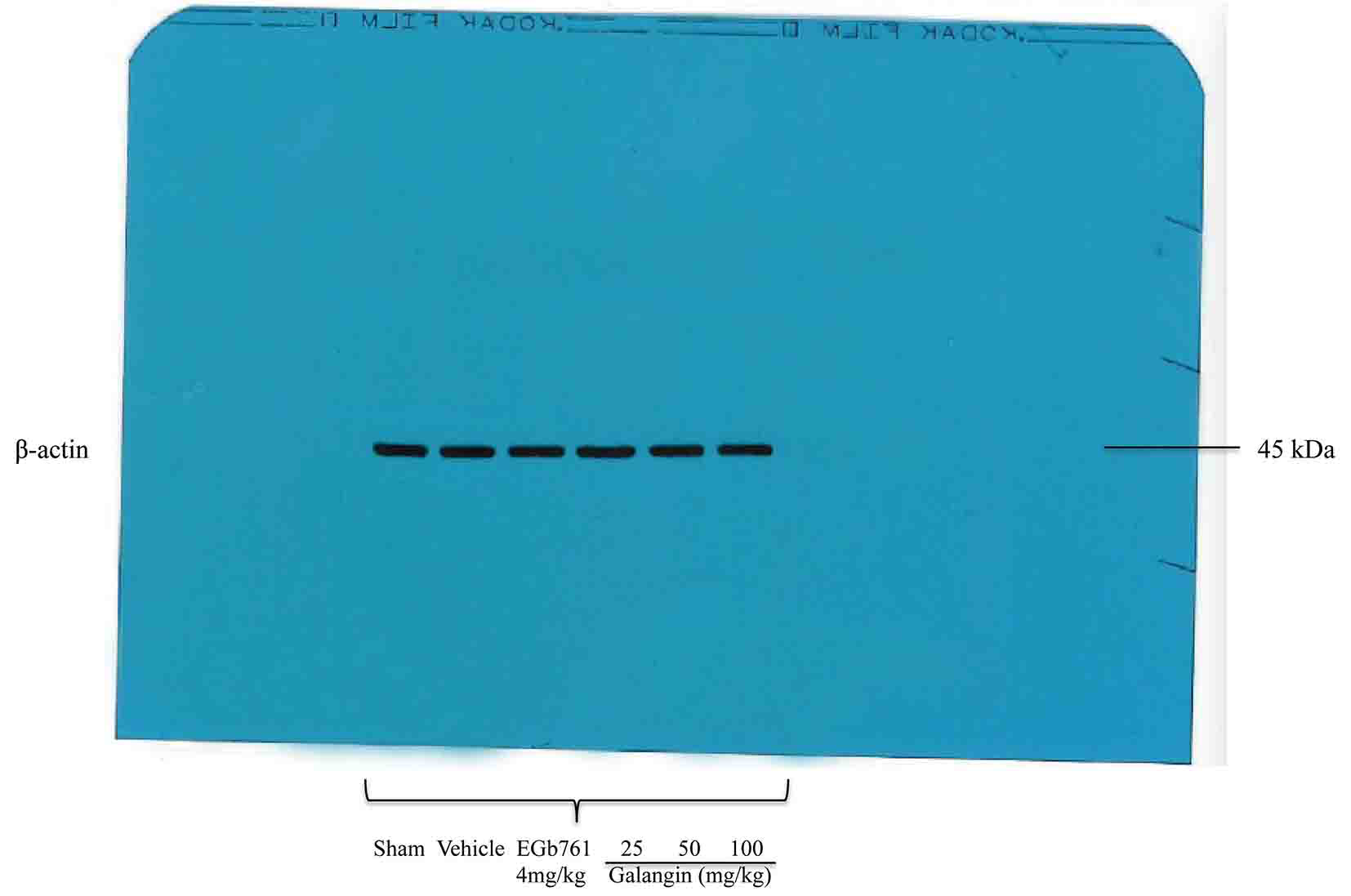


AM, 24 h


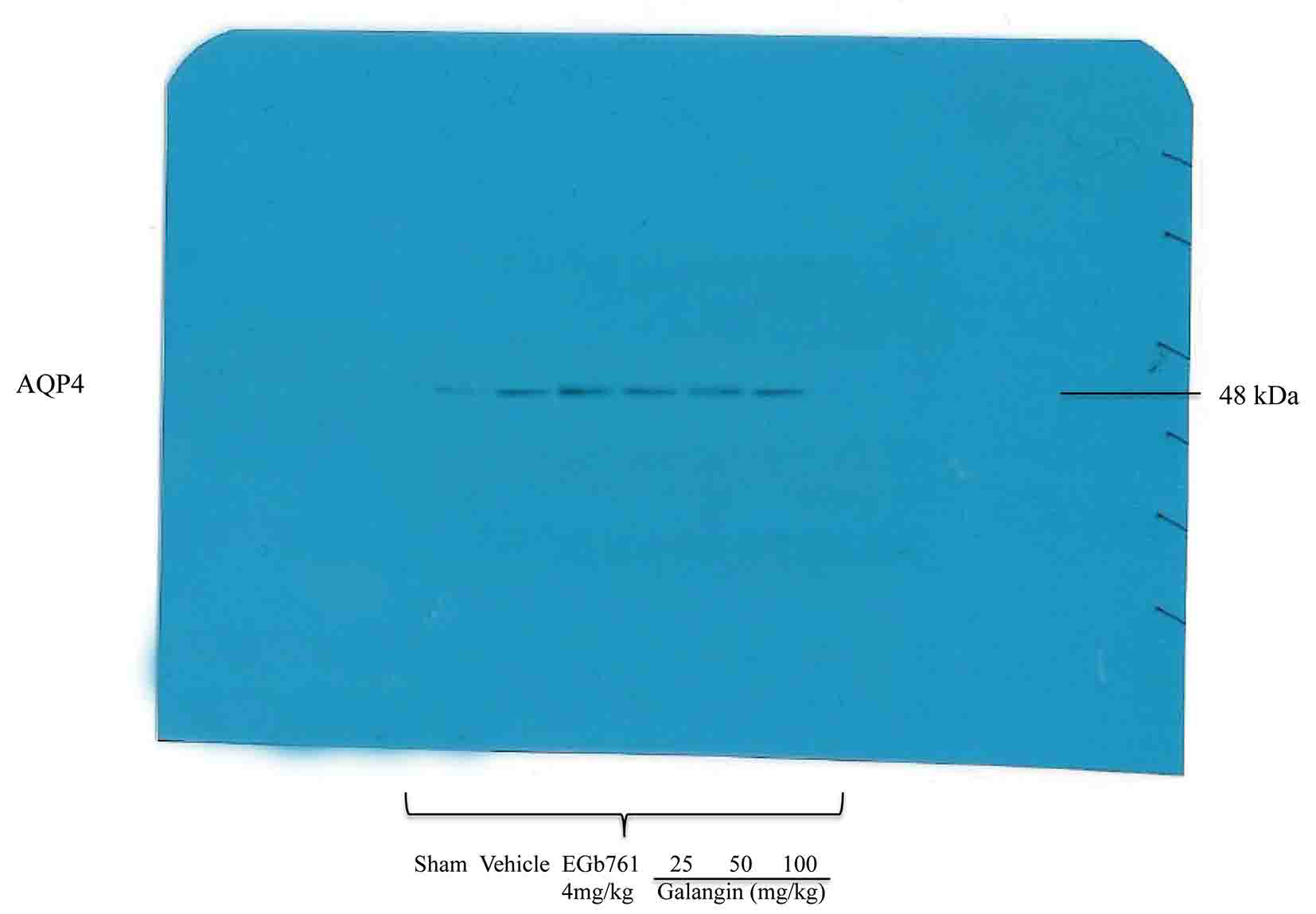


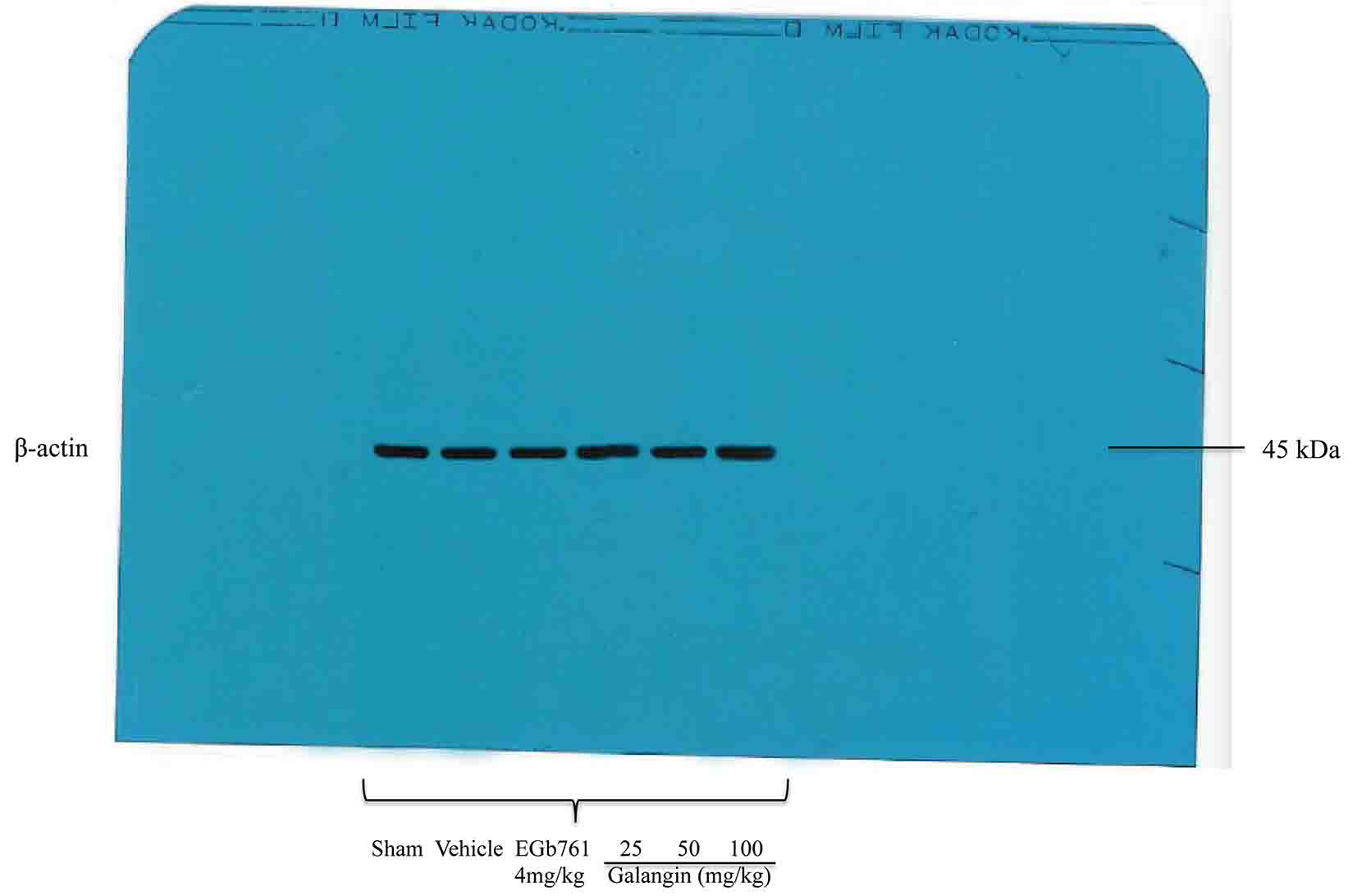


MMP-9:

PM, 12 h


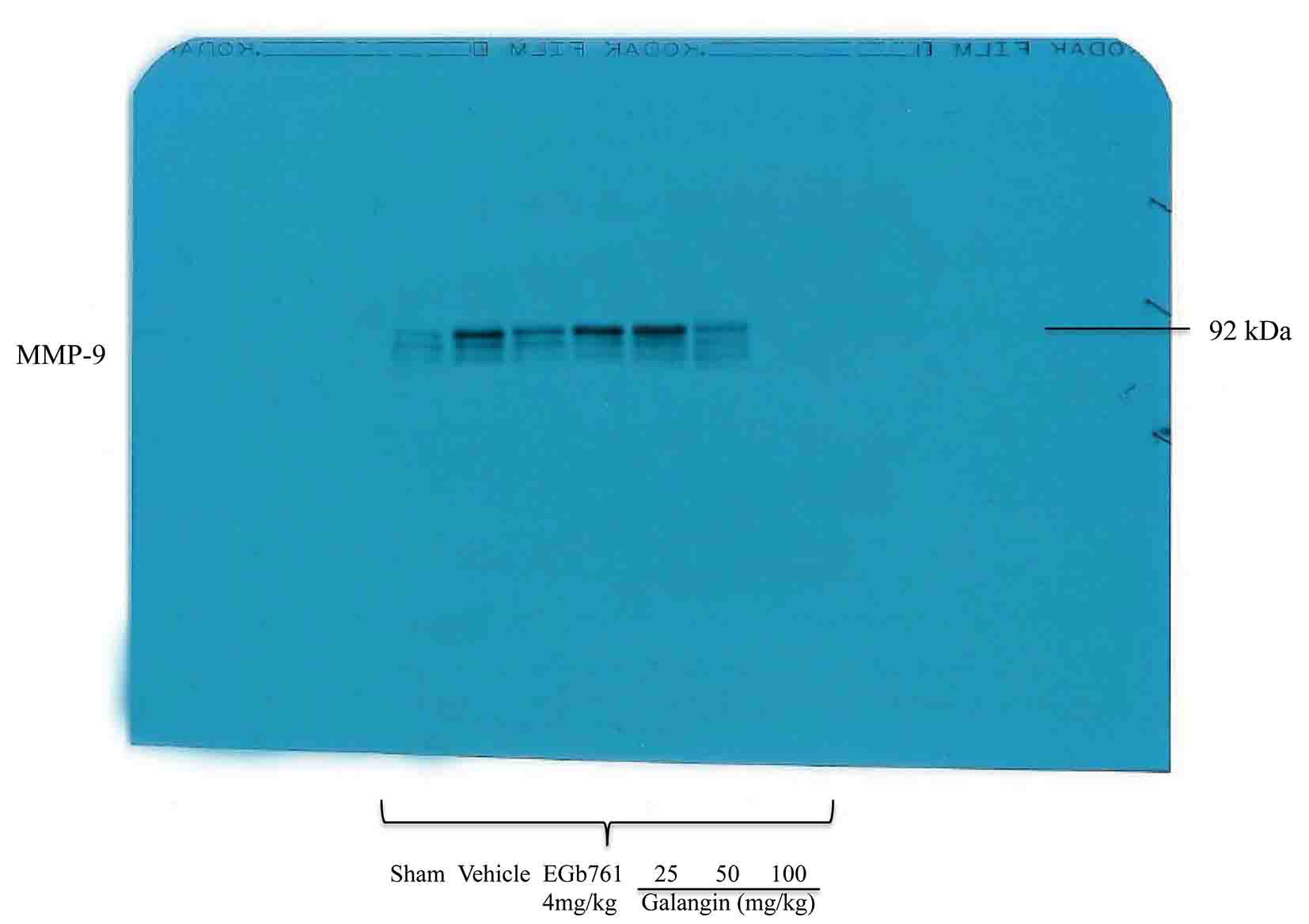


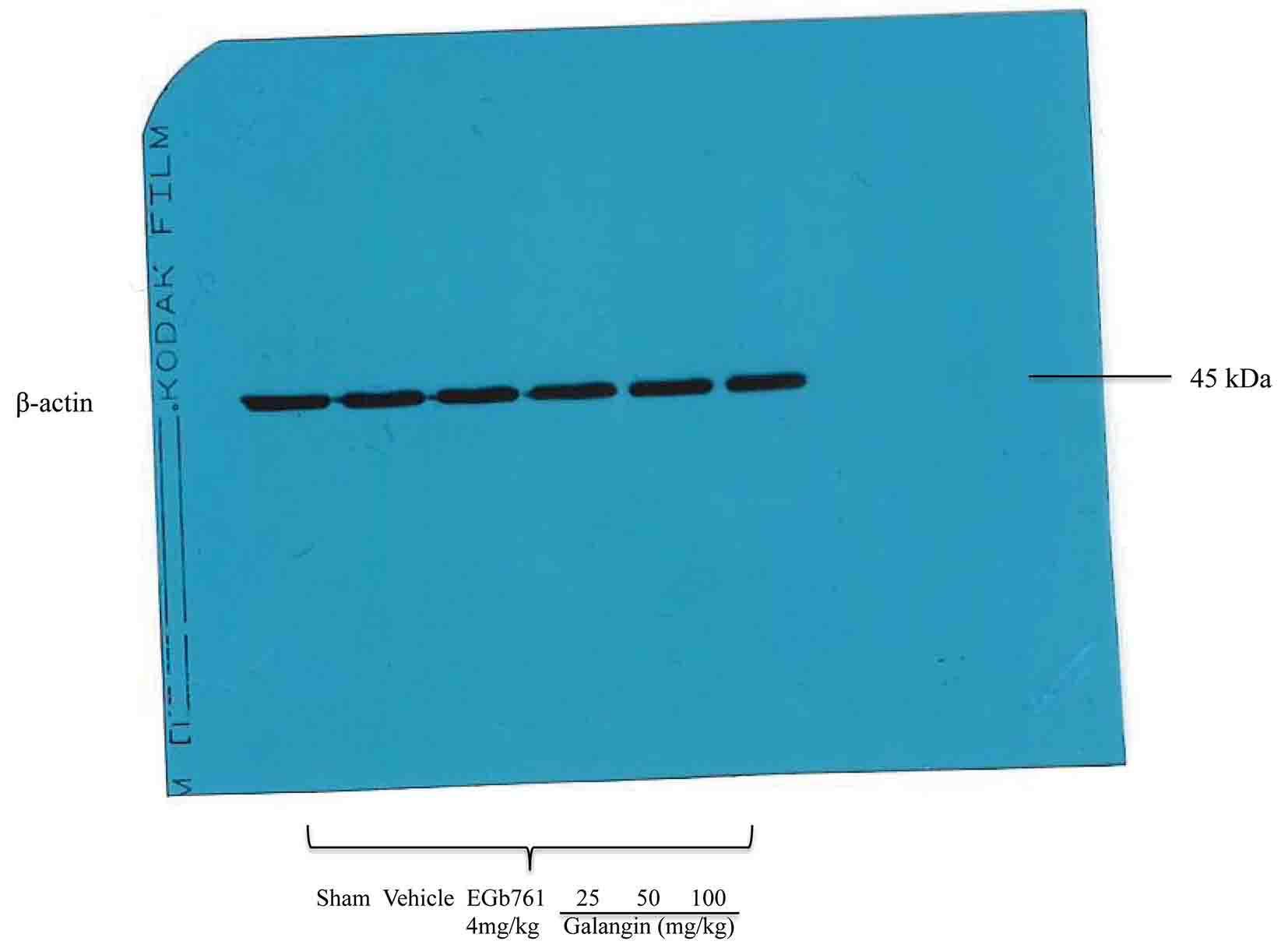


PM, 24 h


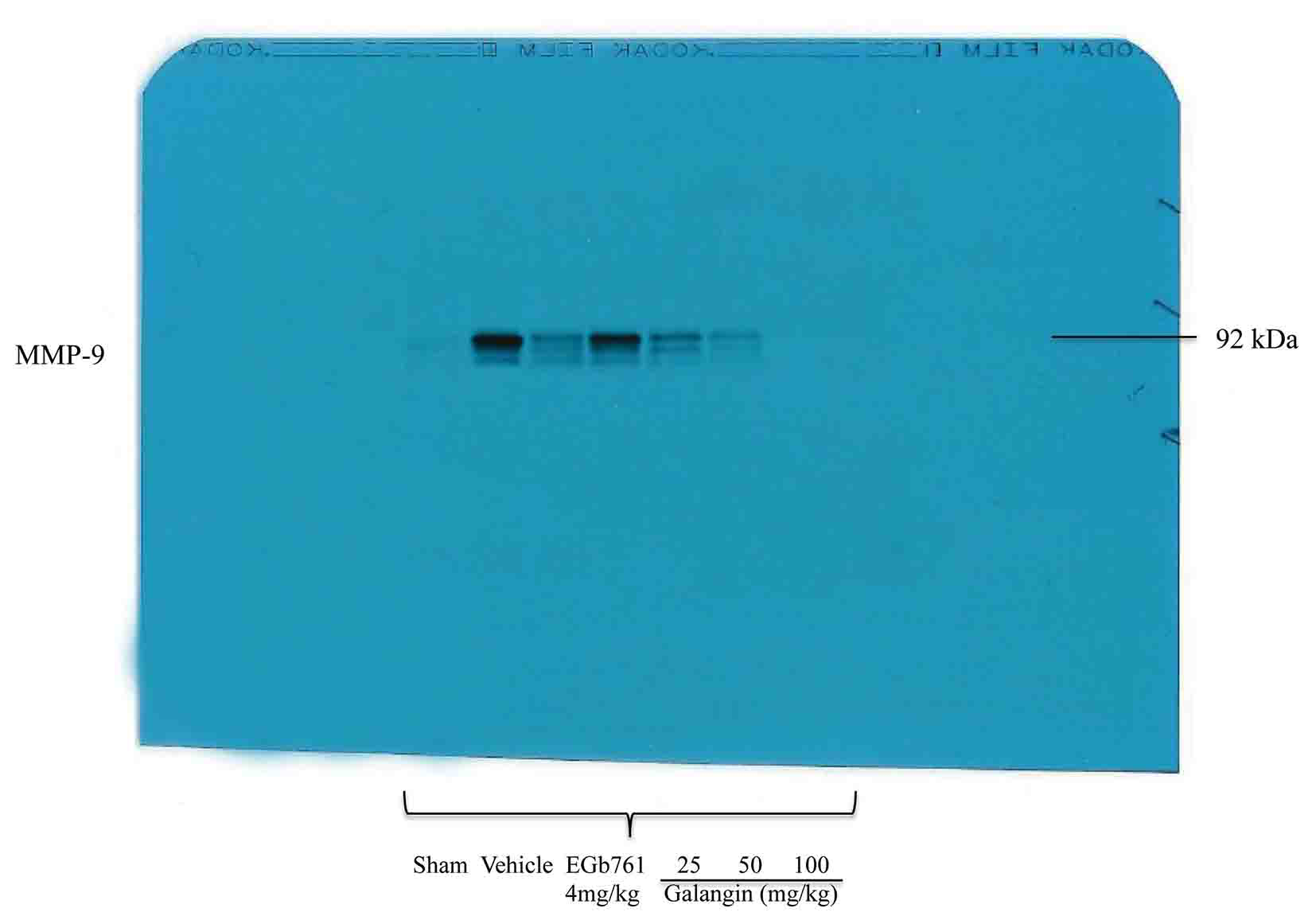


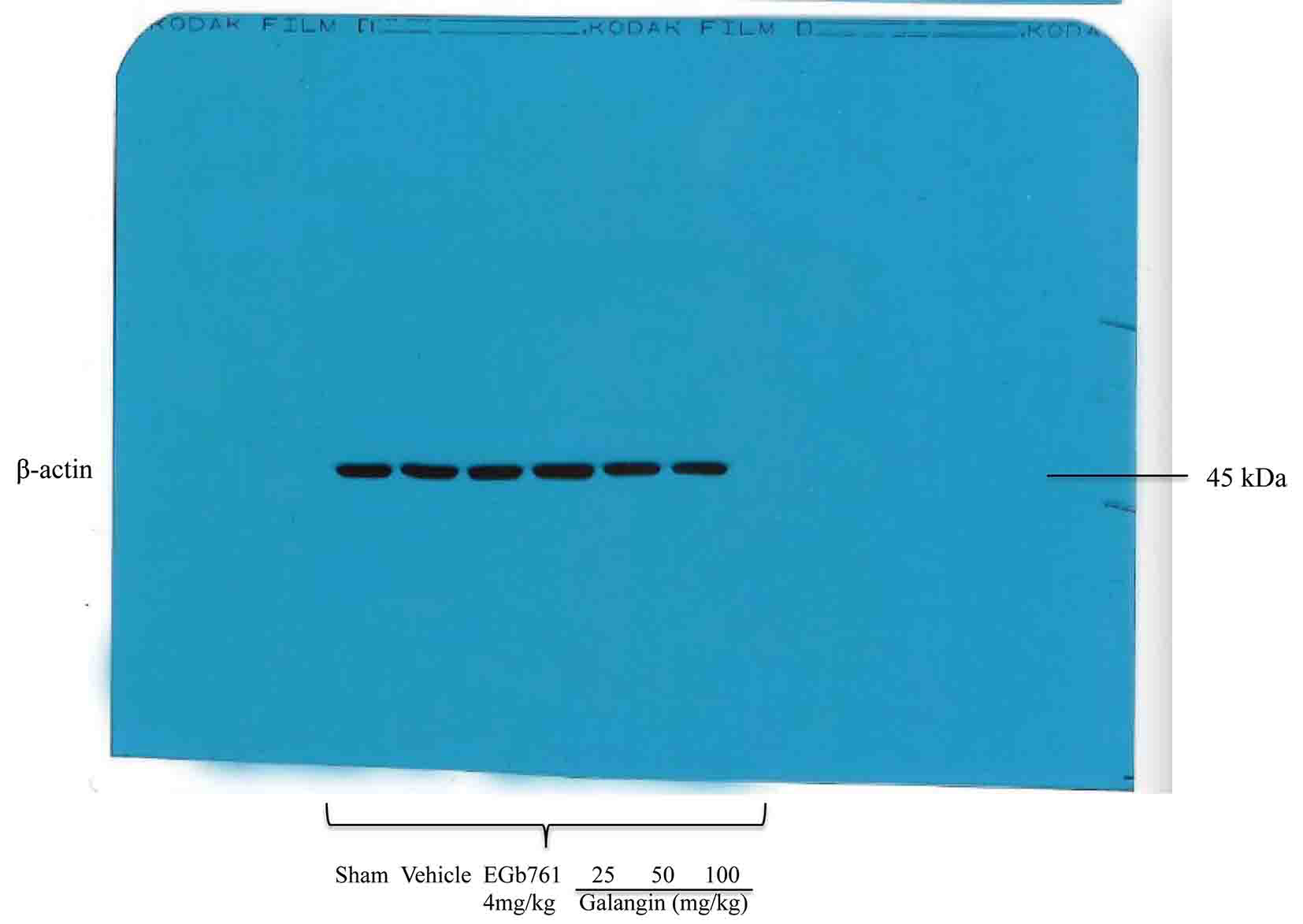


AM, 24 h


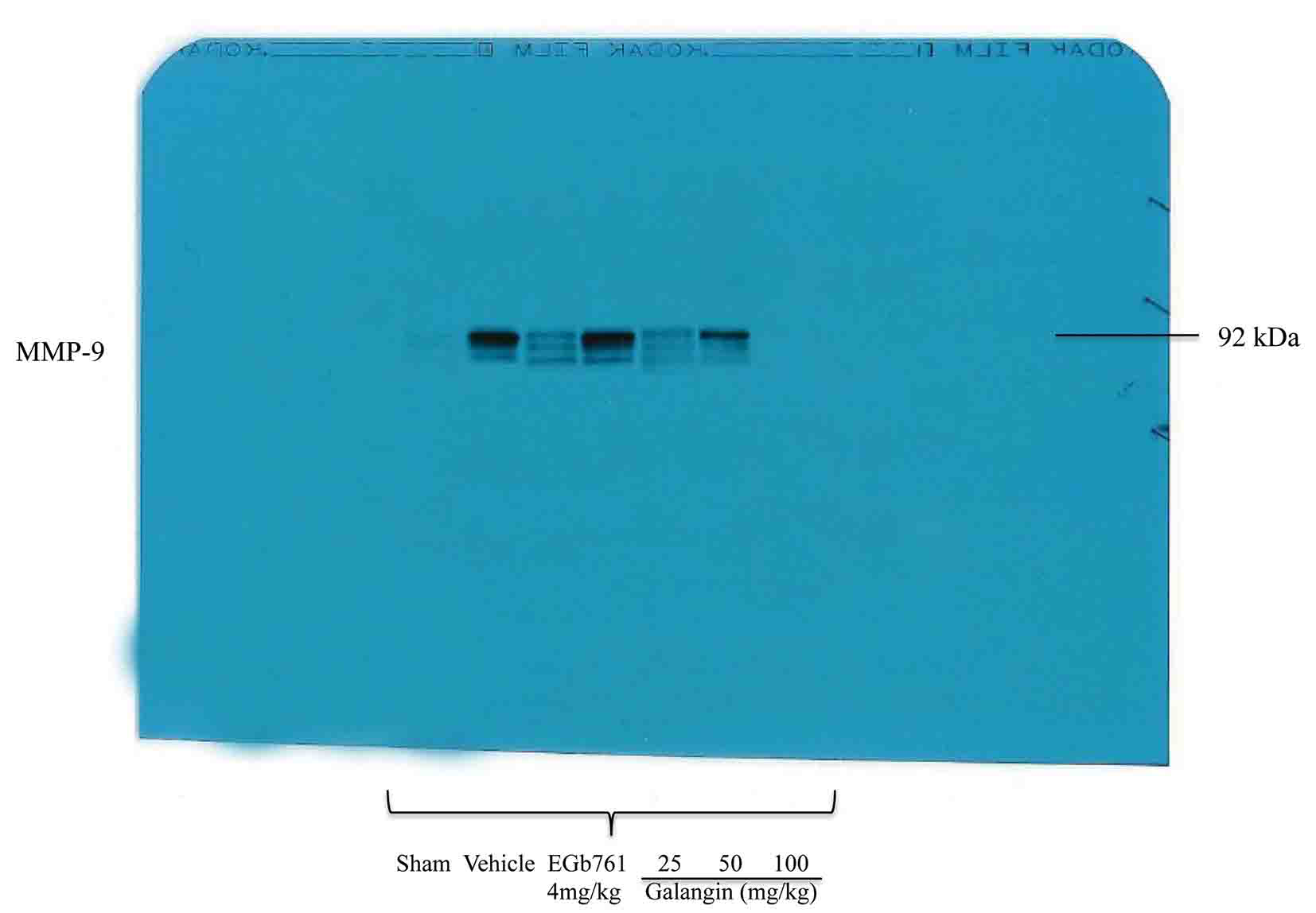


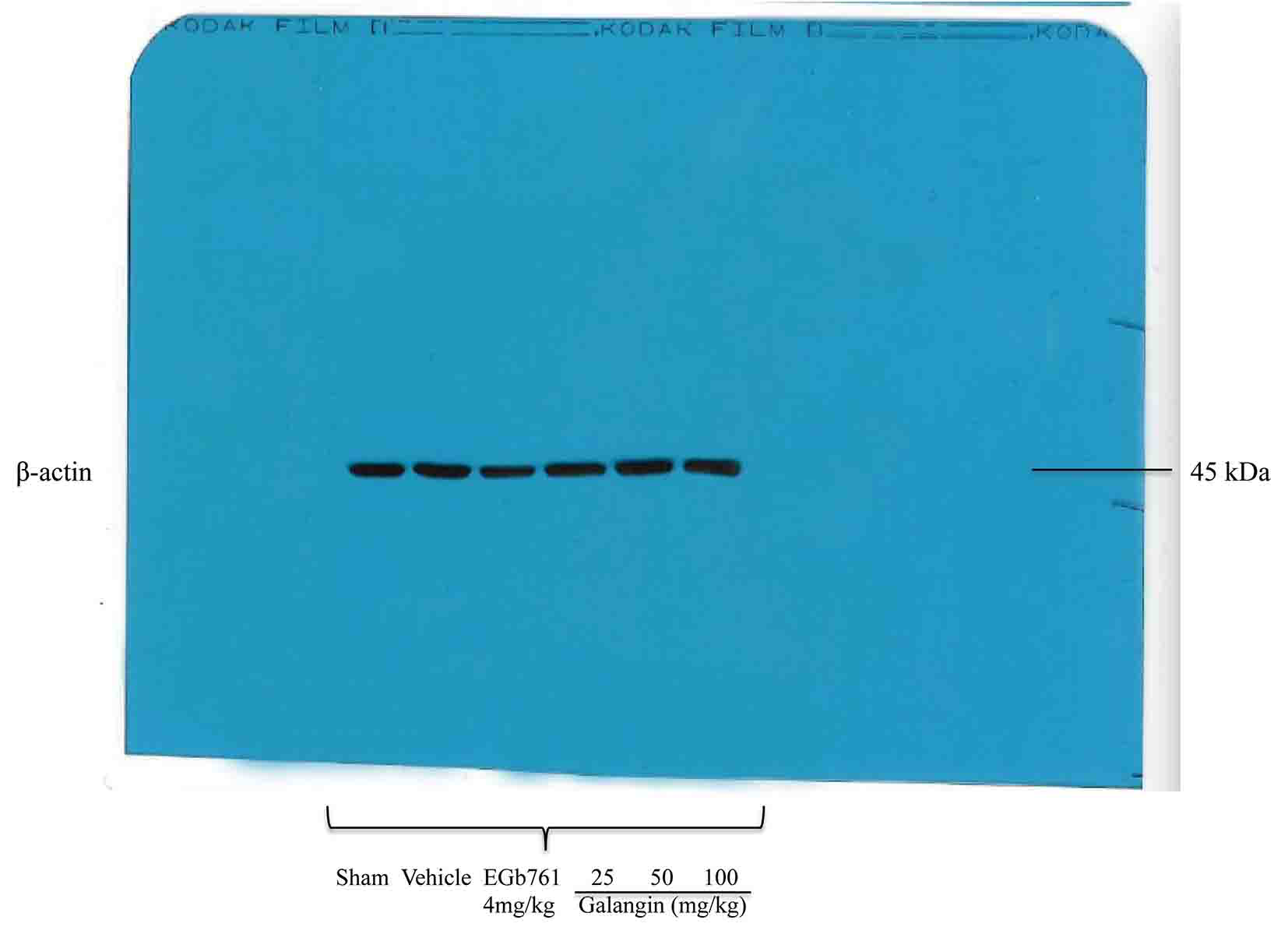


p-GSK-3β:

PM, 12 h


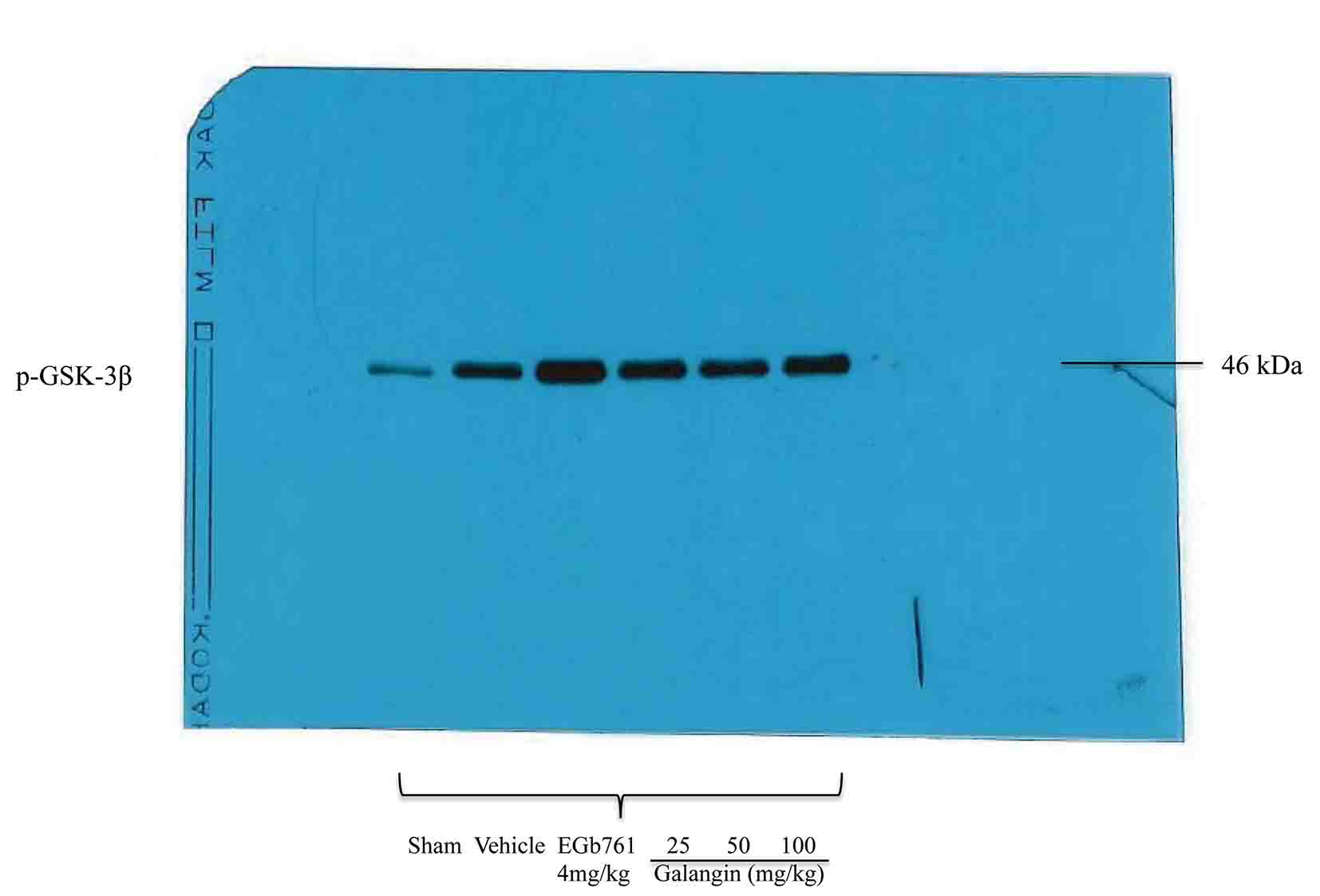


PM, 24 h


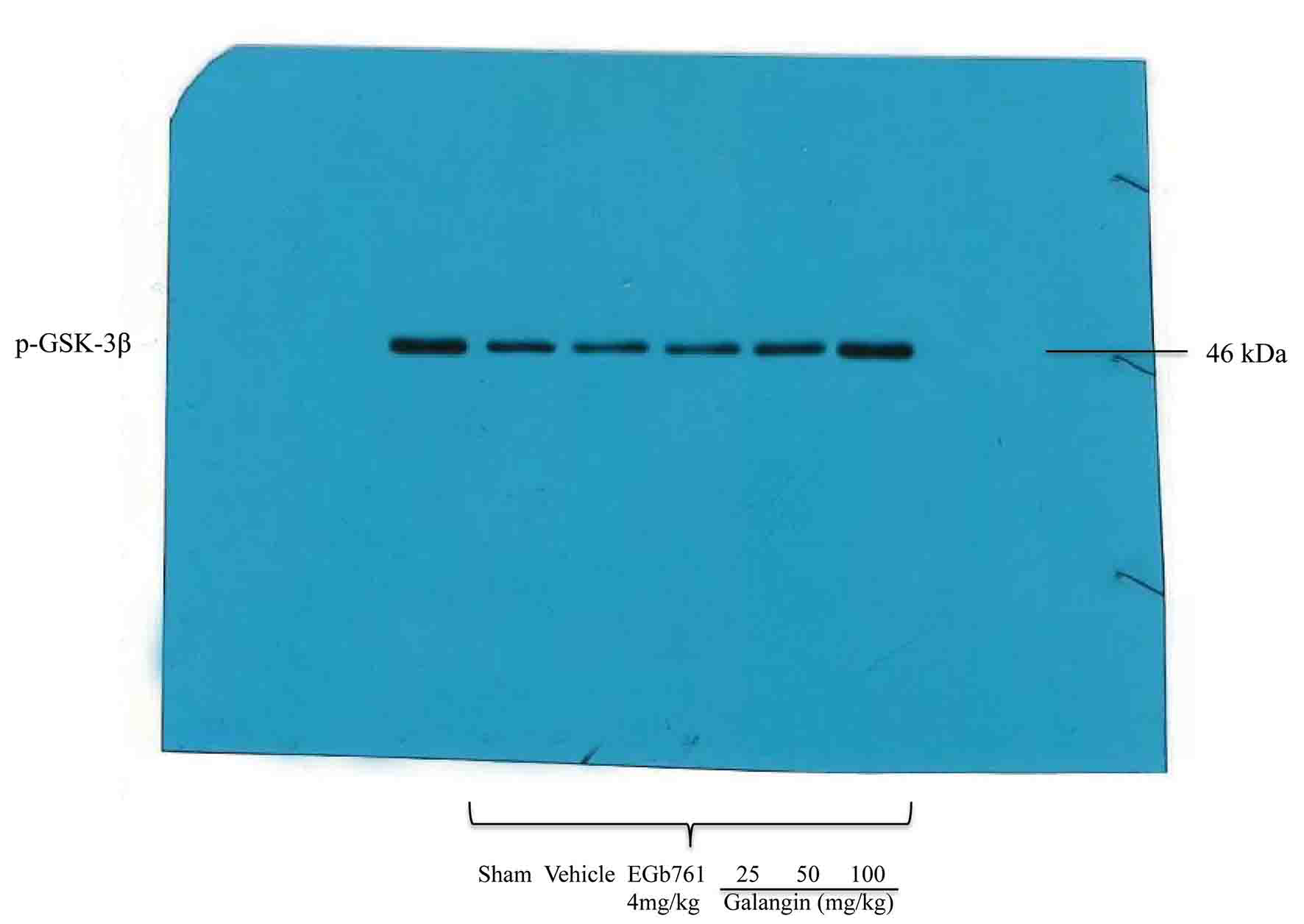


AM, 24 h


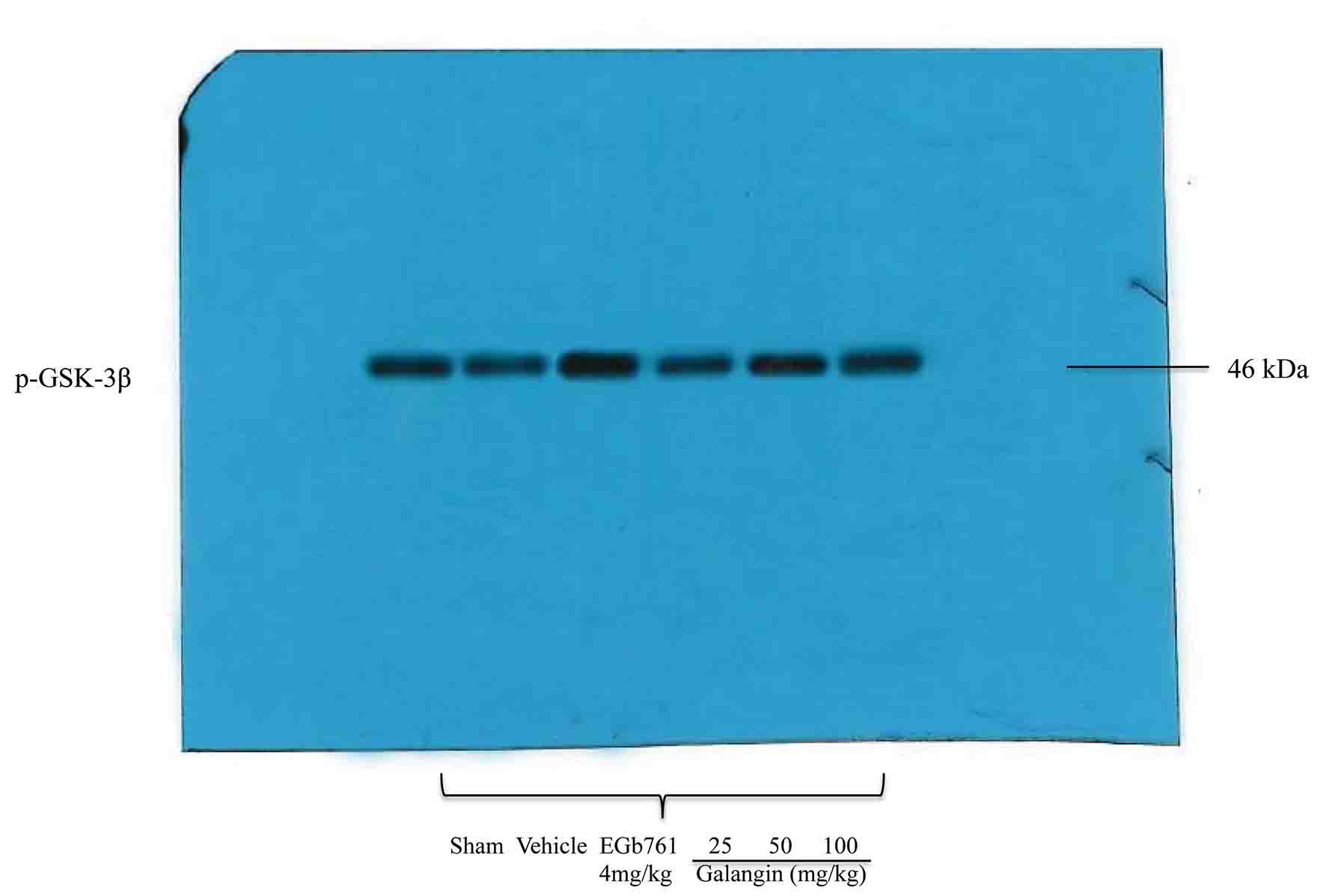


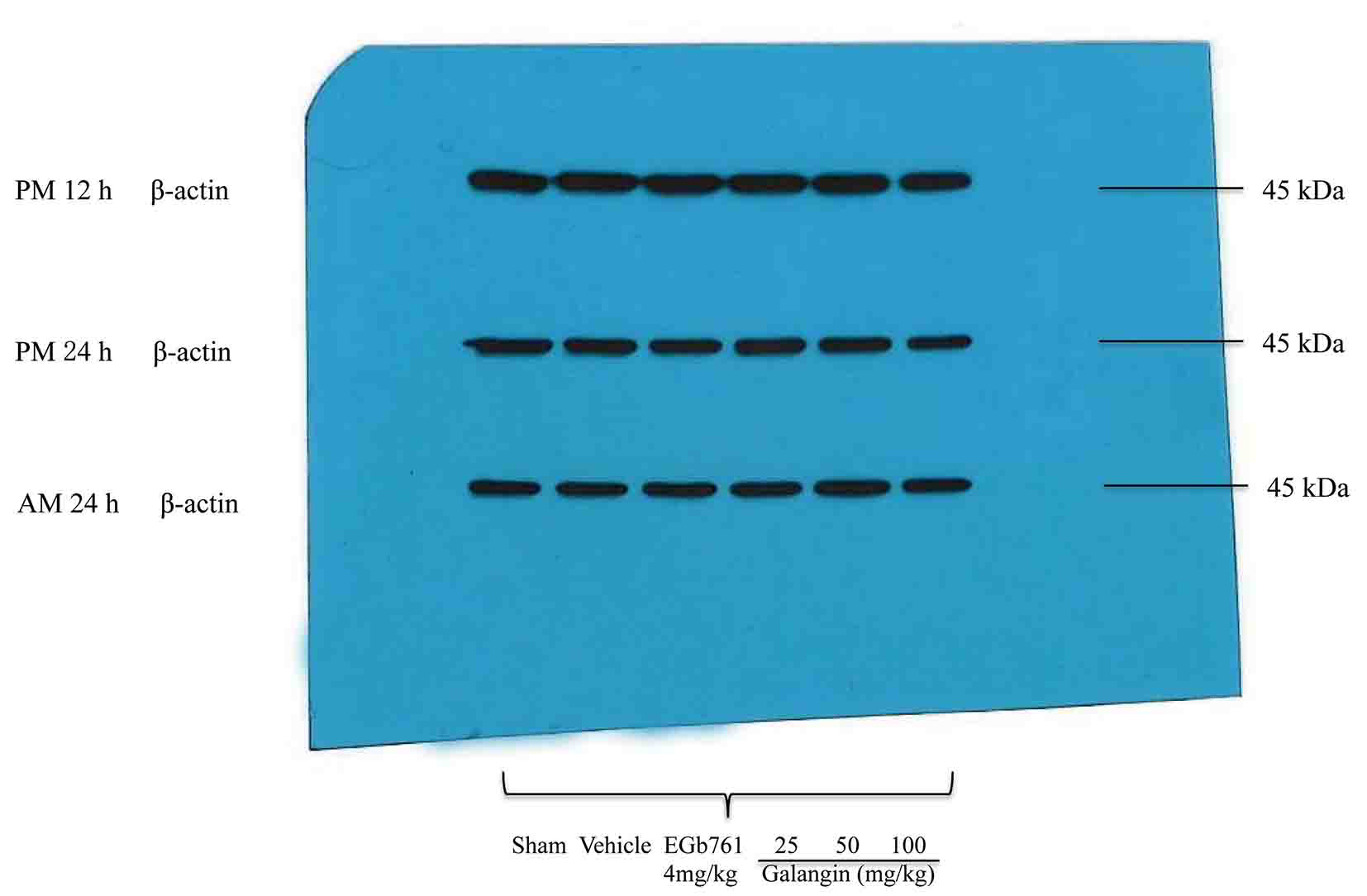


GSK3β:

PM, 12 h


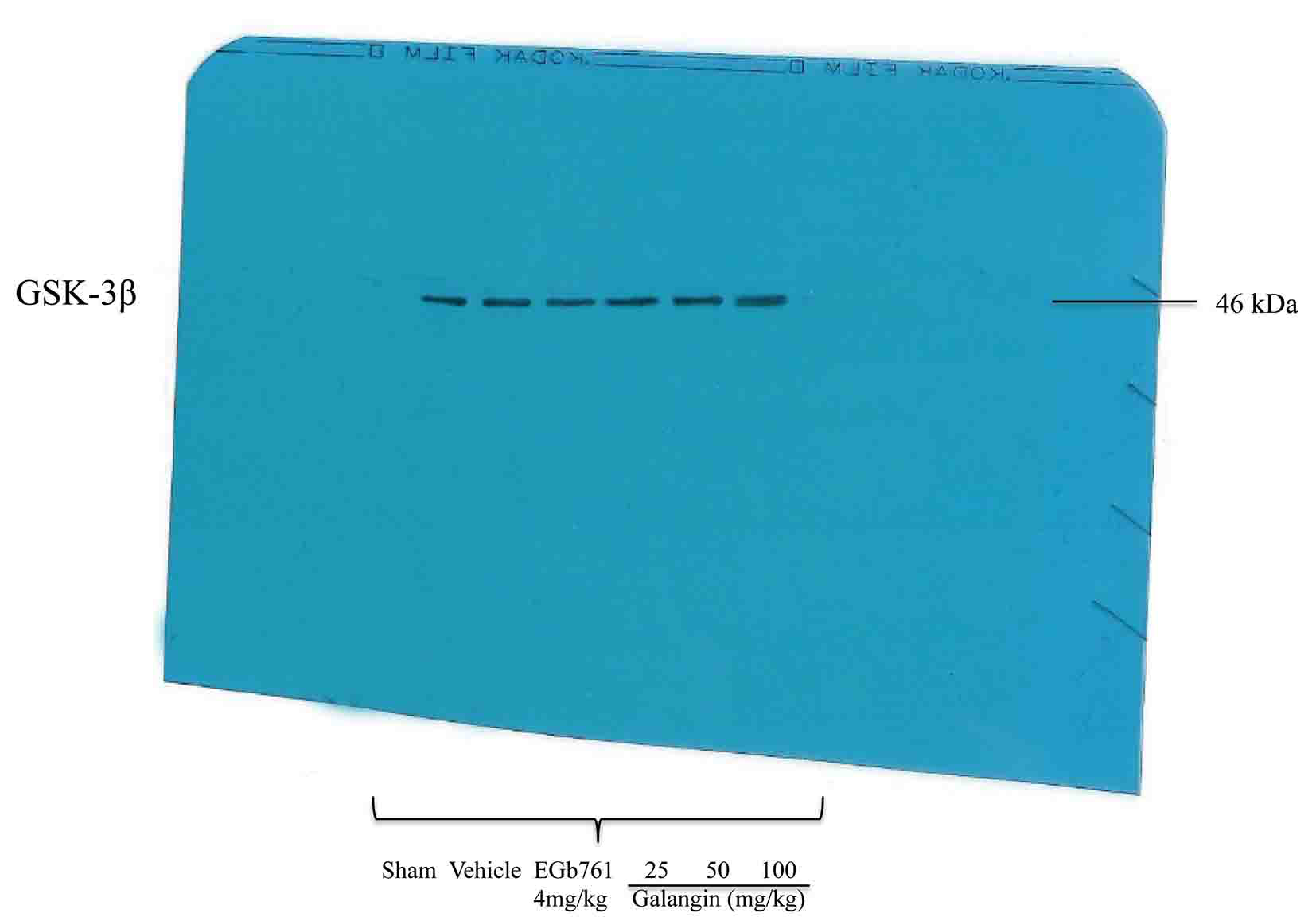


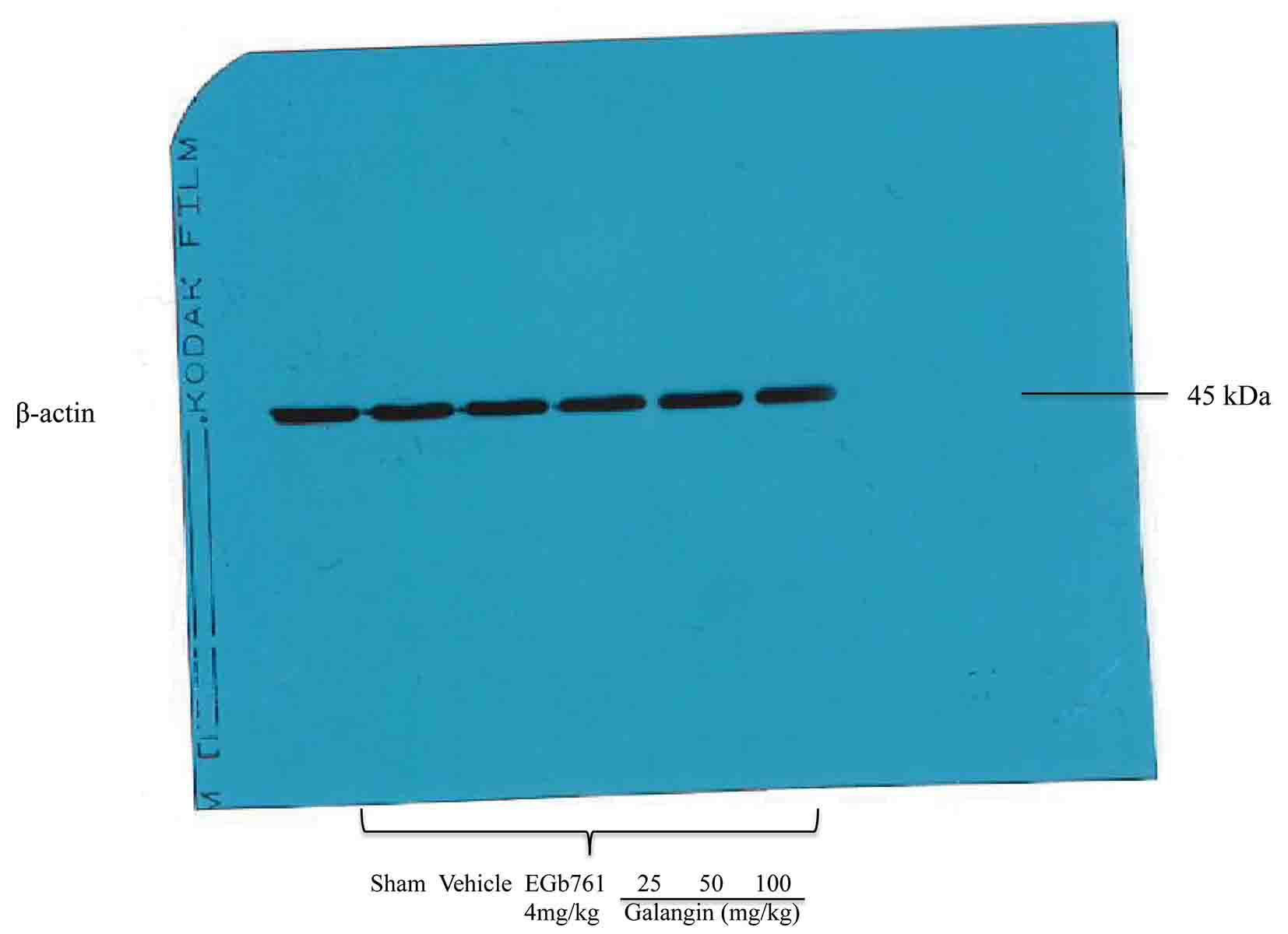


PM, 24 h


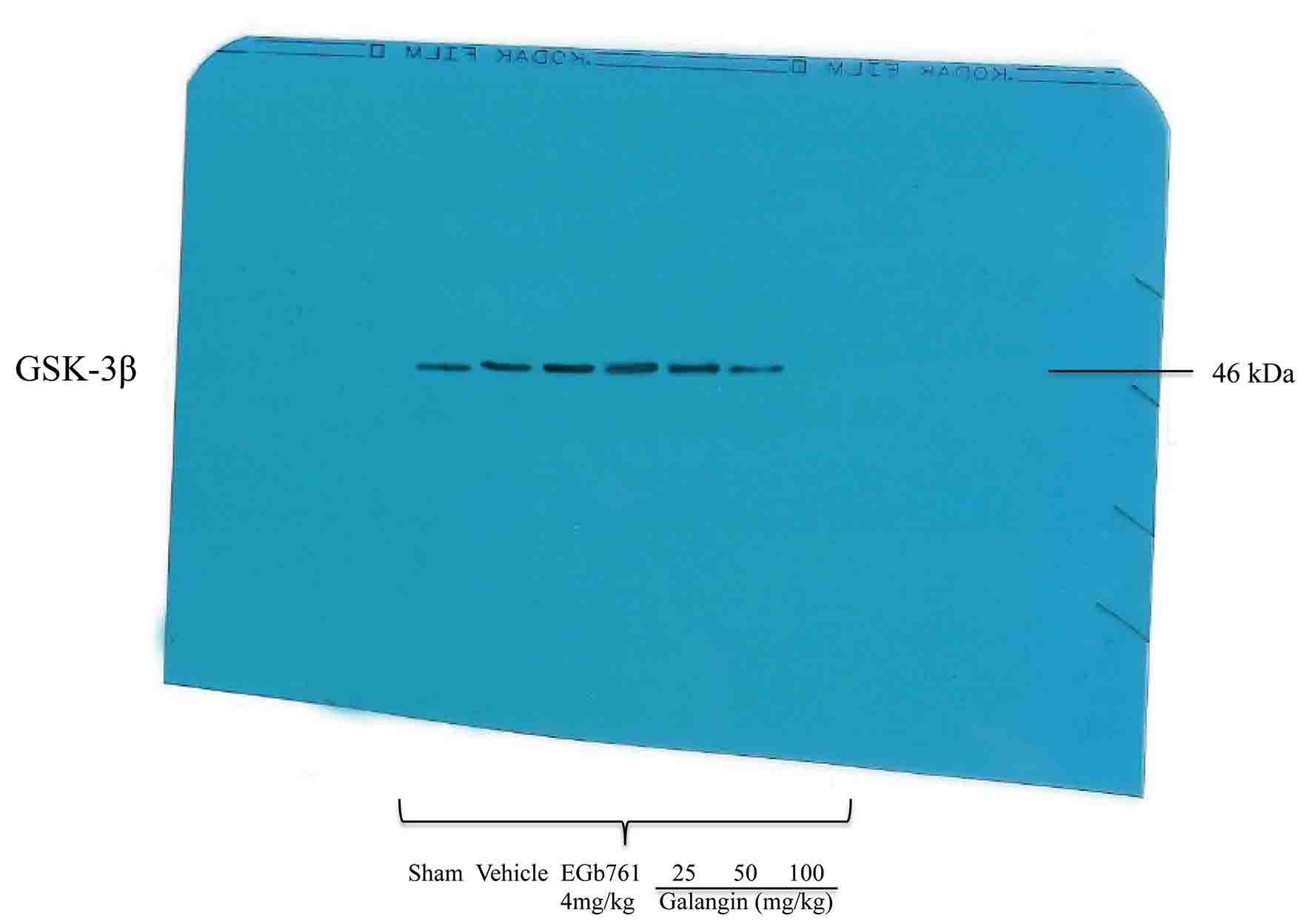


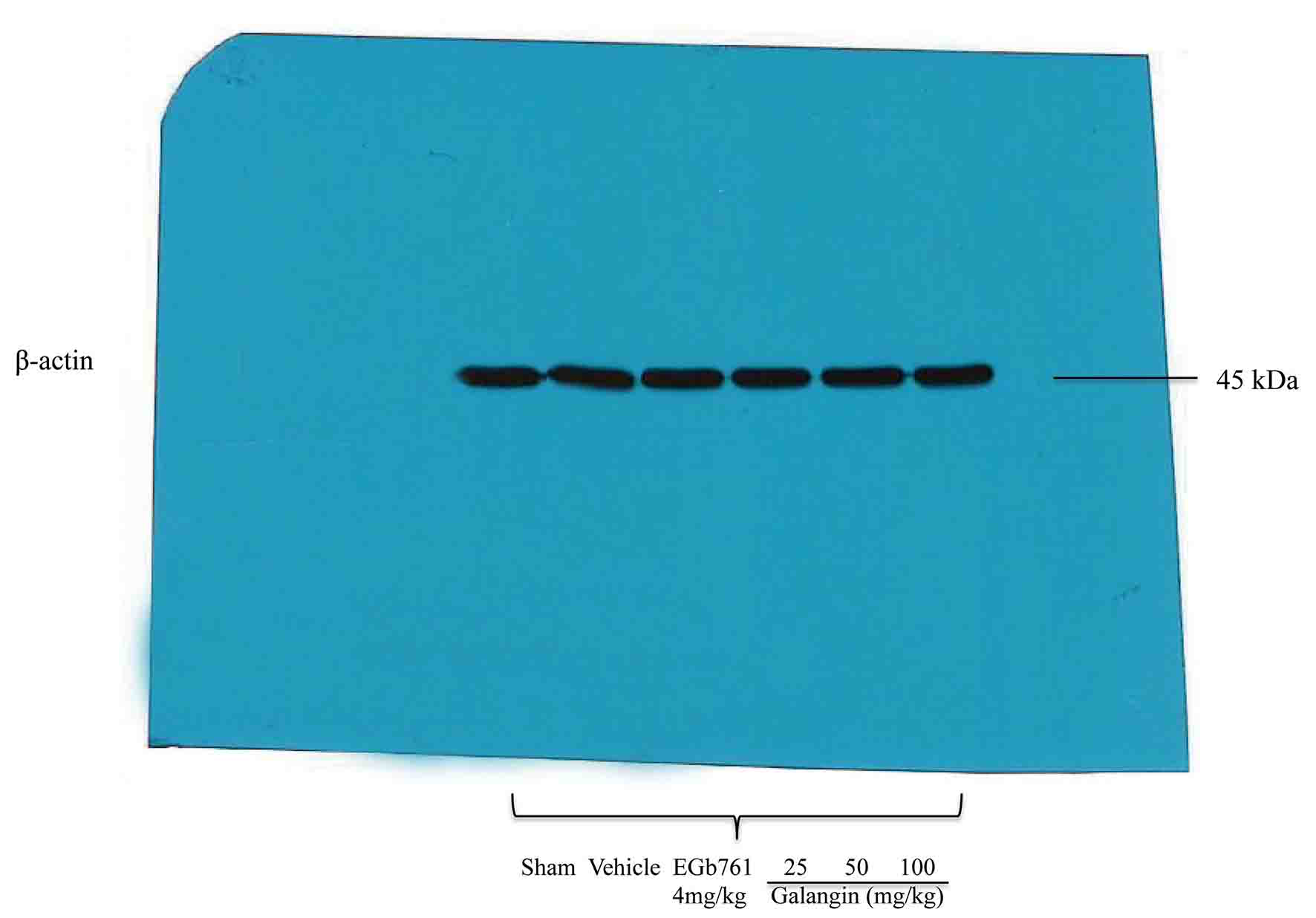


AM, 24 h


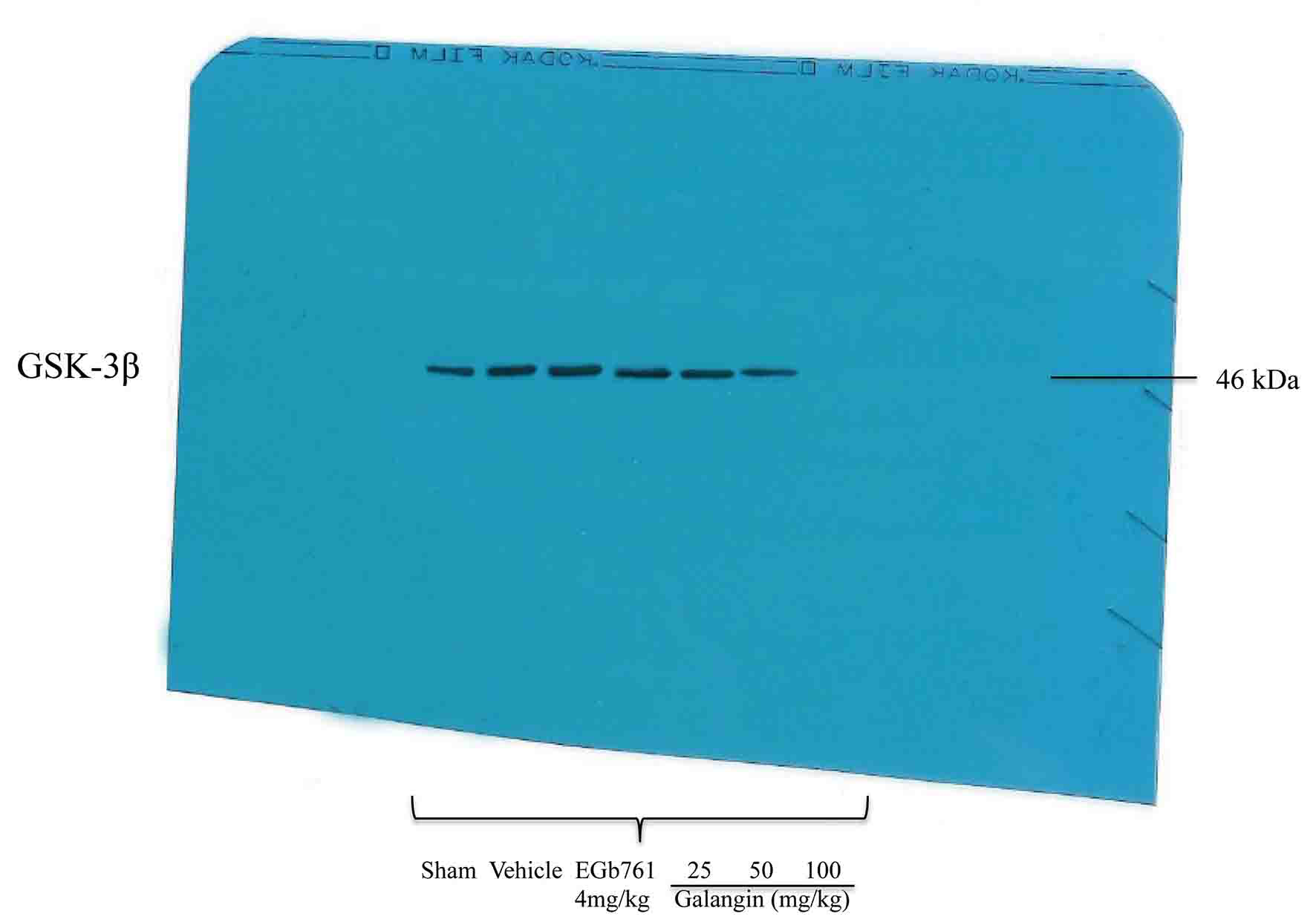


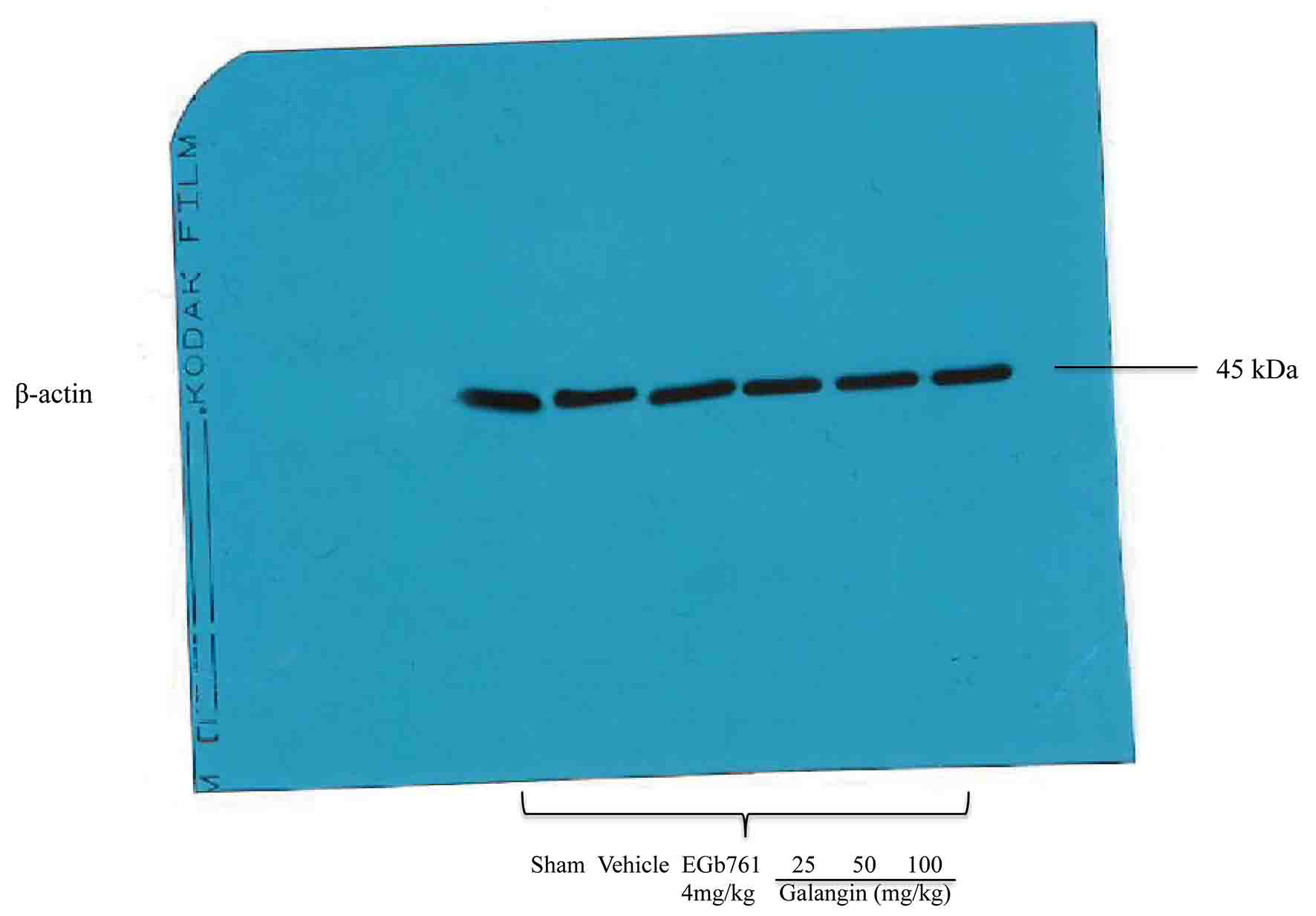


p-β-catenin:

PM, 12 h


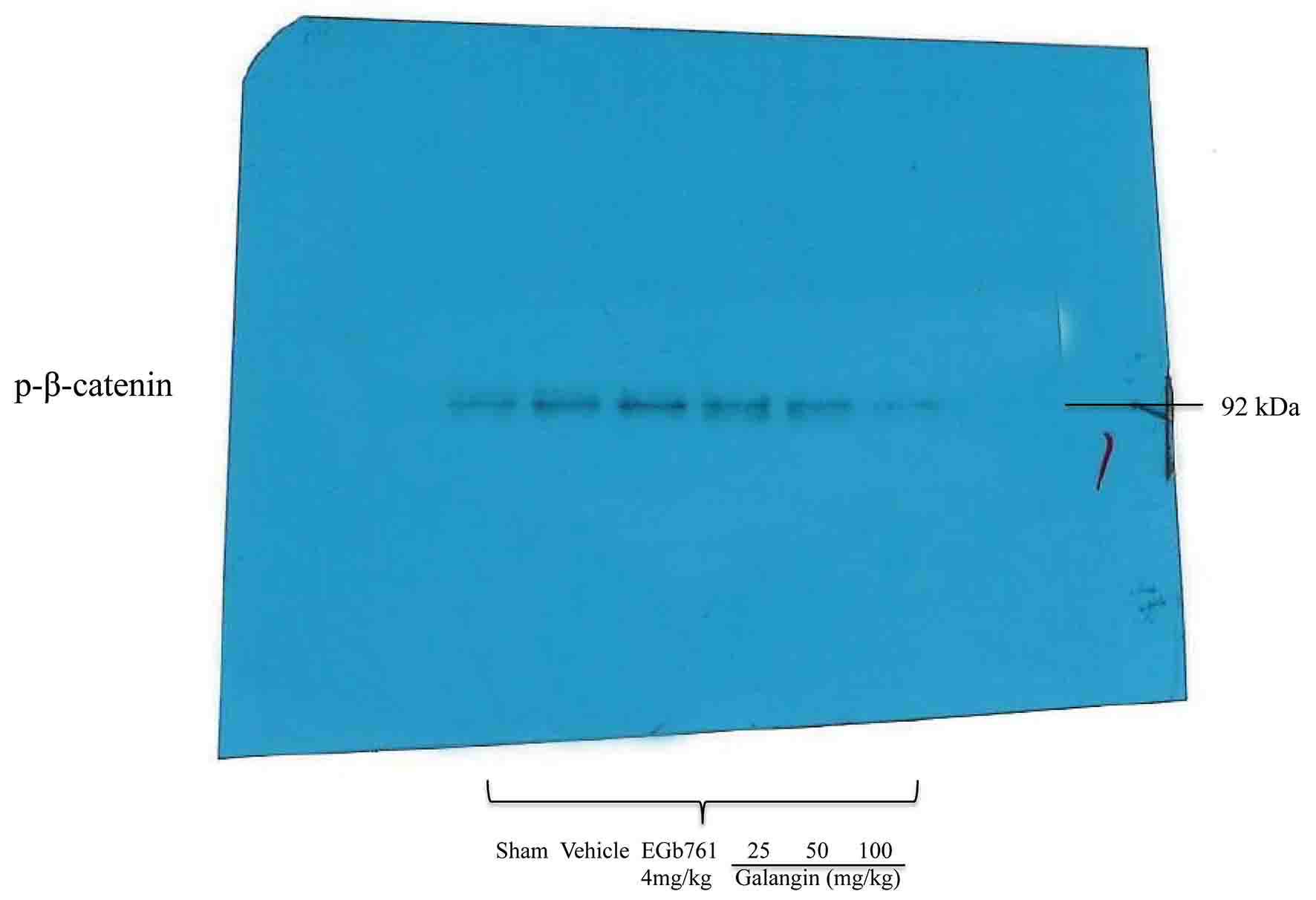


PM, 24 h


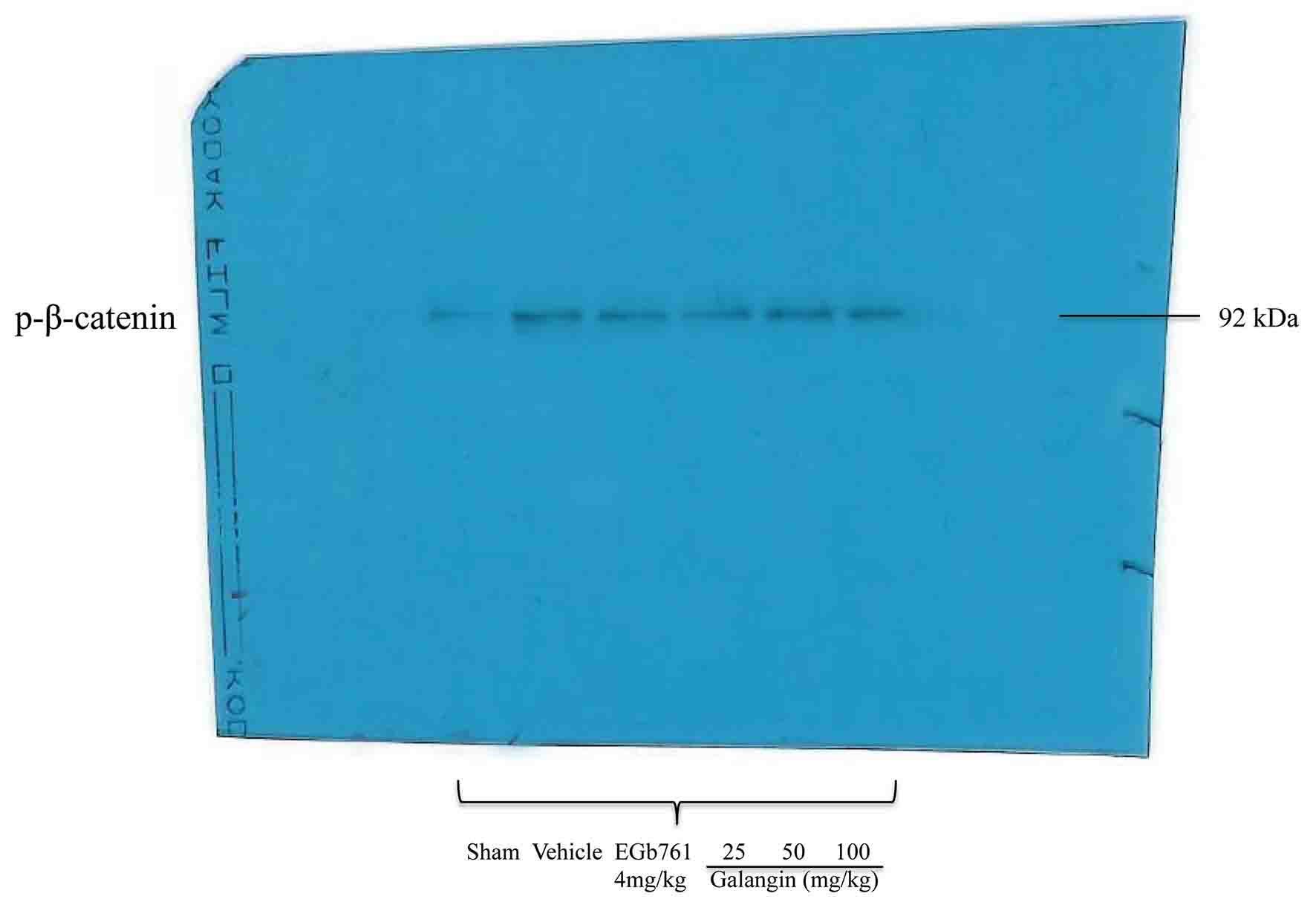


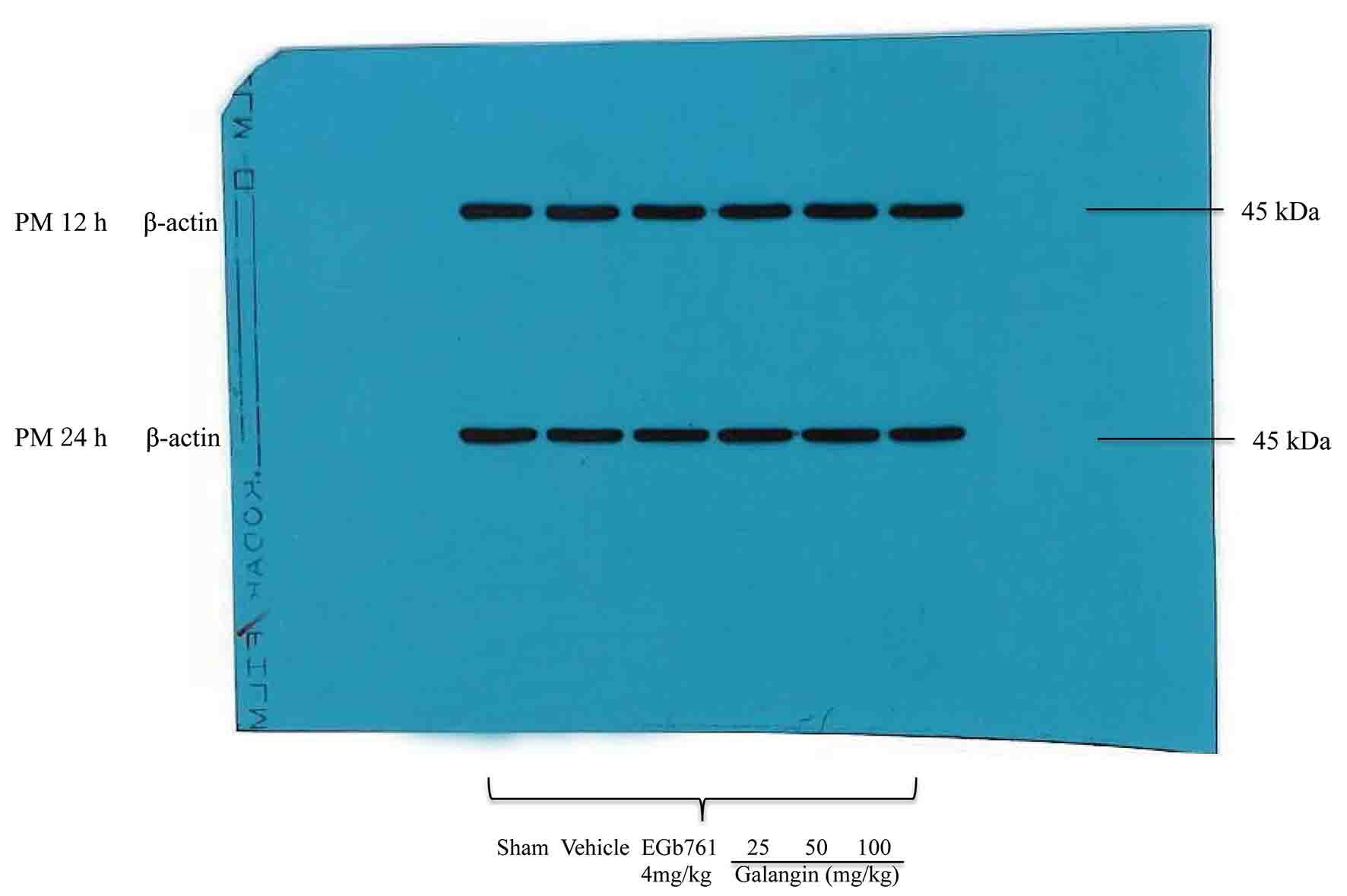


AM, 24 h


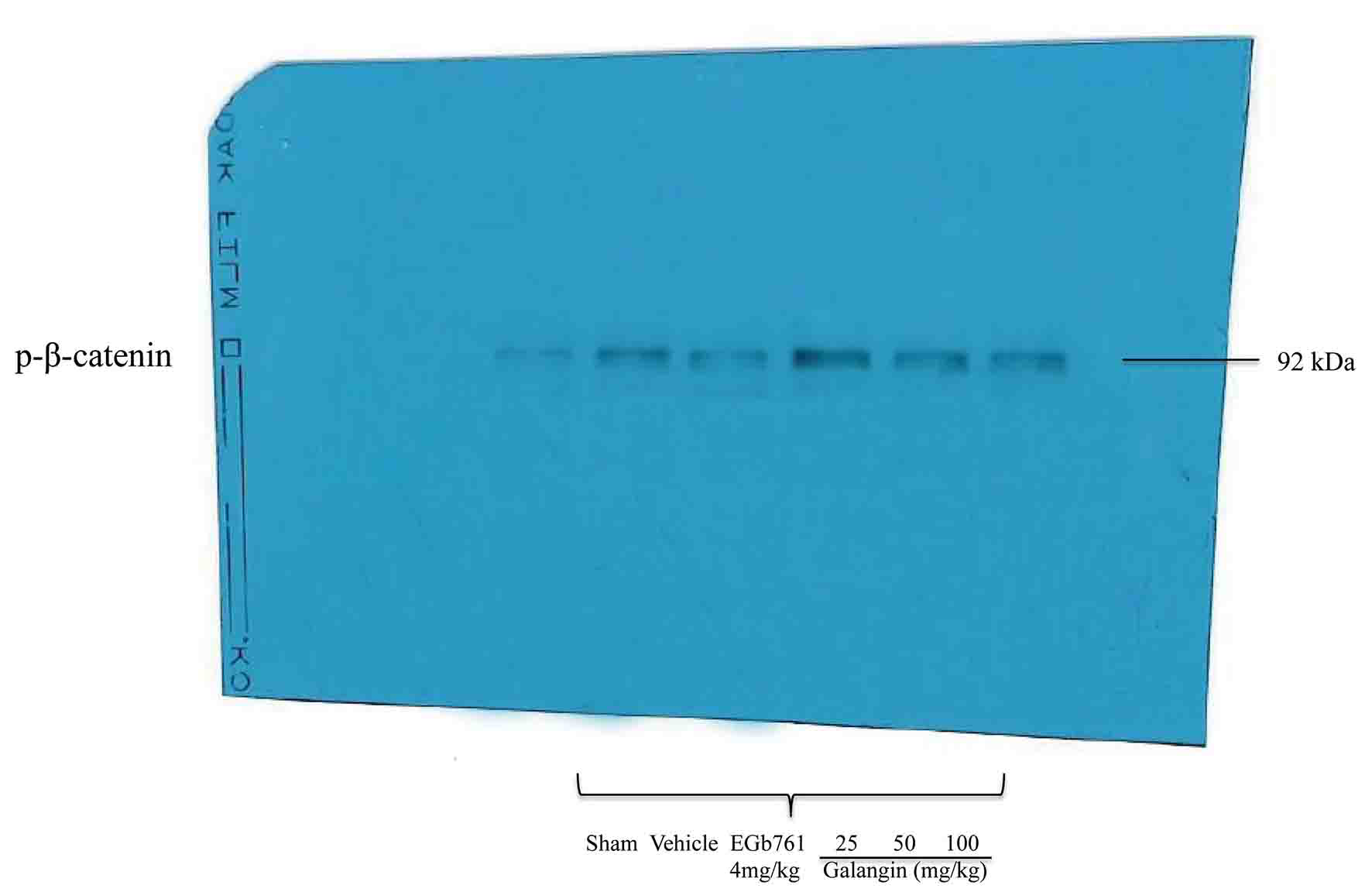


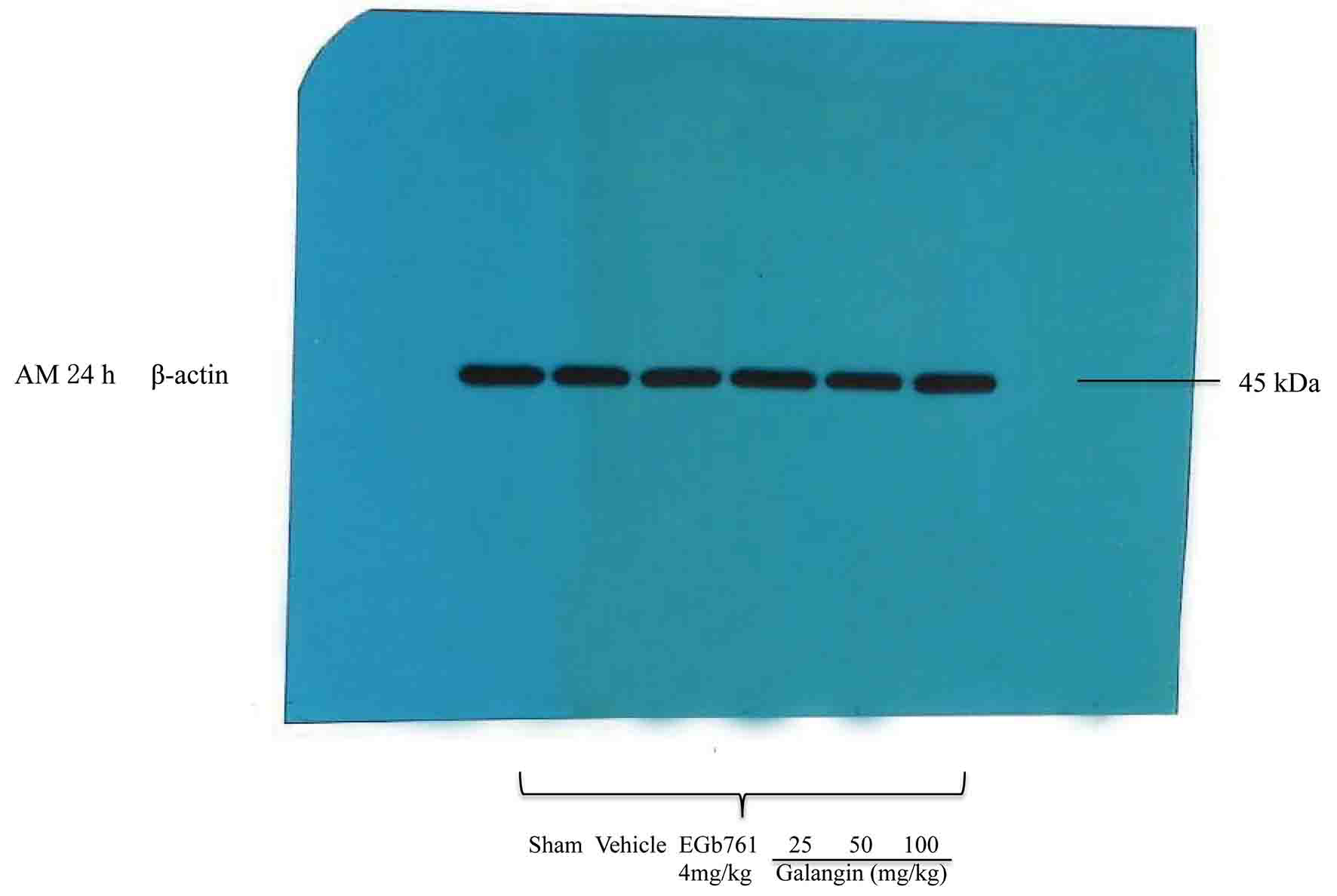


β-catenin:

PM, 12 h


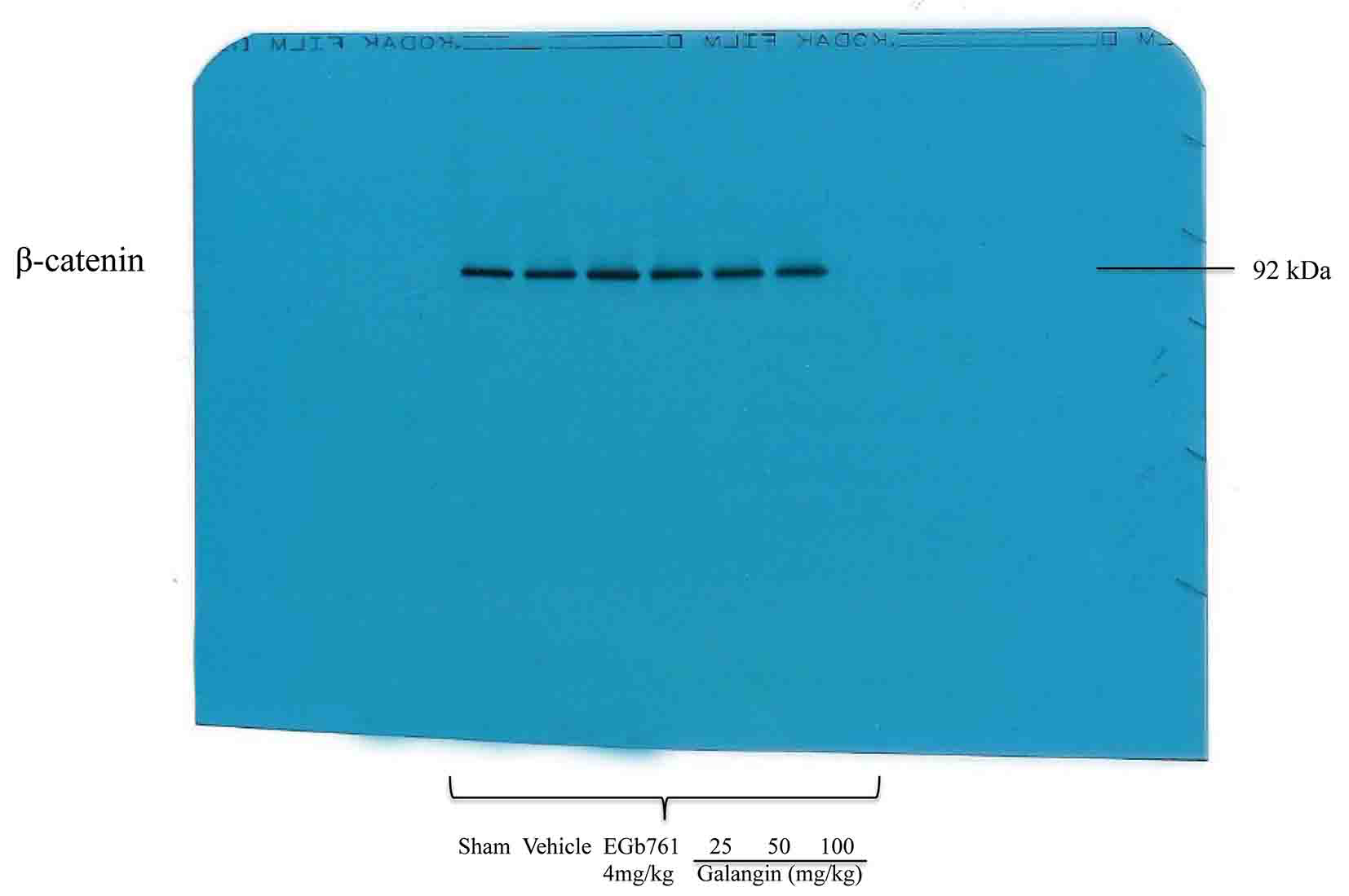


PM, 24 h


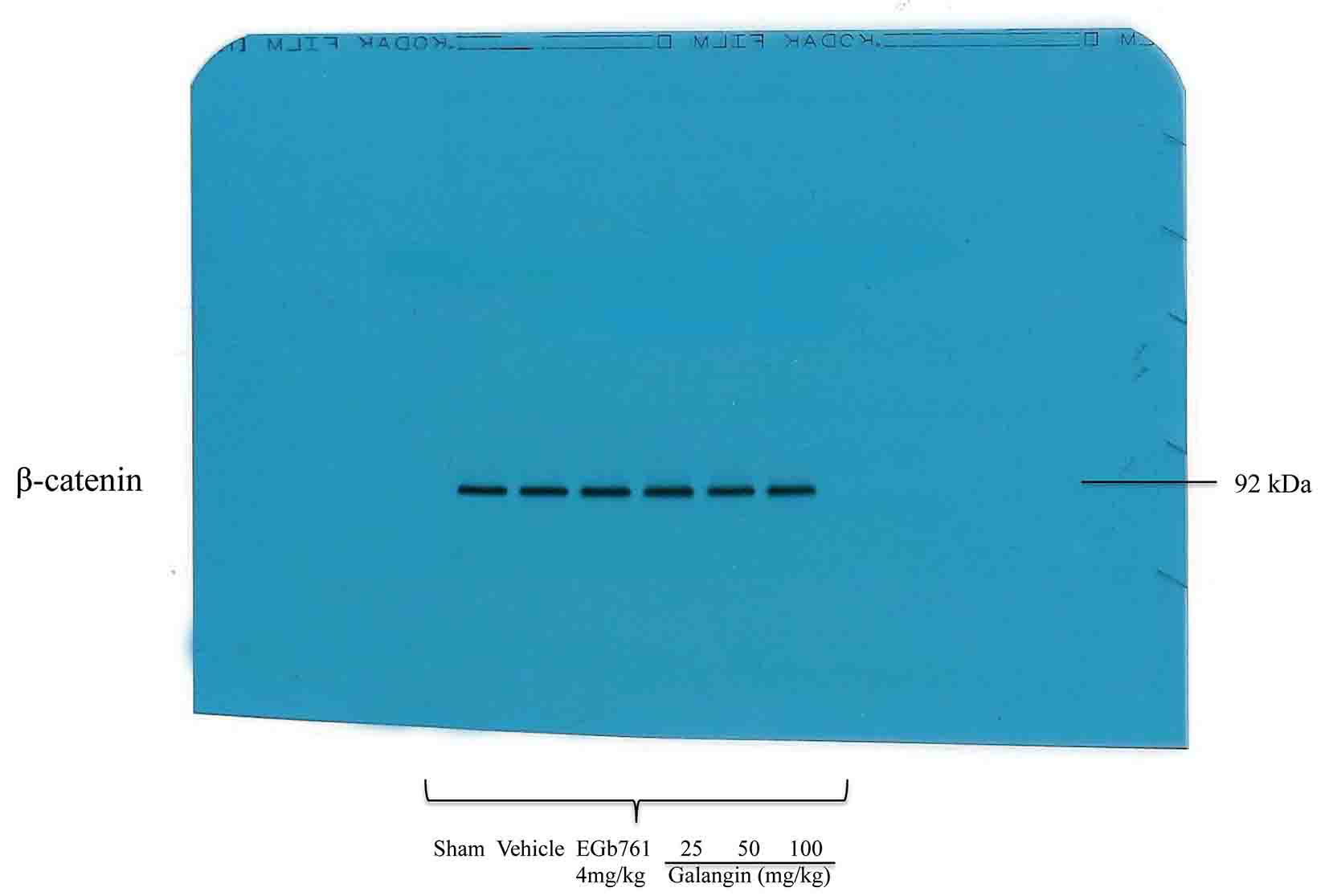


AM, 24 h


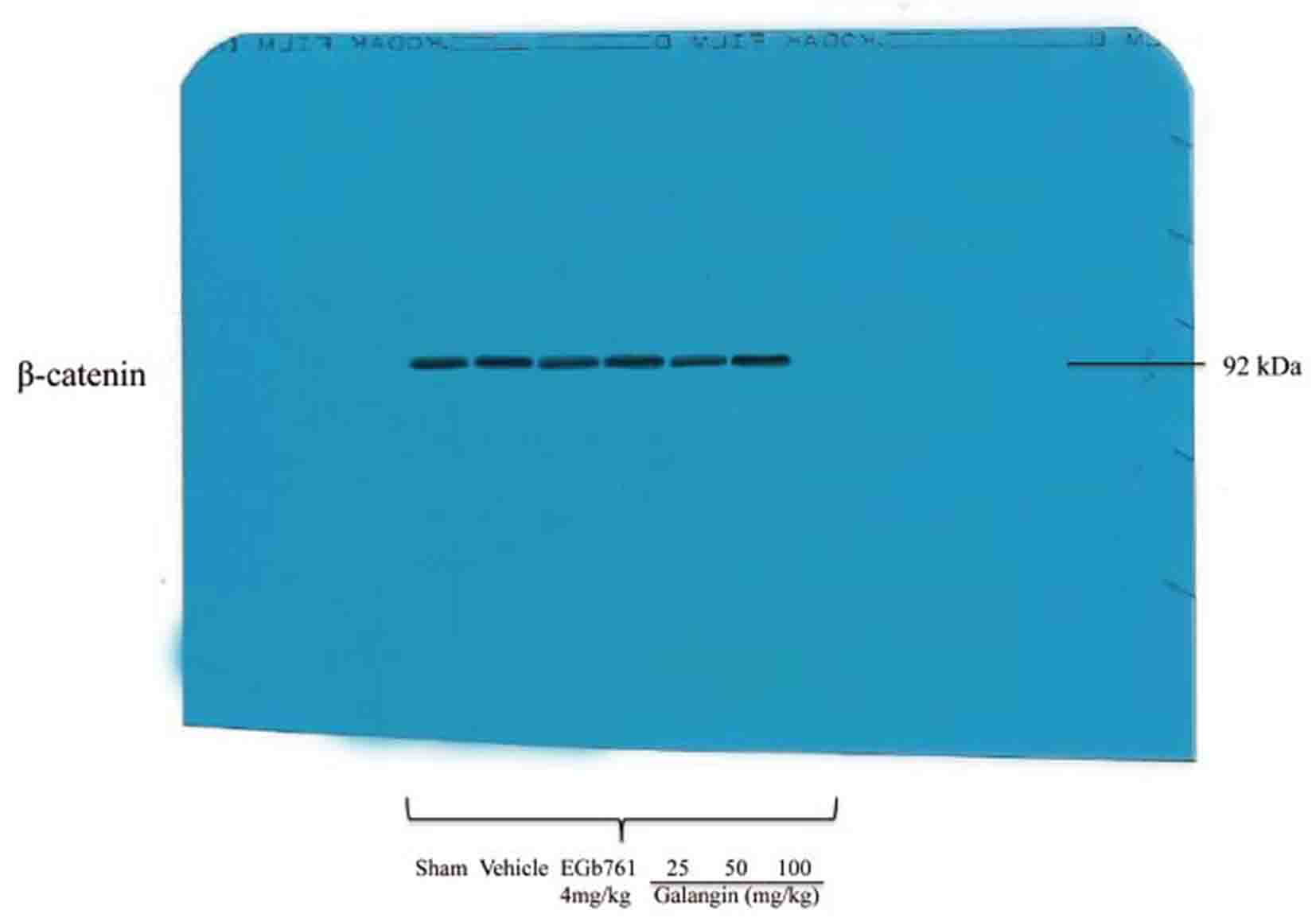


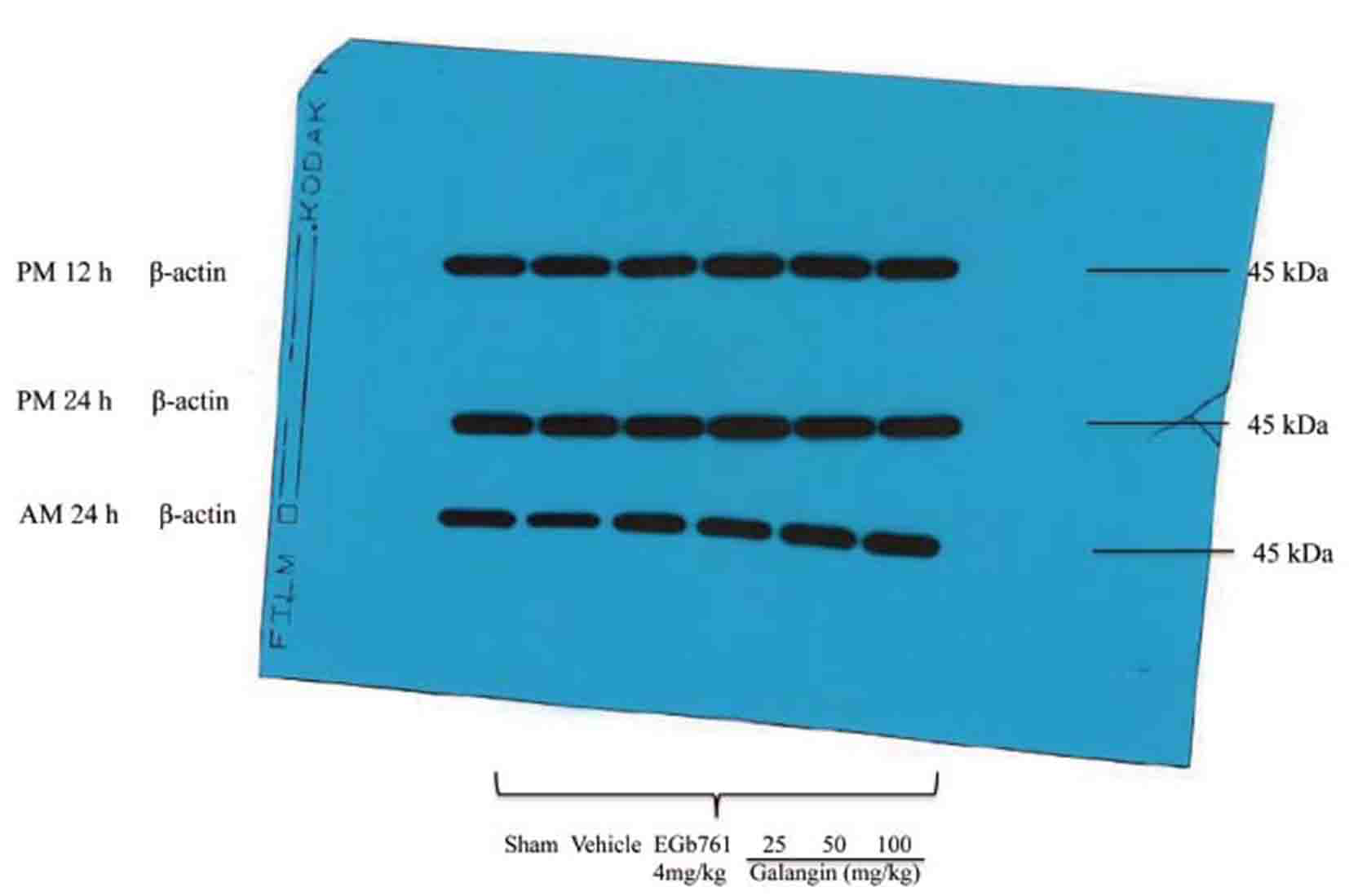


p-Smad3:

PM, 12 h


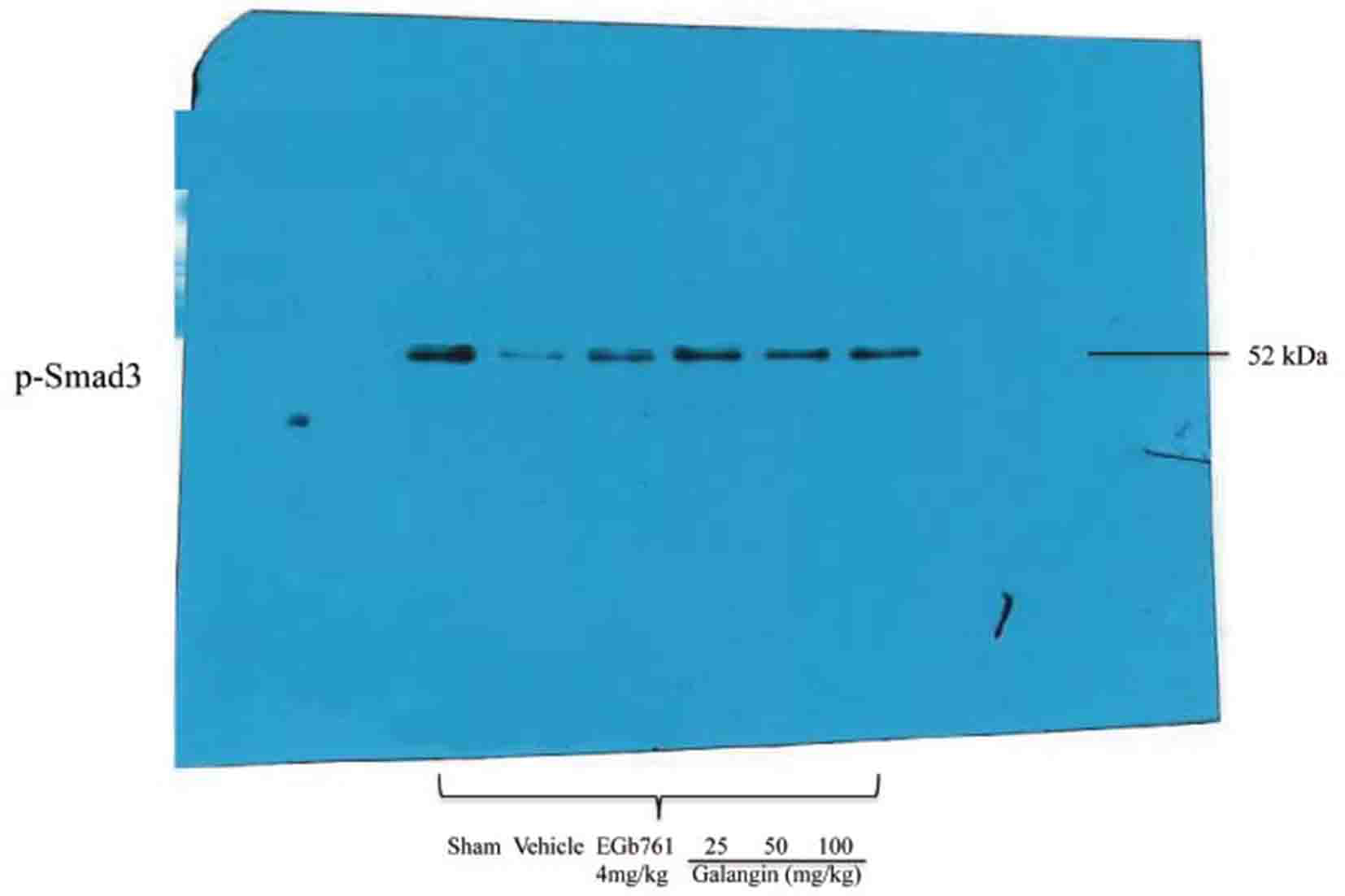


PM, 24 h


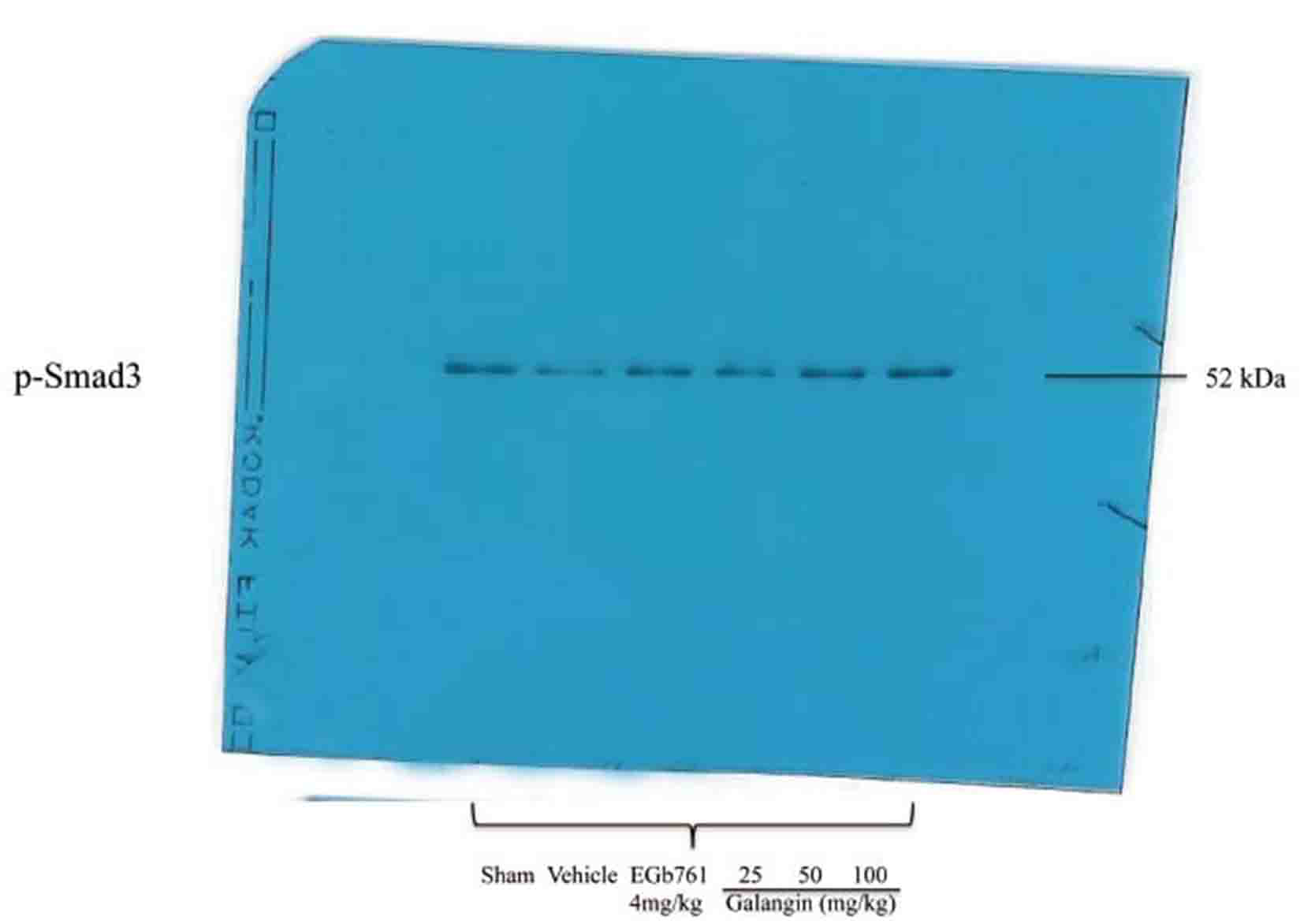


AM, 24 h


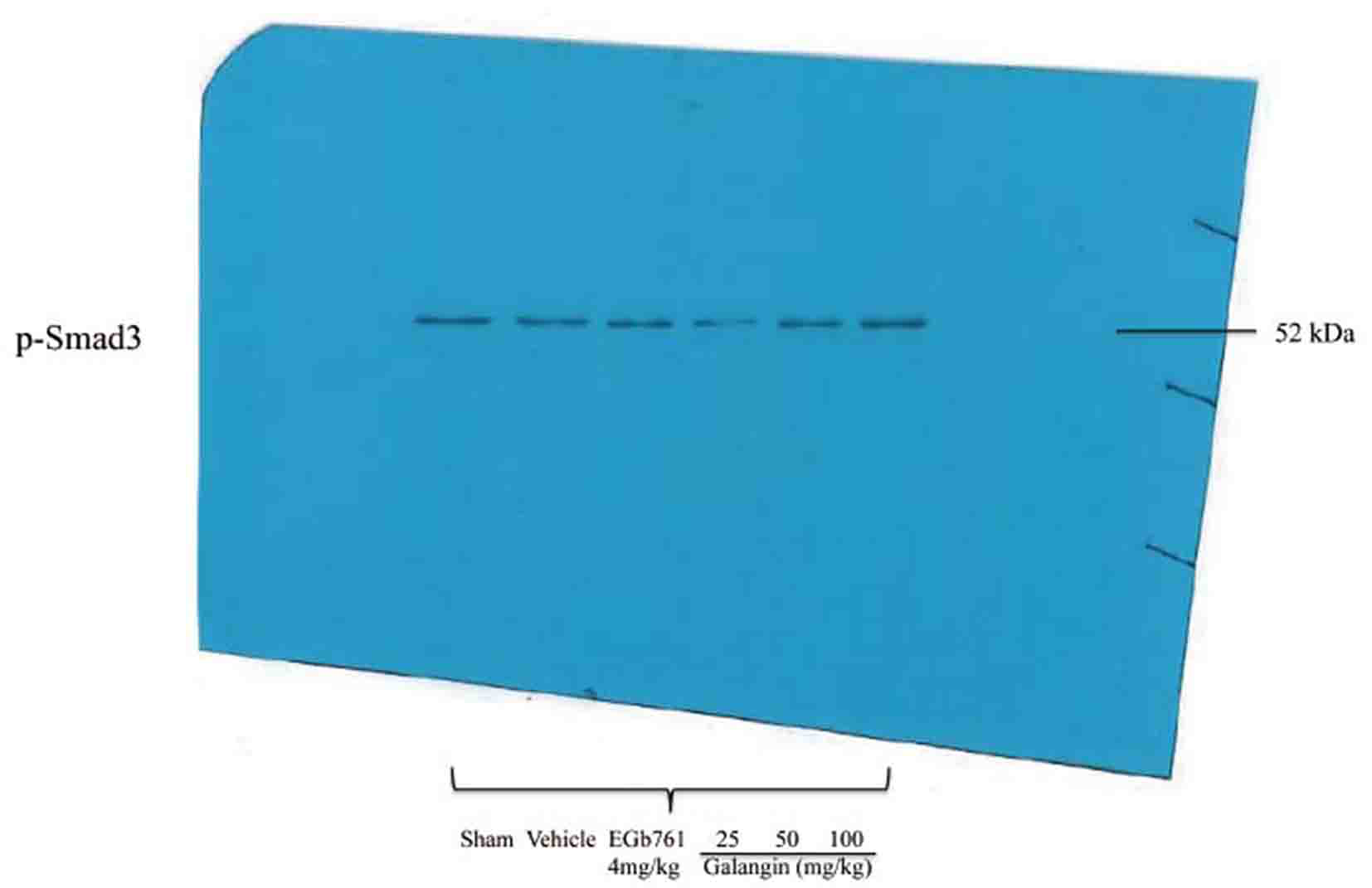


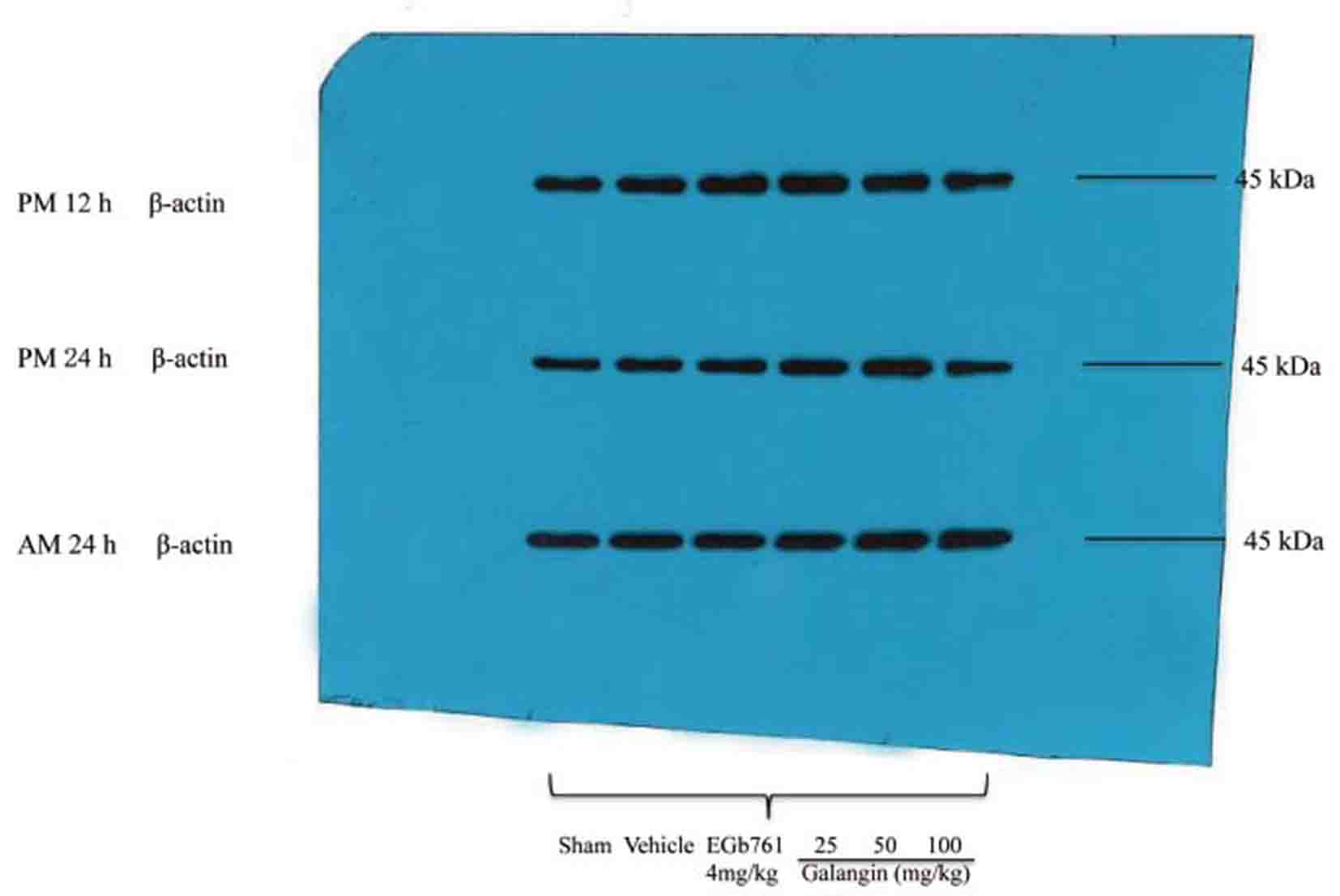


Smad3:

PM, 12 h


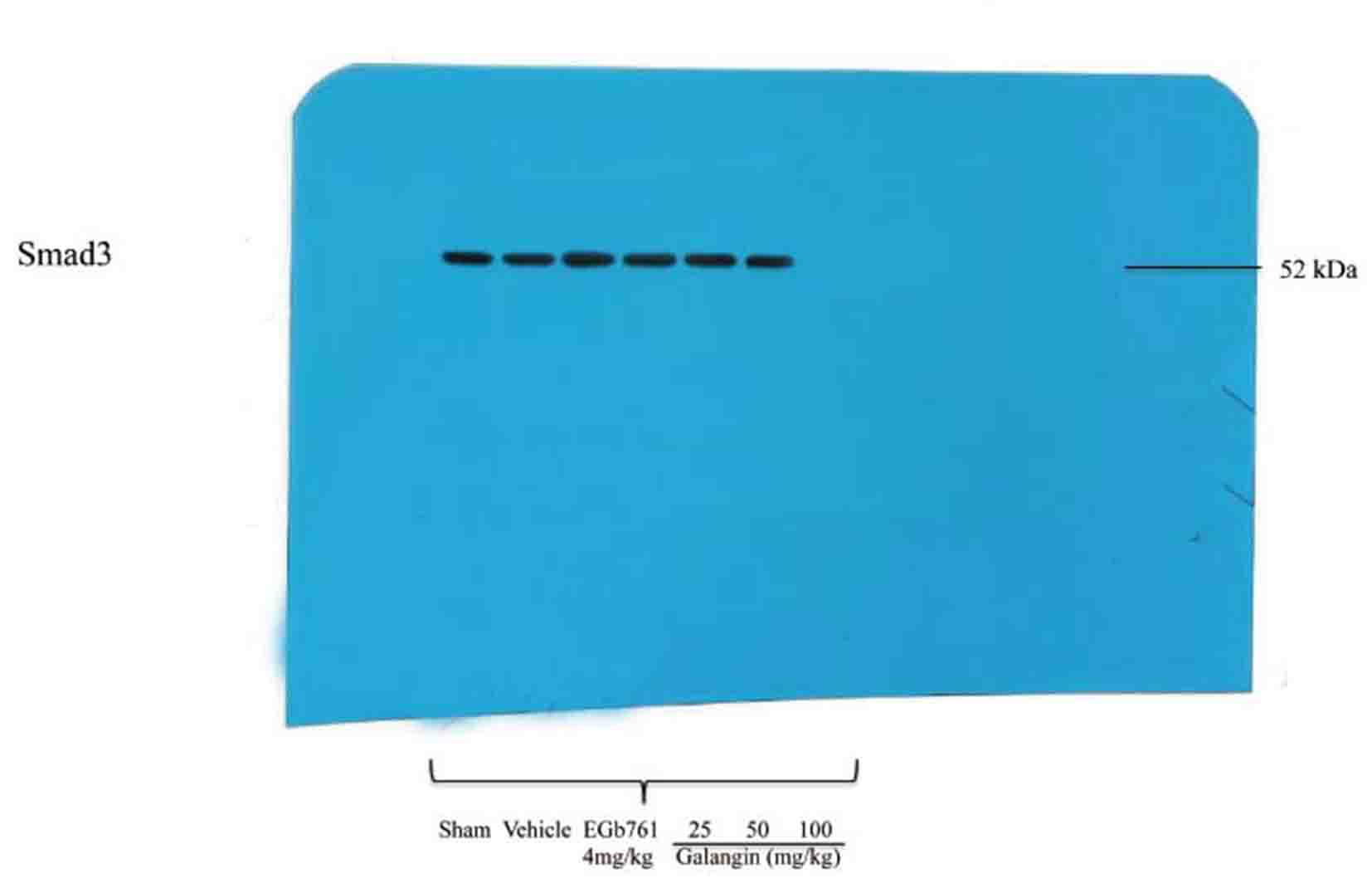


PM, 24 h


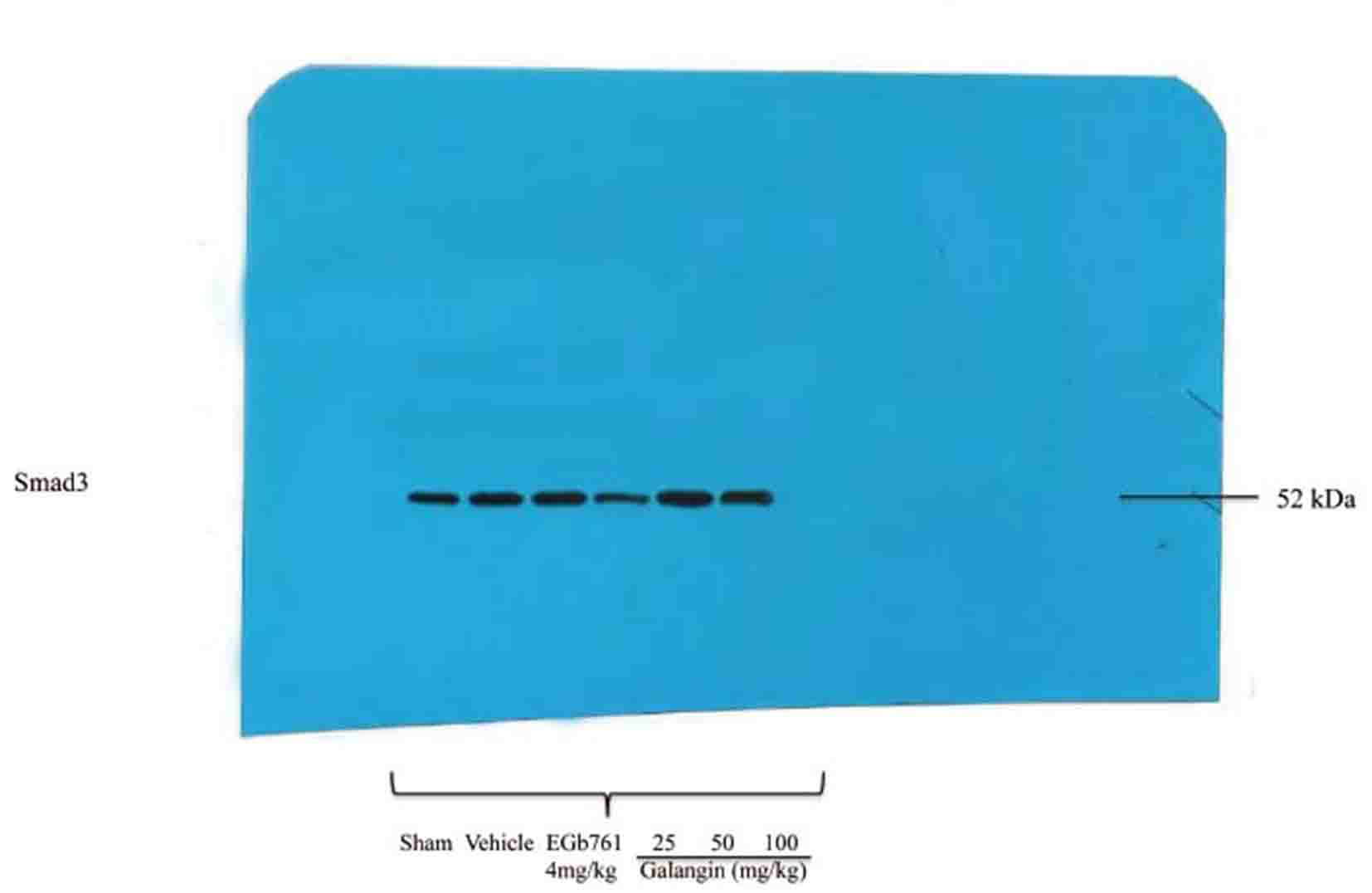


AM, 24 h


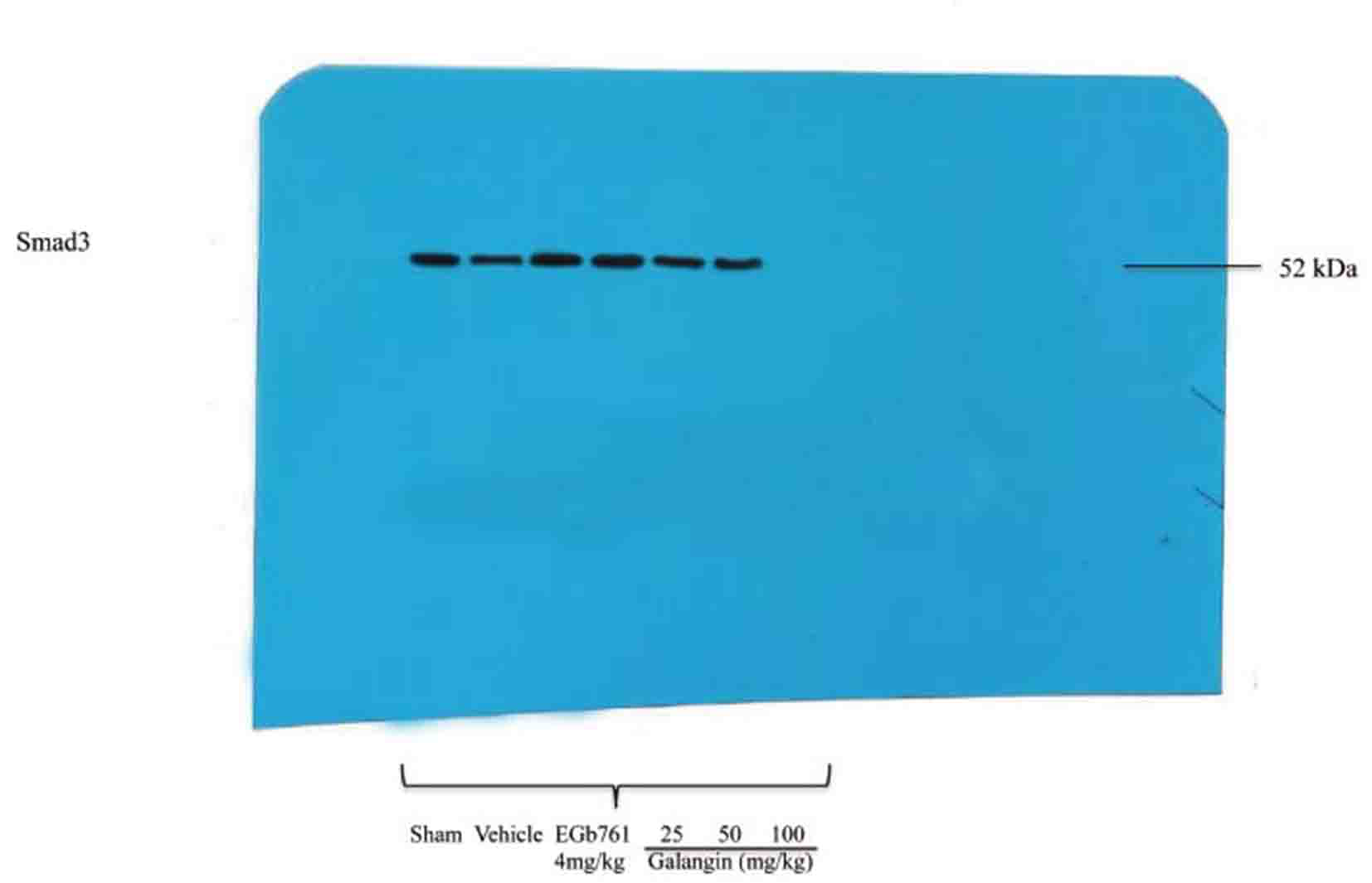


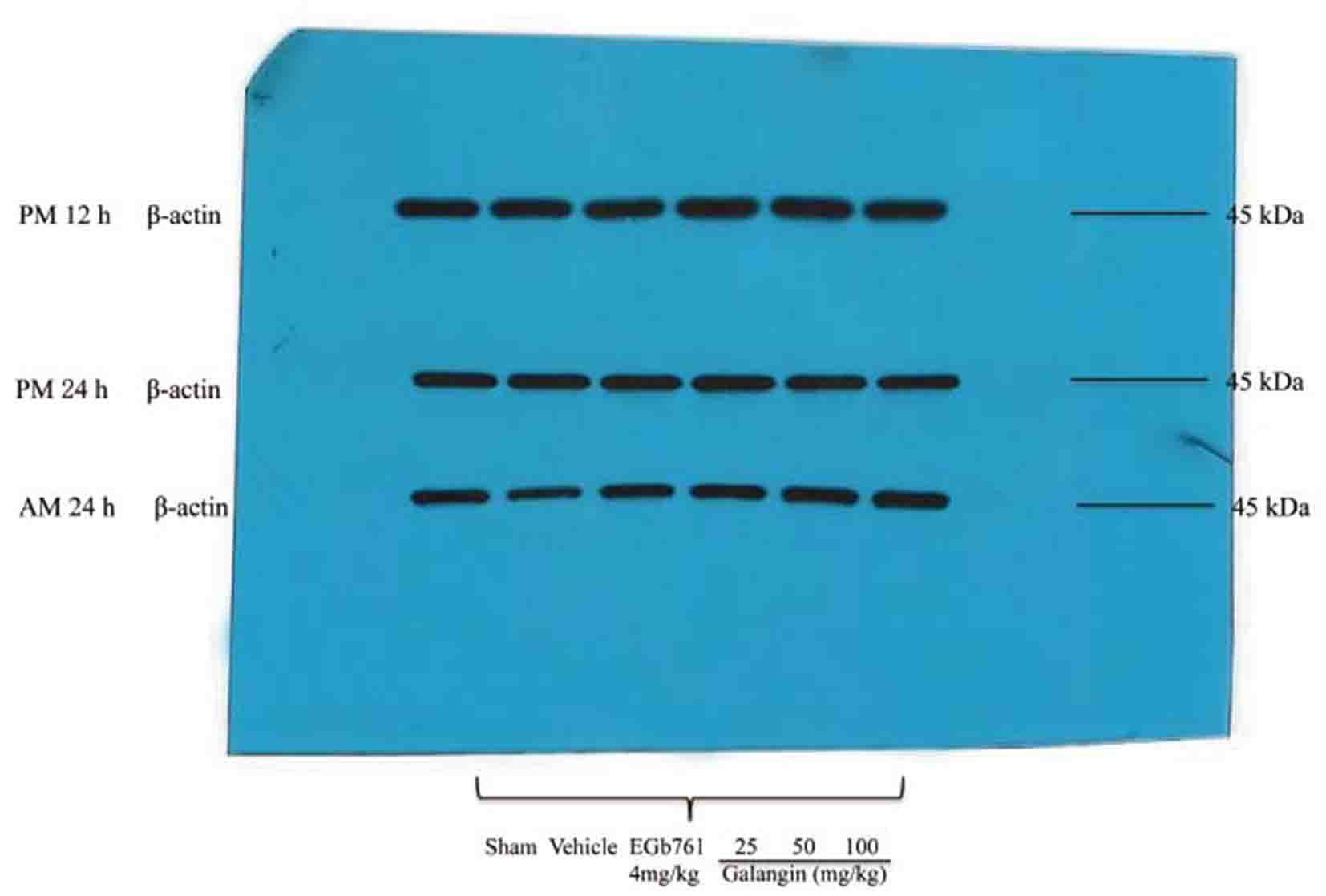


1. ^a^ Chuanhong Wu, Jianxin Chen and Chang Chen contributed equally to this work.

   ^*^ Correspondence author:

   Shaojing Li; Address: Institute of Chinese Materia Medica, China Academy of Chinese Medical Sciences, Beijing 100700, China. E-mail: [shaojingli2004@126.com](mailto:hongjun0420@vip.sina.com).Tel: +86-10-64012991.

   Huihui Zhao; Address: Beijing University of Chinese Medicine, Beijing 100029, China. E-mail: [zhaohh@bucm.edu.cn](mailto:zhaohh@bucm.edu.cn). .Tel: +86-10-64286283. [↑](#footnote-ref-2)
